# The keys to learning for university students with disabilities: Motivation, emotion and faculty-student relationships

**DOI:** 10.1371/journal.pone.0215249

**Published:** 2019-05-22

**Authors:** Anabel Moriña

**Affiliations:** Department of Teaching and Educational Organization, Universidad de Sevilla, Seville, Spain; Università degli Studi di Perugia, ITALY

## Abstract

The affective-emotional dimension may constitute a key element in teaching and learning processes. It is linked to relationship between faculty and students and may help foster students with disabilities’ motivation to learn and remain at university. This is the approach adopted in this article, which aims to fill a gap detected in the literature, since very little attention has hitherto been paid to motivation, emotion and the importance of faculty-student relationships in the learning processes of students with disabilities. In this study, 119 faculty members from 10 Spanish universities who engage in inclusive practices in all areas of knowledge recounted, in response to questions asked during a semi-structured interview, how they motivated and related to their students. The conclusion reached is that students with disabilities are more motivated than their fellow classmates, meaning that very few extra actions need to be taken to engage them in the learning process. Nevertheless, participants reported having a knowledge of strategies based on motivation and emotion and using them to develop a sense of belonging among students, thus fostering their learning.

## Introduction

The engagement of groups not traditionally represented among the university population, such as people with disabilities, is becoming an increasingly widespread object of study [1]. Student retention and academic success are not just the responsibility of students themselves. Universities also have the obligation to guarantee the necessary conditions and opportunities to ensure that all students can engage and learn [2]. Indeed, inclusive education is currently being recognized as an indicator of the quality of education systems. Various authors [3, 4] and international bodies [5, 6] have underscored the need for universities to become more inclusive, not only because this benefits students with disabilities, but also because it has a positive impact on other non-traditional learners and the entire student body [4, 7].

It is important that universities engage with students with disabilities. Not only because their access is getting wider and wider or because inclusive education is considered as an indicator of quality. It has also been studied that the presence of students with disabilities contributes to the development of a better university, as it requires transforming practices and developing attitudes and actions sensitive to disability [1]. In addition, for students with disabilities, the university is an opportunity, an experience of empowerment and is considered as a vehicle to improve their quality of life [7, 8].

### Faculty members: A key factor for inclusion of students with disabilities

While important, increasing both the number of non-traditional learners accessing university education and the resources available to them are not sufficient [8]. Several studies have concluded that faculty members play a crucial role in the inclusion of students with disabilities [9, 10]. The implementation of inclusive learning, teaching and assessment in the university is recommended in most works [11, 12]. Other studies, although less frequent, focus their attention on faculty-student relationships, both within and outside the classroom, as well as on the emotional connection generated between the two as a means of fostering student motivation and engagement, particularly among the most vulnerable learner groups [9]. These relationships are linked to the need to develop a sense of belonging to the university in terms of connectedness to the institution. Indeed, inclusive education not only refers to curricula and assessments designed to engage students in meaningful, relevant learning that is accessible to all [11], it also seeks to ensure that students feel themselves to be valuable members of the university community, members who truly belong and whose contributions are appreciated [13]. Thus, inclusive education engages students and generates a sense of belonging, which is essential to preventing dropout. This feeling is characterized by regular contact and a perception by students that their interpersonal relationships are stable, ongoing and based on affection. Indeed, Thomas [2] found that students with disabilities who did not feel they had a good relationship with academic members of staff were more likely to drop out. Therefore, the stronger the sense of belonging, the more likely learners are to remain at university until they have completed their degree [14].

### Teaching and learning: The fundamental nature of the affective-emotional component in the inclusion of students with disabilities and “all” students

To contribute to student learning, it is important to ensure not only effective teaching methods, but also positive interactions between faculty and students, concern by faculty about students, personal connections and respect [9, 15]. Thomas [2] identifies a series of elements which help retain students with disabilities at university, including practical curricula which are useful to future professionals; ensuring that learning and teaching enable peer interactions and active engagement; ongoing assessment and feedback; personal tutoring resulting in a close relationship, which in turn enables customized monitoring of each student; and finally, good faculty-student relationships. In other words, it is important for students to get to know their faculty members and to feel confident in asking them for help if required.

According to this approach, the emotional-affective component of teaching and learning is vital [16]. As Scott et al [17] argue, a class's inclusiveness depends on the type of interactions which take place between students and faculty, and in this sense, faculty members are encouraged to make personal connections with their students and use motivating strategies.

It has been accepted for some time, in research involving students in general, that cognition, motivation and emotion are the three factors involved in human mental operations [18]. For student motivation, as Almarghani and Mijatovic [19] state, both the learning climate and the teaching approach can influence engagement. These authors attach great importance to faculty empathy and the strategies they use to support their students. For their part, Klauda and Guthrie [20] conclude that there is a positive correlation between motivation and engagement. In the specific case of students with disabilities, some authors believe these learners have a stronger intrinsic motivation than the rest of the student body [21]. Moreover, humor, respect and social support in the classroom nurture students’ academic experience.

Over recent years, several studies have called for more attention to be paid to the emotional dimension of learning [22, 23]. Indeed, some authors have even referred to the need for a pedagogy of emotion [24] or a pedagogy of the heart [25]. According to these authors, emotions influence students' cognitive resources, motivation to learn, learning strategies and self-regulation. However, as Regan et al. [26] and Walker and Palacios [24] argue, insufficient attention has hitherto been paid to this dimension of the learning process.

### The need for faculty-student relationships to connect emotionally with all students

Emotions are a key part of learning. Faculty members should therefore take them into account and strive to emotionally connect to students [27, 28]. Quinlan [15] claims that emotions are a vital aspect of relationships, arguing that the way we feel with and about others is fundamental to the quality of the relationships we establish. Therefore, emotions are important in university teaching and learning processes, as an aspect which enriches the social and relational experiences underpinning student development. Research has shown that emotions and relationships are closely intertwined [29]. When students perceive that faculty listen and show immediacy through behaviors which generate a sense of closeness, they experience learning more positively, feel emotionally supported and are more likely to express their own emotions in a more authentic manner [30]. These authors highlight the importance of the relationship faculty establishes with their own subjects (passion, interest), as well as roles linked to student care (empathy, respect). In sum, student interactions are often emotionally-charged and faculty-student interactions are the most frequent source of faculty emotions [29]. Based on these studies, it should be noted that there are no differences in the importance of emotional connections between students with disabilities and other students. Also, for them, these are fundamental for learning [2]. Lastly, research has shown that high-quality relationships have a positive impact on people in terms of motivation, social competence and general well-being [14]. Personal relationships can be considered a prerequisite for successful learning among all students, although particularly among learners who are at risk. Only a small number of studies focus on personal relationships in higher education, and that further research in this field is required [14].

### The voice of students with disabilities: Faculty who contribute to their inclusion

Finally, evidence has been reported by studies seeking to give voice to students with disabilities regarding the specific profiles of faculty who contribute to inclusion [31, 32]. The profiles described by these studies depict people with a positive attitude towards disability that are also flexible, approachable, understanding and ready to help. The faculty members most appreciated by students with disabilities are those who are not overly theoretical, but rather explain using examples and incorporate innovations into their classrooms. Some informants also highlight faculty who have a thorough understanding of their subject and know how to transmit their knowledge, while others value their personal characteristics, i.e. how they relate to students (are they approachable or distant?), or as the students themselves put it: their "human aspect". Umbach and Wawrzynski [33] conclude that students learn more when faculty use active and cooperative learning techniques, interact with their students and strive to engage them in the learning process, and Griffiths [32] highlights that showing respect for everyone and listening to all students are two fundamental aspects. Therefore, one of the main factors fostering inclusion is for faculty to get to know their students and to show an interest in them. For students, it is important to have a sense of belonging to the university. One element, which enables this feeling to develop, is for faculty to teach with enthusiasm and passion, and to encourage students to participate in class [34]. In a study conducted by Stein [35], when asked what elements contributed to their academic achievement, students identified (among others) the fact that faculty were concerned about them (responded to their messages, were available during tutorials, made reasonable adjustments, etc.). Lipka et al. [36] also reached similar conclusions, highlighting empathy, caring and approachability as elements valued by students with disabilities.

In sum, the theoretical framework presented in this article argues that methodological strategies are not enough, and that an affective-emotional component is also required during learning. This component is linked to the close relationships maintained between faculty and students, which ultimately result in greater student motivation to learn and remain at university. This is precisely the approach adopted in this study, which aims to fill a gap detected in the scientific literature, since very little attention has hitherto been paid in higher education to motivation, emotion and the importance of faculty-student relationships for learning, and even fewer studies have focused on these elements in relation to students with disabilities [29, 24]. The research questions asked are therefore as follows:

- What strategies are used to motivate students with disabilities?
- How do faculty-student relationships and emotional connections influence learning processes?
- What strategies are used to foster these relationships?

## Method

### Participants

The results of this study are located within the framework of a broader research project which aims to study what university faculty who engage in inclusive practices do, and how and why they do it (Inclusive Pedagogy at the University: Faculty Narratives ref. EDU2016-76587-R). This study was reviewed and approved by the ethics committee of the Spanish Ministry of the Economy and Competitiveness. To guarantee the suitability of the sample, only those faculty specifically nominated by students with disabilities were included. Two strategies were used to access the sample. Firstly, the support services at the 10 participating universities sent out information about the project to students with disabilities from all areas of knowledge, requesting their voluntary cooperation for nominating faculty who, in their opinion, practice inclusive education. We also used the snowball technique [37], asking this question directly to students with disabilities whom the research team already knew from previous projects. Colleagues in a position to know students with disabilities were also asked to collaborate.

Once the students had been identified through the channels outlined above, the support services or research team contacted them via email to explain the aims of the project and ask for their collaboration. Specifically, students were asked to recommend any faculty member who had made them feel included in the subject they were studying during their time at university. Certain characteristics were suggested to help students identify these faculty members: they believe that all students have potential; they facilitate learning processes; they engage in active teaching, using different methodological teaching strategies; they are concerned about their students' learning; they are flexible and willing to help; they motivate students; they are approachable and foster interactions between students; and they make students feel like important members of the class.

### Data collection instruments

The research team designed a semi-structured interview script that was discussed and piloted with 15 university faculty members not participating in the study. All necessary modifications were made in accordance with the pilot participants’ suggestions and recommendations. The mean duration of each interview was one hour thirty minutes. During the interview, participating faculty were asked a range of different questions. For example, what are the key elements for ensuring engaged, motivated students? Do you do anything different to specifically motivate students with disabilities? If so, why and how? And in relation to faculty-student relationships, do you consider them important? Why/why not? What type of strategies do you use to foster a good relationship with your students? How do you think this relationship influences students' learning? Do you do anything different to specifically engage students with disabilities?

The majority of interviews were held face-to-face (n = 89). Nevertheless, 18 faculty members conducted their interviews via Skype and 12 did so over the telephone, since it was impossible for them to attend in person. Audio recordings were made of all interviews.

### Data analysis

The information was transcribed and analyzed using a progressive qualitative data analysis technique, in which a system of inductive categories and codes was generated which enabled meaning to be attached to the information gathered [38]. All the transcribed interviews were analyzed by at least two people. The whole research team discussed those data on which doubts arose in relation to their analysis until a consensus was reached. To help the team handle the large quantity of information collected, the computer program MaxQDA14 was used during the data analysis process.

### Ethical issues

This research guaranteed that all information would be treated anonymously and confidentially. In the case that any participant did not wish to continue participating, they were informed that such data would be destroyed and not included in the research report. Faculty members gave their written consent to being recorded. All the information gathered, once transcribed, was returned, so that the participants showed their agreement or disagreement with the transcription made.

## Results

The results are organized in four sections to answer the following questions: What is the profile of the participants in the research? What strategies are used to motivate students with disabilities? How do faculty-student relationships and emotional connections influence learning processes? And what strategies are used to foster these relationships?

### What is the profile of the participants in the research?

The final sample group comprised 119 faculty members from 10 Spanish universities. Of these, 24 (20.16%) taught Arts and Humanities (Participant 1 -P1- P24), 14 (11.76%) taught STEM (Science, Technical, Engineering and Mathematics) (P25-P38), 16 (13.44%) taught Health Sciences (P39-P54), 25 (21.01%) Social and Legal Sciences (P55-P79) and 40 (33.61%) Education (P80-P119). As regards gender, 58.33% were men and 41.66% were women. The majority was aged between 36 and 60, with seven (7.78%) being less than 35 years of age and four (4.42%) being over 60. Most (68.35%) had over 10 years' experience, with only six (6.25%) having less than 5 and 24 (25.4%) having between 5 and 10. All had experience responding to the needs arising from disabilities. Of these, the most frequent were sensory disabilities, i.e. visual and hearing impairments (40.97%), followed by physical (23.68%) and mental (18.79%) disabilities, poor health (10.52%) and learning difficulties (6.01%). Finally, four of the 119 participants had a disability themselves.

### What strategies are used to motivate students with disabilities?

The majority of participants in this study shared the belief that the actions taken to motivate students with disabilities were no different from those taken in relation to other students, and indeed, no specific strategy was required to engage these students in the learning process. This was because students with disabilities were already more motivated to study at university than the rest of the student body:

*P116*: *They are already incredibly motivated*. *If I were to motivate them any more*, *it would be too much*. *I just wish that students who do not have difficulties were half as motivated as those with disabilities*. *I don't have to do anything special with them*.

Only 18 faculty members claimed to do things differently, saying that they used a series of strategies to help students with disabilities learn. They explained that they tried to give them confidence and show an interest in them, making them feel like just another student, using positive reinforcement and, in some cases, giving them personalized attention during the learning process:

*P3*: *I try to reassure them*, *give them confidence*, *explain to them that I'm here for them if they need me and that they shouldn't be concerned about the subject*, *that the most important thing is basic knowledge*.

For the faculty participating in our study, showing affection for students was a strategy which contributed to their inclusion. This demonstration of affection transcended what happened in the classroom itself, and some faculty members even commented that, if necessary, they spent time with students with disabilities in other, non-academic settings. Nevertheless, they played down the importance of this, considering it something natural, something they would do for anyone:

*P17*: *Well*, *with Diana–fictitious name-*, *I always tried to be aware of her*. *I made an effort to establish a certain rapport with her*, *although to be honest she made it very easy*. *For example*, *I sometimes took her home*, *or went with her to the metro*, *or I gave her a hand*, *just like I'd do for anyone*.

In general, participants commented that the strategies they used to motivate other students were equally effective with students with disabilities. Although they said it was important to include actions designed to foster student engagement and learning when planning teaching and learning processes, they also acknowledged the existence of prior issues that affect motivation. The principal one was students' previous motivation in relation to their chosen degree course. In other words, participants felt that studying something for which they felt a vocation strongly influenced students' engagement. In many cases, students were not studying subjects that really interested them, and this did not help their commitment to learning. However, when they did feel a vocation, teaching them was easy:

*P2*: *One main element is to ensure that you study something you really enjoy*. *It needs to be a vocational choice*. *That way success is guaranteed*.

However, participants stated that students' motivation in relation to their course was not the only important thing. Faculty motivation was also a key element. They stated that it was important for faculty to enjoy teaching, to become involved and feel part of the process. They also said it was important for them to feel and show passion for teaching:

*P38*: *The most important thing is for students to see that you are motivated*, *it is important for them to see your passion*.

In addition to this motivation, participants also stated that it was vital for faculty to be well-trained, have well-prepared classes and to have a thorough understanding of all aspects of the subject being taught:

*P15*: *It is important for them to see that you have a thorough knowledge of your subject*. *They need to feel you are in complete control and know what you are doing at all times*. *They should never see you doubt or hesitate*, *you should be completely familiar with all the ground you cover*.

Nevertheless, in addition to the vocation felt by students and faculty, participants also mentioned several strategies that can be used to make subjects more attractive. The majority coincided in pointing out a series of key elements for motivating students, not only those with a disability: classroom climate, linkage of contents with future professional, active methodologies, varied use of activities and resources, and ongoing feedback.

a) Classroom climate

The first was linked to classroom organization and the importance of generating a good classroom climate in which students feel welcome and at ease, with a sense of forming part of and belonging to the group. To achieve this, participants said it was necessary to create a relaxed, welcoming atmosphere, and many said they used humor to make students feel that learning can be fun and to help them feel comfortable and at ease in class.

*P44*: *I think that when you create a very empathetic*, *relaxed*, *warm*, *welcoming and motivating class climate*, *then even when students don't really like the content of what you are teaching*, *they end up becoming engaged in the subject*.

b) Linkage of contents with future professional activities

From an academic perspective, they said it was vital to link the subject matter of the course with students' future professional activities, stating that contents should be both useful and practical. Participants believed it was important for students to feel that they were learning, and that the contents being taught would be useful to them in their future careers.

c) Active methodologies

Another strategy was linked to methodologies. Participants explained that in order to motivate students, it was important to use active strategies that foster participation and engagement. They said that classes should not be passive

*P43*: *I believe it's vital for students to feel like active agents in their own learning processes*. *They need to feel like the protagonists of this process and to become engaged in it*.

d) Varied use of activities and resources

On the order hand, the participant explained that they used different types of activities and resources that were more dynamic in nature. The reasons given by participants for using different types of activities and resources were not only related to encouraging a greater degree of student engagement. They also argued that a diverse range of resources in itself enabled them to get their message across to more students, since everyone learns differently. Therefore, a variety of different activities and resources should be used:

*P13*: *There are many different types of students*, *some are more visual*, *others more* … *so if you vary the type of activity you use*, *you can try and include all of them*. *You shouldn't always use the same kind of activity*. *It's important to understand that there are different types of learning*, *and different types of students*. *So*, *that's what we try and do*, *right*? *We try to capture this diversity*.

e) Ongoing feedback

Participants stated that planning the subject also included giving ongoing feedback to guarantee that students had a clear idea of their progress at all times and understood those aspects of the learning process in which they needed to make a more concerted effort. They also said it was important to provide positive (not just negative) feedback. They talked about the importance of this feedback being immediate, stating also that faculty should respond as promptly as possible to queries sent by email or over the platform:

*P42*: *Ongoing positive reinforcement when things are done well (which is almost always)*, *because it's important to recognize this*. *And when the work is mediocre or poor*, *then it's important to let them know too*, *so they can improve*.

Finally, it is worth noting that although in this study the majority of participating faculty were aware of and used strategies to promote student engagement and motivation, two of them claimed not to know any strategy and not to know how to achieve this aim, and another three said they thought it was difficult since student attitudes did nothing to foster learning:

*P6*: *It think it's getting more and more difficult*, *because the type of student you get these days at university is different* … *It's really hard*, *because they seem to have an ever decreasing capacity for work*, *less ability to relate to each other and there are more and more conflicts between them and less and less mutual support*.

### How do faculty-student relationships and emotional connections influence learning processes?

Almost all participants in the study believed that faculty-student relationships were vital. Only three participants failed to share this view, two because while they considered it important, they also saw it as impossible due to the high number of students in their classes, and the third because they did not believe it contributed anything to the learning process. All other participants, however, stated that faculty-student interactions were fundamental, because everyone has to learn to live together in the classroom and they were convinced that learning occurs best when those involved feel close to one another:

*P33*: *For me*, *it's very important for this relationship to be easy and congenial*, *and for there to be good communication*.

Some participants even claimed that this was their main resource for attending to the needs of students with disabilities, considering it to be even more important than the contents being learned. They explained that, on many occasions, faculty members who were approachable and concerned about their students, and developed close relationships with them, made a deep impression.

*P81*: *I think it's much more important than even what you can learn in class*.

*P25*: *We all remember at least one special teacher*. *Why*? *Not because they explained things better than anyone else but because they touched you in some way*, *because they were concerned about whether or not you really understood* …

For more than half of the faculty participating in the study, relationships were important because they fostered learning. Indeed, they stated that this was not only happening with the student with a disability, it was the same with the rest of the students. They thought that when faculty failed to connect with students, when they failed to establish a good relationship with them, learning was difficult.

*P110*: *If you don't establish a good connection*, *a good rapport*, *it doesn't matter how well you explain things*, *they won't learn*.

They linked relationships to the emotional component of learning, explaining that positive emotional contexts fostered this process. Participants stated that emotional connection was important, since emotions are vital for stimulating students, encouraging them to be "present" in teaching and learning processes and to commit to both themselves and the subject:

*P101*: *I remember a course I went on as a teacher*. *The first part of it really made an impression on me because some really good teachers came and one of them got us to do an activity*. *He said "let's see*, *think about the best teacher you've ever had in your life"*. *So we did*. *And almost all of us coincided in saying that the person we thought of was a good person*. *"Yes*, *but did you learn a lot too*?*" the teacher asked*. *And of course*, *the answer was yes*. *But it's about the emotional connection*. *If the emotional side of things works well*, *you learn more*, *you invest more in a subject and you learn more when you feel comfortable and you like the teacher*, *right*? *You engage with the subject*.

Other 37 participants stated that faculty-student relationships were important because they helped students trust the person teaching them, and this in turn gave rise to more fluent communications and encouraged students to express themselves freely. As a result, faculty had a better idea of their students' needs, and being aware of these needs enabled them to help students more effectively.

Moreover, fluent communications between faculty and students enabled mutual learning. According to participants, it was not only students who learned, but they themselves also, since they had the opportunity to hear what students had to say about different aspects of the teaching and learning process, and find out what was working and what was not, something which put them in a much better position to take steps to improve:

*P115*: *You get feedback*, *so you know whether or not they are understanding what you are teaching them*, *whether or not they like it*. *I think we all learn*. *We learn about how to approach teaching*, *and they learn contents and skills*.

### What strategies are used to foster faculty-student relationships?

Participants described a diverse range of strategies for fostering good faculty-student relationships, although they also stated that the strategies they used in relation to students with disabilities were no different from those used in relation to the rest of the student body. Only one participant claimed not to be aware of or use any strategy:

*P3*: *Well*, *I'd like to*, *but I don't really know how to go about it*. *I don't know how to reach out to a group of 40 students*. *I'm sure there are instruments*, *but I don’t know what they are*.

A) Closeness

The main strategy used by participants was to be warm and friendly, to tell students about their everyday life, to be approachable in class and available at all times, talking to students, paying attention to them and evincing an interest in their lives. In short, almost all the participants said that one strategy that never failed was to make students feel important:

*P51*: *Well*, *I normally ask them how they are before class starts*, *how they are getting on* …*I show an interest in them*.

b) Horizontal relationships

Other strategies were more related to the type of relationship established. 38 Participants stated that it was important to ensure horizontal relationships and to put themselves on an equal footing with students, treating everyone the same. They also mentioned that mutual respect fostered good faculty-student relationships, avoiding the creation of hierarchies in the personal sphere:

*P89*: *Well*, *I try to talk to them at their level*, *telling jokes like the ones kids their age tell*, *using some of the words they use*. *I want them to see me as approachable*, *to feel that the relationship between us is symmetrical*.

To foster this kind of close relationship, participants said they tried to connect with their students and to empathize with their tastes and preferences:

*P5*: *I try to use a bit of psychology*, *you know*, *like emotional intelligence*, *empathy* … *finding out about their individual tastes to try and make a connection*.

c) Faculty members know the names of the students

Another simple yet powerful strategy, which participants said they often had recourse to, was learning students' names, because this generated closeness and encouraged students to become more interested and engaged. Specifically, 17 faculty members described this strategy:

*P15*: *For me*, *it's really important to learn everyone's name*. *The simple fact of being able to call someone by their name really helps*. *It's a trivial thing really*, *but it helps*. *Just being able to call on them in class saying "María" instead of "you"*.

d) Use of spaces outside the classroom

Finally, a few faculty members said they often tried to find spaces and moments outside the classroom context to foster good relationships with their students. For example, they used tutorials or more informal settings, like the faculty cafeteria, to get to know their students better and establish closer relationships with them.

*P76*: *Well*, *just talking to them really*, *I try to talk to them*. *I hold tutorials*, *for example*, *about assignments and so forth*, *and I use that time to get to know them better*, *and help them get to know me better too*, *because I can be more open and friendly*.

## Conclusions and discussion

This article focuses on an area of the teaching and learning process that has hitherto received very little attention. Specifically, it focuses on how the affective-emotional component, and particularly personal connections and relationships between faculty and students, affect learning. Other studies have previously outlined the need for further research in this field [29, 24].

Before focusing on the main conclusions of this study, we would like to acknowledge that as in any other qualitative study, we do not intend to generalize the results of our research. We would like to show and share a reality, which is none other than that of the 119 faculty members who base their practices on education inclusive. In this sense, and as others have already studied, inclusive faculty members go beyond disability, and think of all their students when they teach [2].

The first conclusion that can be drawn from the results of the study is that, in general, the actions taken to motivate students with disabilities are no different from those taken to motivate the rest of the student body. Similarly, the strategies used to foster good faculty-student relationships are the same for both students with disabilities and other students. As Cunningham [39] and McKay and Devlin [40] stated previously, this suggests that positive practices are positive for everyone. Indeed, it is possible that the participants in our study could have been nominated by any student, since their actions are no different from those identified in previous research into what makes a good university teacher [41–43]. The novel contribution made by our study is that, for the first time, the focus is exclusively on what *inclusive* university faculty members do. These faculty members were not identified because of their best practices, or because they won a teaching award or participated in consecutive teaching innovation projects. Rather, exclusively their own students with disabilities identified them, and it is in this that the value of this study lies. The majority of studies carried out to date have focused on identifying, through student voices, the helps and hindrances encountered in relation to inclusion/exclusion [3, 7]; however, to date, no research has focused on faculty members' voices, seeking to understand how they contribute to the inclusion of students with disabilities.

From the results reported here it could be concluded that few faculty members do anything different to motivate students with disabilities, and when they do, their actions are linked to a way of teaching best defined in terms of its human component [31]. Basically, they show affection, evince an interest in their students and strive to give them confidence. All these actions reveal an idea of what it is to be a faculty member that is similar to the profile required by an inclusive university which welcomes diversity and is based on the principles of equality and social justice [2]. This faculty profile can serve as an inspiration for universities to design training policies that not only design courses on teaching methodologies, but also incorporate aspects related to emotions or motivation in the training actions.

Although the results of the study point to a number of strategies that may help motivate students, there is also a prior issue to bear in mind in relation to both faculty and students: the vocation felt by students in relation to their chosen area of study and the faculty member's self-motivation, preparation and training. Indeed, participants demonstrated a strong sense of vocation and passion for teaching. Ruíz-Alonso and León [34] defined the passion teachers have for their profession in terms of enthusiasm, passion for both the subject being taught and students, and a belief in both themselves and the fact that their teaching may make a difference to their students' lives. The faculty members participating in our study fit this description and demonstrate this passion to their students through their actions [30].

Furthermore, and in relation to motivation, another conclusion that can be drawn from the results is that students with disabilities are extremely motivated. This finding is consistent with that reported by Bye et al. [21], and indicates that faculty do not have to work any harder to engage this group of students in their subjects. Nevertheless, participants in our study did claim to be aware of and use specific strategies for making their subjects more attractive. One of these is linked to organizing the classroom to make it more inclusive. The aim is to create a good climate [19] and a welcoming atmosphere, and to foster a sense of belonging. Previous studies have found that this feeling is vital for avoiding student dropout [11, 13]. However, there are also academic aspects which the participants in our study believe help foster student motivation, including useful content for future professional activities, active and participatory methodologies, varied resources for adapting to different learning styles and ongoing feedback. This same finding was reported by Thomas [2] in a study on elements which contributed to student retention. However, there are also other aspects which influence motivation, including one which participants claimed was their principal strategy: the establishment of good faculty-student relationships through emotional connection. Again, universities should take note and learn from the faculty who teach based on inclusive education. What they do and how they do it can help transfer these practices to other faculty members and contagion them from their good work. Previous studies also concluded this [27,15]. Thus, university faculty should bear in mind that it is not only the content of their lessons that is important, but that they should also pay attention to *how* they teach, in a twofold sense, since while there can be no doubt that methodologies impact learning, it is no less certain that the climate generated and the way in which faculty and students interact are also vital elements in this process. Therefore, steps should be taken in university training programs to help prepare faculty to develop the triangle proposed by Mayer et al. [18]: cognition, motivation and emotion. It is also recommended that universities encourage faculty members to adopt the universal design for learning among their teaching practices [44], since this approach to teaching incorporates all students, including those with disabilities. It is planned taking into account the different forms of expression, representation and involvement in learning.

There are also other factors which have more to do with awareness than with training. Indeed, the faculty members who participated in this study are all people who maintain close relationships and engage in horizontal communication with their students. They show an interest in them and are empathetic. They open up to students and are concerned about getting to know them in turn, identifying their needs and finding ways to help them. In sum, they are committed to training processes, believe in the advantages of diversity and strive to act in accordance with this belief. By doing so, they show us that another kind of university is possible. Their actions demonstrate that faculty can help build high-quality universities that stand out not only for their research excellence, but also for their first-class teaching. In short, they can help build more human and socially-committed universities. The social dimension of higher education requires that universities play a key role in promoting social cohesion, reducing inequality, providing opportunities and helping improve the general quality of the societies in which they are embedded. From this social perspective, we are firmly convinced that universities can only attain high quality if they are also accessible and inclusive.

### Limitations and future research

This study has a number of limitations which should be taken into consideration, one being the time spent recruiting participants. The process was slow and lasted a whole year, mainly because we had to negotiate with the support services in the different universities in order to communicate the project to students with disabilities (who were responsible for nominating faculty members engaging in inclusive pedagogy). In order to access more participants, we were also obliged to contact different universities and other people not originally included in the initial project in order to expand the sample group.

Another limitation was the time spent gathering data, since faculty members were overburdened with research and teaching tasks and it was often difficult for them to find time to conduct two long interviews.

A third aspect that could be considered a limitation is that no separate analysis was conducted in accordance with either university or field of knowledge. Nevertheless, this was not the aim of the study, and nor were any significant data found which would enable a differential analysis on the basis of these criteria.

Despite these limitations, however, we believe that our study is novel and fills a gap in the research carried out to date on higher education and inclusive education. Future research should strive to further explore this field by carrying out classroom observations in order to investigate these practices and analyze in more detail the actions carried out by these faculty members. Moreover, interviews could be held with students with disabilities in order to determine the most effective strategies that contribute to their learning and engagement. Finally, it would also be useful to listen to the voices of the rest of the student body, in order to identify and explore in more detail the best practices engaged in by faculty members.

## Supporting information

S1 File Interview script (Guión de entrevista). (DOC)

S2 File Data analysis results (Resultados del Análisis de Datos). (DOCX)
