# Supplementary material for: The keys to learning for university students with disabilities: Motivation, emotion and faculty-student relationships
Source: PLoS One. 2019 May 22;14(5):e0215249. doi: 10.1371/journal.pone.0215249 (PMC6530886; doi:10.1371/journal.pone.0215249)
Supplement: S1 File — (DOC) [file pone.0215249.s001.doc]

**
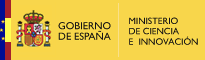
**
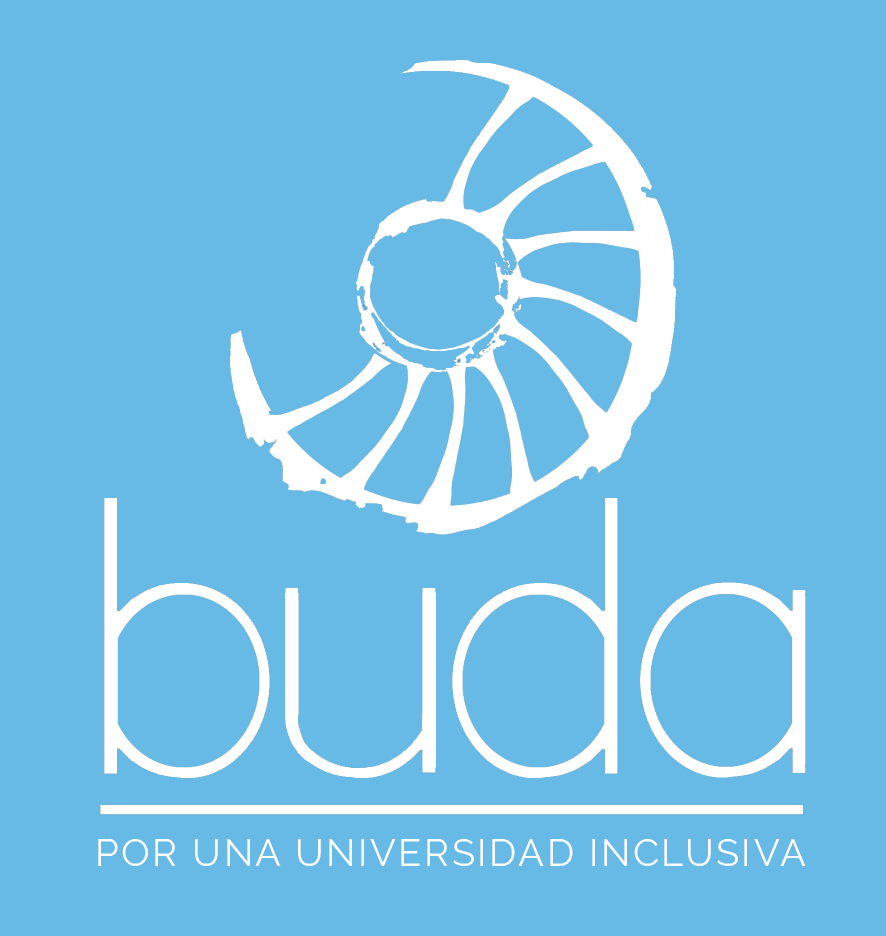
**
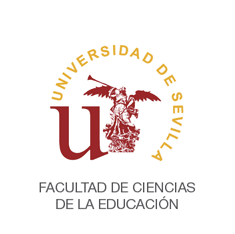
**

**GUIÓN ENTREVISTA. ACCIONES**

Proyecto: Pedagogía inclusiva en la universidad: narrativas del profesorado
(MINECO, ref. [EDU2016-76587-R](https://investigacion.us.es/sisius/sis_proyecto.php?idproy=27540))

**OBJETIVO:** Describir y explicar las acciones del profesorado universitario para desarrollar una pedagogía inclusiva en la universidad.

**Acciones:** estrategias afectivas, emocionales y de enseñanza y aprendizaje, puestas en práctica para el desarrollo de una pedagogía inclusiva.

**ACCIONES**

**PROCESO DE ENSEÑANZA Y APRENDIZAJE EN GENERAL**

- ¿Qué aspectos consideras que son los más importantes (organización y disposición del aula, buen funcionamiento de dispositivos electrónicos, buena iluminación y acústica en el aula, metodología, etc.) para desarrollar prácticas de educación inclusiva, es decir, para que pueda aprender y participar todo el alumnado?
- Nos gustaría ponernos en situación y que nos contaras cómo es una clase, desde que se inicia hasta que finaliza.
- ¿Qué haces para conocer cuáles son las necesidades o dificultades concretas que un estudiante tiene para seguir con éxito tu asignatura? ¿Qué haces para ayudarle a superarlas?
- Si le pudiéramos preguntar a un alumno qué mejoraría de tus clases, ¿qué crees que diría?
- Desde tu papel como docente: ¿cuáles consideras que son las principales dificultades a las que te enfrentas al ayudar a aprender al alumnado y en especial al alumnado con discapacidad?

**RELACIÓN PROFESORADO-ALUMNADO**

- ¿Para ti es importante la relación que se establece entre alumnado y profesorado? ¿Por qué?
- ¿Qué prácticas/ estrategias llevas a cabo para fomentar la relación con tu alumnado?
- ¿Cómo crees que la relación con los alumnos puede influir en el aprendizaje del alumnado?

**MOTIVACIÓN ESTUDIANTES**

- Desde tu punto de vista, ¿cuáles consideras que son piezas clave para conseguir un alumnado implicado y motivado durante todo el proceso de enseñanza-aprendizaje?
- ¿Cuál(es) piensas que son las causas más frecuentes de la desmotivación de tu alumnado? ¿por qué?
- ¿A qué recursos sueles acudir cuando ve que se está produciendo un descenso en la motivación e implicación de tu alumnado?
- En cuanto al alumnado con discapacidad, ¿haces cosas distintas para motivarlos?¿Por qué? En caso afirmativo, ¿cómo lo haces?

**Para finalizar esta entrevista, nos gustaría saber ¿cómo te ha influido, a nivel personal y profesional, haber tenido estudiantes con discapacidad en el aula?**

¿Deseas añadir alguna cuestión o información que no haya sido incluido en esta entrevista?
