# Supplementary material for: The keys to learning for university students with disabilities: Motivation, emotion and faculty-student relationships
Source: PLoS One. 2019 May 22;14(5):e0215249. doi: 10.1371/journal.pone.0215249 (PMC6530886; doi:10.1371/journal.pone.0215249)
Supplement: S2 File — (DOCX) [file pone.0215249.s002.docx]

**
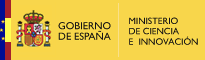
**
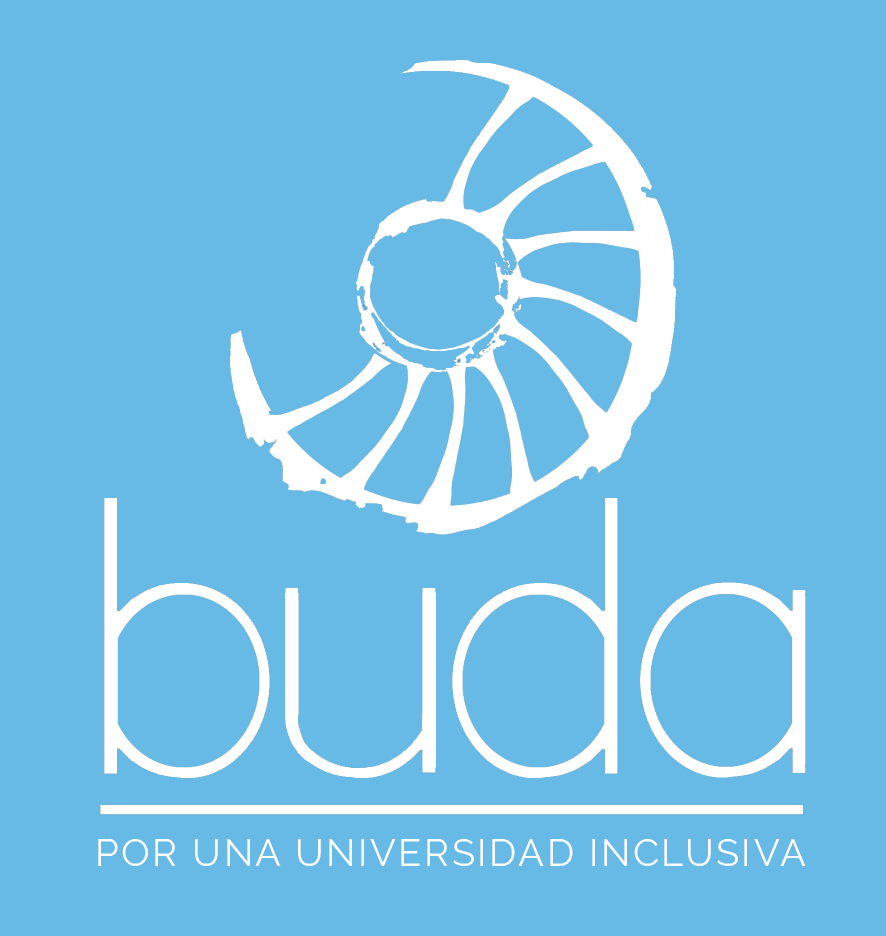
**
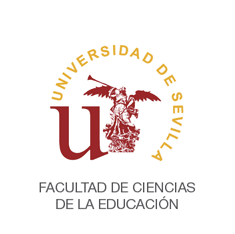
**

**RESULTADOS ANÁLISIS DE DATOS (Ejemplo. Código importancia relaciones)**

Proyecto: Pedagogía inclusiva en la universidad: narrativas del profesorado  (MINECO, ref. [EDU2016-76587-R](https://investigacion.us.es/sisius/sis_proyecto.php?idproy=27540))

**Documento: 1. Arte y Humanidades\P 1 Arte Diseños**

**Peso: 0**

**Posición: 31 - 33**

**Código: 4. Acciones\Relación profesorado-alumnado\4.15. Importancia relación**

P1: Bueno, pues yo tengo la costumbre de poner las tutorías coincidiendo con taller, de tal menara, que en cualquier momento que el alumno me necesite, pues me tenga a su disposición.

E: Entonces, les das consejos…

P1: Sí, yo les doy confianza a los técnicos. Ellos hacen su trabajo perfectamente y es una manera pues, de conocerlos más.

**Documento: 1. Arte y Humanidades\P 1 Arte Diseños**

**Peso: 0**

**Posición: 66 - 67**

**Código: 4. Acciones\Relación profesorado-alumnado\4.15. Importancia relación**

E: ¿Para ti es importante la relación que se establece entre alumnado y profesorado? ¿Por qué?

P1: Me parece fundamental ya que tenemos que convivir. Pienso que se ha perdido mucho la autoridad el profesor, pero aún así, yo creo bien que…el respeto hacia una persona no está en hablarle de usted, el respeto es otras cosas y lo serio no es siempre lo serio, sino que lo serio está en hacer las cosas correctamente. Es necesario, somos seres humanos y manifestar las cosas…porque tú utilices el humor no significa que estés faltando el respeto, sino quizás estás respetando más a los demás. Yo creo que con respeto todo se puede hacer, y las relaciones son muy importantes. Esa relación donde tú eres el profesor y ellos los alumnos y que tú eres el referente y si no lo eres, pues algo falla. Y no eres el referente porque seas el mejor del mundo mundial, sino porque ellos piden de ti ciertas cosas.

**Documento: 1. Arte y Humanidades\P 1 Arte Diseños**

**Peso: 0**

**Posición: 68 - 69**

**Código: 4. Acciones\Relación profesorado-alumnado\4.15. Importancia relación**

E: Y, ¿qué tipo de prácticas o estrategias llevas a cabo para fomentar la relación con tu alumnado?

P1: Bueno, pues les hablo como hablan entre ellos, me pongo a su nivel, yo no me la voy a dar de guay y tengo que estar al día de las cosas que rodean a su realidad y se aprende mucho. Un profesor tiene que saber entrarles a sus alumnos, y una vez que esto sucede, pues ellos te llevan a su terreno y aprendes un montón.

**Documento: 1. Arte y Humanidades\P 2 Arte Diseños**

**Peso: 0**

**Posición: 58 - 61**

**Código: 4. Acciones\Relación profesorado-alumnado\4.15. Importancia relación**

E: Ya, depende de… ¿Para ti es importante la relación que se establece entre el alumnado y el profesorado? Y por qué.

P2: Creo que es fundamental. Creo que, que el alumno vea, independientemente del profesor, el conocimiento, etc., hay una persona, es fundamental ¿Por qué? Porque creo que estamos en una sociedad en la que la información y el conocimiento no tienen nada que ver con la información y el conocimiento de hace unos años. Hace treinta o cuarenta años o quizá más atrás, el profesor titular o el catedrático era el exponente máximo de ese conocimiento. Ese conocimiento ahora lo podemos encontrar en muchos otros sitios, lo que sí que tiene que tener claro el alumno es que la persona que está ahí como profesor, no solamente conoce, que puede conocer más o menos como él incluso, pero sí te puede dar una serie de instrumentos y herramientas y una metodología de trabajo en la que puedes progresar. Yo creo que ahí es donde está el verdadero descubrimiento, ¿de acuerdo? Yo ahora mismo no tengo necesidad de conocerme los Reyes Godos, aunque estuviese en historia del arte, ni tengo por qué decirte de memoria cuáles son los pintores franceses de pe a pa, ¿por qué? Porque tengo una herramienta a mano, tan a mano, que puedo dedicarme a otro tipo de cuestiones. Pero lo que sí que debe saber es qué metodología debe utilizar el alumno para progresar, eso sí lo puedo saber yo.

E: Qué prácticas o estrategias llevas a cabo para favorecer la relación con tu alumnado.

P2: Pues mira, el tipo de ejercicio, puestas en común, ejercicios colaborativos, etc. Y, después una cosa que me funciona desde hace treinta y tres años que es un café a las nueve de la mañana. Ese café comunitario en el aula, poniendo cada uno un poco de café, té y unas magdalenas, es fundamental. Ahí, la mayoría de las tutorías desaparecen.

**Documento: 1. Arte y Humanidades\P 3 Arte Diseños**

**Peso: 0**

**Posición: 52 - 53**

**Código: 4. Acciones\Relación profesorado-alumnado\4.15. Importancia relación**

E: En relación a lo que tiene que ver el profesor y el alumnado. Para ti es importante la relación que se establece entre alumnado y profesorado, por qué.

P3: Me parece importante, pero también imposible, al menos en este curso, para mí es imposible porque tengo 160 alumnos y yo no soy capaz ni de recordar los nombres, me quedo con las caras, pero no sé si es de este curso o de otro. Me quedo con las caras de los que siempre vienen. Quizás, ahora me he aprendido cinco nombres, es que soy fatal para los nombres. Pero, no puedo establecer un contacto. Antes, cuando daba clase en segundo año para los restauradores en ciencia aplicada tenía grupos más pequeños entre 15 y 20 alumnos, entonces ahí sí se puede establecer este vínculo. Y es algo muy importante porque si se establece ese contacto, primero confían más en ti y luego, tú te das cuenta de sus necesidades y les puedes ayudar mucho más y éste es nuestro principal papel.

**Documento: 1. Arte y Humanidades\P 3 Arte Diseños**

**Peso: 0**

**Posición: 54 - 55**

**Código: 4. Acciones\Relación profesorado-alumnado\4.15. Importancia relación**

E: ¿Qué prácticas o estrategias llevas a cabo para fomentar la relación con tu alumnado?

P3: Ninguna. No sé, si me los encuentro por el pasillo, pues les hablo. Además, siempre les saludo, me parece muy importante saludar y el desear un buen fin de semana. No sé un acercamiento más personalizado, siempre les digo que me pueden buscar para cualquier cosa y que me pueden escribir emails, pero más de esto no hago. Además, me gustaría, pero tampoco sé cómo hacerlo, no sé cómo acercarme a un grupo de 40 alumnos, seguro que hay herramientas, pero yo no sé y no las conozco. Entonces, me quedo con las caras que están siempre.

**Documento: 1. Arte y Humanidades\P 4 Arte Creencias**

**Peso: 0**

**Posición: 37 - 37**

**Código: 4. Acciones\Relación profesorado-alumnado\4.15. Importancia relación**

yo ya te digo, llevo 24 años, y te conviertes un poco en psicólogo y no porque lo pretendas, sino porque tú te das cuenta y dices bueno, igual que hay expertos caligráficos, la caligrafía no es ni más ni menos que un dibujo en el que se refleja el estado de ánimo.

**Documento: 1. Arte y Humanidades\P 4 Arte Creencias**

**Peso: 0**

**Posición: 37 - 37**

**Código: 4. Acciones\Relación profesorado-alumnado\4.15. Importancia relación**

Yo veo dibujos de mis alumnos y me voy para ellos y les digo “qué te pasa hoy”, porque hay una temperatura, una sensibilidad en la línea, que transmite un estado de ánimo

**Documento: 1. Arte y Humanidades\P 4 Arte Diseños**

**Peso: 0**

**Posición: 58 - 61**

**Código: 4. Acciones\Relación profesorado-alumnado\4.15. Importancia relación**

E: Vale. Y en cuanto a la relación profesor-alumno, me imagino que para ti es importante esa relación por lo que me has ido comentando.

P4: Sí, claro.

E: Y qué prácticas llevas a cabo para favorecer la relación con los estudiantes

P4: Verás, hay dos parámetros que yo creo que no deben faltar, uno es el respeto absoluto, no ya te digo profesor-alumno, sino a las personas, no distingo profesor alumno en ese sentido, sino el respeto comunitario. Y, eso, la universidad funciona. Desde el primer día les digo las normas de la clase, en mis clases les ruego que por favor estén en silencio porque es un proceso de concentración, un desgaste psicológico y físico y con el agravante de que tenemos unas personas que son unos modelos, unos profesionales que están ahí y que necesitan respeto. Yo no soporto que alguien esté hablando y el modelo esté posando para ti, eso hay que respetarlo. Entonces, en mis clases, silencio absoluto. Esa dinámica se lleva muy bien porque como hay periodos de pose y periodos de descanso. Bueno, pues en el descanso charlan, se toman un café y vuelven. Somos mayores de edad, el que tenga una necesidad, pues se sale de la clase y cuando se interrumpa en otro descanso, se incorpora. Yo creo que el respeto es fundamental. Y, por mi parte, yo intento ser lo más natural posible, siempre desde el respeto. Lo más cercano, estoy a su disposición e intento hacerle fácil lo difícil que ya es dibujar.

**Documento: 1. Arte y Humanidades\P 5 Arte Diseños**

**Peso: 0**

**Posición: 60 - 65**

**Código: 4. Acciones\Relación profesorado-alumnado\4.15. Importancia relación**

E: En relación a lo que sería la relación profesor-alumno. ¿Para ti es importante la relación que se establece entre el alumnado y el profesorado?

P5: Sí.

E: ¿Por qué?

P5: Porque como te dije anteriormente, esto es una cuestión de comunicación continua y de interrelación entre personas. Entonces, si no existe esa unión, ese vínculo…es que cuando nos paramos a recordar a los profesores que hemos tenido en nuestra escolarización, siempre pensamos en aquellos con los que hemos conectados. Ahora, esta conexión no siempre la consigo con todos los estudiantes, pero sí lo he intentado.

E: ¿Qué prácticas o estrategias llevas a cabo para fomentar la relación con tu alumnado?

P5: Yo intento ir muy directo, no intento crear barrearas entre el estudiante y yo, pero eso no significa que vaya de colega del estudiante, pero no me gusta crear una barrera en plan de yo soy el profesor aquí y tú solo eres un alumno más. Yo intento fomentar el tema de tutoría, de trabajo en quipo, de reuniones, etc., no sé un poco de sentido común. También, intento utilizar la psicología un poco, en el sentido de la inteligencia emocional, de la empatía, de ver cuáles son los gustos de esa persona, de acercarme y tal. Pero bueno, intentar empatizar con estas cuestiones, pues considero que es un punto clave.

**Documento: 1. Arte y Humanidades\P 6 Arte Diseños**

**Peso: 0**

**Posición: 58 - 59**

**Código: 4. Acciones\Relación profesorado-alumnado\4.15. Importancia relación**

E: En relación a lo que sería la relación entre el profesorado y alumno. Para ti, es importante la relación que se establece entre el alumnado y el profesorado y por qué.

P6: Por supuesto que es muy importante por lo que estaba comentando al principio. Es decir, si yo no sé la problemática de ese alumno, yo no sé las necesidades que tiene ese alumno, pues yo no le puedo dar una solución. Digo que para mí es importante esa primera parte, ya que si el alumno viene con una información previa que yo debería saber, pues me gustaría saberla antes.

**Documento: 1. Arte y Humanidades\P 6 Arte Diseños**

**Peso: 0**

**Posición: 60 - 61**

**Código: 4. Acciones\Relación profesorado-alumnado\4.15. Importancia relación**

E: ¿Qué prácticas o estrategias llevas a cabo para fomentar la relación con tu alumnado?

P6: Pues, hablar muchísimo, yo hablo diariamente con todos mis alumnos y varias veces si puedo.

**Documento: 1. Arte y Humanidades\P 7 Arte Diseños**

**Peso: 0**

**Posición: 50 - 53**

**Código: 4. Acciones\Relación profesorado-alumnado\4.15. Importancia relación**

E: ¿Para ti es importante la relación que se establece entre profesor-alumnado y por qué?

P7: Hombre es básico. Yo freo que la confianza es fundamental, ya que, si ellos no tienen confianza en mí, pues me van a ver como un ogro. Es más, las asignaturas que yo imparto son bastante ogro, duras y sin embargo, tengo un índice de superación que se lo tiene ganado, no es que yo les pase la mano, es que hacen un trabajo magnífico. Y, además, esto lo he visto luego en los TFG porque han sacado matrícula en los TFG.

E: ¿Qué prácticas o estrategias llevas a cabo para fomentar la relación con tu alumnado?

P7: Pues, intentar acercarme a ellos, aunque me cuesta mucho porque, aunque no lo parezca soy tímida. Pero eso, intentar acercarme hablando con ellos y mostrándome muy disponible. Y cuando ellos ven que te mandan un email ya sea sábado o domingo y yo le contesto, pues eso dice mucho. Y después, llego a clase y digo, “oye que me habéis mandado un email un sábado” y se ríen y ellos me dice, “pero has contestado”. Entonces, yo creo que, en la medida de lo posible, pues hay que estar disponible.

**Documento: 1. Arte y Humanidades\P 8 Arte Diseños**

**Peso: 0**

**Posición: 100 - 103**

**Código: 4. Acciones\Relación profesorado-alumnado\4.15. Importancia relación**

E: ¿Para ti es importante la relación que se establece entre alumnado y profesorado? Y, ¿por qué?

P8: Hombre claro, es que es super importante, claro que sí. Es como una pequeña familia con un objetivo común, el que alcancen los objetivos y que durante el camino se sientan a gusto.

E: ¿Qué prácticas o estrategias llevas a cabo para fomentar la relación con tu alumnado?

P8: Qué prácticas…pues trato de hacer ejercicios y de hacer propuestas que les susciten mucho interés, que les despierten la imaginación y la creatividad.

**Documento: 1. Arte y Humanidades\P 9 Arte Diseños**

**Peso: 0**

**Posición: 88 - 91**

**Código: 4. Acciones\Relación profesorado-alumnado\4.15. Importancia relación**

E: Hablando como antes nos has dicho sobre la confianza que hay que tener con el alumnado, siempre manteniendo cierta distancia, porque tampoco una relación tiene que ser estrecha, manteniendo un respeto. ¿Para ti es importante la relación que se establece entre el alumnado y el profesorado?

P9: Claro, totalmente. Si tu no pones expectativas en esos alumnos, esos alumnos se toman la asignatura de otra manera. Si tú no confías en ellos o ellos ven ilusión por lo que estás enseñando, o sea, si ellos ven que ni tú misma te lo crees, es que no transmites nada y eso se nota. Eso es como el lenguaje subjetivo, ¿no?, que se nota, yo creo que se nota desde un principio. En comentarios que haces, en, no sé, en esas cosas que parece que no se ven pero que están ahí, y que juegan un papel muy importante. En la asignatura de Video-creación, hay un ejemplo del inicio del cine, de los Hermanos Lumière, que ellos no habían inventado ni si quiera el primer plano. Ellos grababan desde un punto fijo como si fuera un teatro, entonces la escena, todo ocurría en ese espacio, como si fuera en ese teatro ¿no?, entonces las primeras películas eran así, eran como un teatro, imagen fija y todo lo que ocurría, ocurría ahí. No se movía, ni izquierda y derecha, ni arriba y abajo. Entonces, les pongo el ejemplo de una película que hicieron que estaban representando la crucifixión de Cristo y ahora, de repente la gente en el cine “*jajajaja*” venga a reírse, venga a reírse. Y qué pasa ¿no? Y es un ejemplo que yo les pongo para que ellos tengan en cuenta que, cada vez que van a grabar, lo que aparece en cámara, ¿no? Y son esos detalles que estamos hablando ahora que le dan connotación a lo que sucede en aula y resulta que, en esa escena de crucifixión, que era lo más cruel y lo más duro, hombre, estaba Jesucristo con las dos cruces y él padeciendo y tal, y la gente riéndose en el cine. Y qué pasaba, que resulta que cuando habían grabado esa imagen, se les había cruzado una gallina con los polluelos y había una mujer detrás corriendo con los polluelos. Qué pasó, que eso es como la historia de Ice Age, de la ardillita, es como una historia paralela a lo que está pasando en el cine. Pues esto igual, esa gallinita que puede pasar, que de repente se ha colado y que ha captado la atención y que todo el mundo se fija en ella, y no están en lo que tienen que estar realmente, pues sucede en las clases.

E: ¿Y qué practicas o estrategias llevas a cabo para fomentar esa relación con tu alumnado?

P9: Pues hablar con ellos, consensuar, dialogar. No es solamente hablar, que yo transmita, no. Ellos también tienen que intervenir.

**Documento: 1. Arte y Humanidades\P 10 Arte Diseños**

**Peso: 0**

**Posición: 62 - 67**

**Código: 4. Acciones\Relación profesorado-alumnado\4.15. Importancia relación**

E: ¿Para ti es importante la relación que se establece entre alumnado y profesorado? Y, por qué.

P10: Sí, es importante en el sentido de que, al fin y al cabo, pasan muchas horas juntos en las clases, los alumnos suelen ver a los profesores como con cierto referente o ejemplo, porque como te ven con más experiencia o conocimiento, sienten que pueden aprender. Entonces, es una relación fuerte. En mi caso, también, al ser joven y eso, soy muy sociable, me suelo llevar bien con ellos, suelo tener una actitud anímica muy alegre con ellos… Y eso hace que ellos también se sientan más cercanos, en este caso. Y más o menos yo diría que es eso, pero yo también intento guardar la distancia y que no piensen o no se sobrepasen de que puedan pasarse en confianza, que no se extralimiten en confianza. Entonces, no me caso tampoco con ellos y ellas.

E: Qué prácticas o estrategias sueles llevar a cabo para favorecer esta relación con tu alumnado.

P10: Bueno, antes me preguntaste también por qué en esa pregunta. Yo diría que porque afianza los lazos y genera motivación en ellos.

E: Y ahora es qué prácticas o estrategias sueles llevar a cabo para favorecer esta relación con tu alumnado.

P10: Sí, que me muestro cercano, empático, que les pregunto cómo va todo, cómo va la cosa… Preguntas tipo así, “¿la semana pasada bien?, cómo ha ido” y en un momento dado, a lo mejor hago preguntas de “¿y el curso? ¿Os sentís bien? Cómo va”, pregunto cosas así.

**Documento: 1. Arte y Humanidades\P 11 Arte Diseños**

**Peso: 0**

**Posición: 38 - 39**

**Código: 4. Acciones\Relación profesorado-alumnado\4.15. Importancia relación**

E: Consideras que a la hora de dar clases, ¿es importante la relación que tienes con tus alumnos para su aprendizaje?

P11: Hombre, yo creo que eso siempre es importante. Pero, siempre y cuando no se pase el umbral. Evidentemente, al ser una asignatura donde hay 5 o 7 equipos en cada clase, pues no son muchos, entonces, se pueden ir viendo las cosas en la misma clase. Entonces, al ser pocos alumnos, pues te permite conocerlos y tener un trato más cercano. Y al ser una clase muy práctica, hay mucho diálogo, presentaciones, ellos traen problemas y en clase se intentan resolver. Aunque luego, es muy gracioso porque lo explicas todo y llegan equipos con las cosas totalmente distintas a como las has explicado tú. Así que, imagínate cuando hacemos las preguntas en quipo relacionados con las películas, pues hubo un grupo, que la hicieron sobre el “Señor de los Anillos”, y eran preguntas en plan, “¿dónde se rodó el Señor de los Anillos?” y preguntas así, pues entontes, les puse un cero porque no se habían enterado de nada. Y eso que lo habíamos hablado ochenta veces en clase.

**Documento: 1. Arte y Humanidades\P 12 Arte Diseños**

**Peso: 0**

**Posición: 21 - 21**

**Código: 4. Acciones\Relación profesorado-alumnado\4.15. Importancia relación**

P12: No, no, porque ya te digo, en el momento en que se me informa o cuando el mismo chico o chica me dice que tiene…pues entonces hablo con ellos y me dicen qué necesitan, o “¿tú puedes hacer esto?, tal, ¿tú tienes dificultades para escribir?”, vamos a ver, para un examen se puede tomar todo el tiempo que quiera, pero igual que todo el mundo o sea que no me lo planteo porque he tenido, como te comentaba el otro día, cinco o seis alumnos con discapacidad a lo largo de mi vida, cada uno muy diferente y de algunos me he hecho muy amiga, por esta cuestión de que has conectado, por entusiasmo vital.

**Documento: 1. Arte y Humanidades\P 12 Arte Diseños**

**Peso: 0**

**Posición: 36 - 39**

**Código: 4. Acciones\Relación profesorado-alumnado\4.15. Importancia relación**

E: Luego, con respecto a la relación entre el profesor y el alumno, tú ya me has mencionado el otro día y hoy también, que es muy importante esa relación. ¿Por qué crees que es tan importante y qué cosas haces para fomentar la relación con tu alumnado?

P12: Bueno, insistirles mucho en que vengan a tutorías, aunque afortunadamente tengo grupos pequeños ahora, antes no lo podía hacer, ¿eh? Para ver sus necesidades les dices “tú tienes tal trabajo, pero el tema lo tienes que consensuar conmigo”, y entonces, obligatoriamente vienen al despacho a consensuar los temas y tú ya lo conoces enseguida, pero claro, esto lo puedes hacer este año con una clase que yo tengo este año, pero yo comprendo perfectamente que con una clase de 100 alumnos esto no se puede hacer. Yo cuando he tenido otras clases hace unos años, no lo podía hacer, o sea, que la atención también va dependiendo, sobre todo, de los estudiantes que tengas.

E: Claro, claro. Y tú cómo valoras esta relación con los estudiantes.

P12: Muy buena.

**Documento: 1. Arte y Humanidades\P 12 Arte Diseños**

**Peso: 0**

**Posición: 40 - 41**

**Código: 4. Acciones\Relación profesorado-alumnado\4.15. Importancia relación**

E: Y tú cómo crees que eso puede influir en su aprendizaje.

P12: Hombre, desde el momento que se facilita un sistema de exámenes y tal, yo creo que sin perder…jolín, es que yo tengo muchos más años que ella, no es lo mismo que cuando empecé que tenía su edad, pero que antes, a lo mejor nos íbamos hasta de fiesta, ahora ya no, pero yo me acuerdo que a mí me gustaba mucho que me escucharan. Yo me acuerdo de cuando yo estudiaba. Yo creo que acordarse de cuando uno fue alumno, es parte esencial de ser profe.

**Documento: 1. Arte y Humanidades\P 13 Arte Diseños**

**Peso: 0**

**Posición: 68 - 71**

**Código: 4. Acciones\Relación profesorado-alumnado\4.15. Importancia relación**

E: Vale. Y bueno, yo creo que esta ya está respondida, pero, ¿para ti es importante la relación que se establece entre el alumnado y el profesorado? Hemos dicho que sí, que la comunicación la consideras esencial, pero ¿alguna otra noción que quisieras dar de por qué consideras importante esa relación?

P. 13: Pues, bueno, porque el ambiente de clase sea distendido, me gusta entrar en clase, digamos, y encontrarme a mis alumnos con una sonrisa en la cara, y no “uf, ahora toca inglés, aquí está esta”, ¿sabes? A mí me gusta entrar en clase y ver a mis alumnos contentos, si ellos están contentos, yo estoy contenta. Cuando algo no funciona, yo lo llevo peor, no sé, a mí me gusta estar con mis alumnos en clase, no concibo la docencia de otra forma.

E: Claro. Y, de las prácticas y estrategias que llevas a cabo para fomentarla, hemos dicho que preguntarles, preocuparte por ellos, cómo ha ido el fin de semana o las vacaciones… ¿Pero alguna otra estrategia que consideres remarcar o práctica?

P. 13: Bueno, pues sí, a veces sí, noto que se me da el caso, el año pasado, una alumna que estaba en primera fila y siempre estaba con cara de asustada porque le costaba mucho y tal, pues entonces, yo intento ser sensible a esa dificultad, ¿no? Y hablar con ella después de clase, poquito a poco, “lo estás haciendo bien” ... Eso mismo, ¿no? Captar un poco el lenguaje corporal de los alumnos.

**Documento: 1. Arte y Humanidades\P 14 Arte Diseños**

**Peso: 0**

**Posición: 39 - 39**

**Código: 4. Acciones\Relación profesorado-alumnado\4.15. Importancia relación**

Pero sí creo que en toda educación hay algo fundamental que es entrar e intentar que al mes y medio te sepas los nombres de la gente y ya está. Y si no te sabes los nombres de la gente no pasa nada, pero pregúntaselos, que ellos sepan que quieres saber de lo que opina Rosa, de lo que opina Alejandro… No quieres saber lo que opinas tú, “oye, tú”, eso hay que evitarlo. Pero, cuando queramos saber lo que opina alguien, preguntémosle a él, no lo digo por esta pregunta, sino, cuando hablamos de discapacidad, no hables de un sordo, háblale a este alumno. Entonces, esto a veces es como la madre, los niños cuando se ponen la rebeca, cuando la madre tiene frío o el padre piensa que ya ha llegado el momento, entonces, eso hay que evitarlo.

**Documento: 1. Arte y Humanidades\P 14 Arte Diseños**

**Peso: 0**

**Posición: 80 - 83**

**Código: 4. Acciones\Relación profesorado-alumnado\4.15. Importancia relación**

E: Hablamos ahora de la relación entre el profesor y el alumno, en este caso, de tu relación con los estudiantes. Para ti, ¿es importante la relación que se establece entre el alumno y el profesor?

P. 14: Sí, dentro del contexto de que yo soy el profesor y él es el alumno, es decir, no me interesa caer bien, pero me interesa que se sienta respetado en clase, cómodo dentro del sentido cómodo que pueda proporcionar una universidad, del contexto educativo, pero no necesito caer bien a un alumno. Sí que para mí es imprescindible que ese alumno siempre se sienta respetado, que pueda decir “me parece un impresentable, pero no por cómo me trata a mí, porque el trato es exquisito. No me gusta el Power Point que hace, no me gusta cómo enseña, pero a mí me trata bien”.

E: Y, ¿cómo consigues tú esa relación?

P. 14: Respetando a la gente ¿Tú qué quieres?, ¿que te respeten? Bueno, pues respeta, respetando… Y, sobre todo, estableciendo una jerarquía inexistente a nivel personal. Estamos trabajando en el mismo aula, cada uno con un rol, pero en el mismo…ninguno está por encima del otro. Es verdad que yo tengo unas obligaciones que ellos no tienen.

**Documento: 1. Arte y Humanidades\P 14 Arte Diseños**

**Peso: 0**

**Posición: 108 - 109**

**Código: 4. Acciones\Relación profesorado-alumnado\4.15. Importancia relación**

E: Hablando ahora de las tutorías, ¿vale? ¿Cómo organizas tú tus tutorías?

P. 14: Bueno, yo entiendo que las tutorías deben de ser un espacio donde el alumno venga independientemente de que sea examen. Entonces, intento…y este año voy a hacer…hay un libro que era “Lo que hacen los mejores profesores universitarios”, y ahí había un profesor que decía “comía con sus alumnos”, con todo, quiero decir, por grupos, ¿no? Y, no vamos a hacerlo claro, porque no tiene uno que ir a esas comidas ni obligar a nadie a que vaya, porque, además, pienso que tiene que ser una relación profesional, yo no tengo que ir al…yo no tengo que cenar con nadie, un buen médico no me escupe, me respeta y ya está. Entonces, las reseñas, todo el mundo va a venir aquí a revisar las reseñas, primero, porque como había muchos fallos, había veces que suspendía gente y le decía…bueno, suspender, era solo un punto y medio, la gente podía sacar un 0,5, y le decía “si usted hubiera venido a revisión, usted tendría ahora un 1,3 en la reseña”. Entonces, los voy a obligar, porque, además, algunos son de segundo y es una manera de naturalizar las tutorías, porque he visto que mucha gente que era muy seria conmigo, como muy retraídos, cuando ha acabado la clase, el curso, después era mucho más expresivo, entonces, pensé que para ellos había un profesor y después una persona y, solamente cuando acabó la asignatura fueron capaces de superar esa idea, ¿no? Entonces, esa es una manera, vienes a tutorías y así lo desmitificas.

**Documento: 1. Arte y Humanidades\P 15 Arte Diseños**

**Peso: 0**

**Posición: 44 - 45**

**Código: 4. Acciones\Relación profesorado-alumnado\4.15. Importancia relación**

E: Y, ¿para ti es importante la relación que se establece entre profesorado-alumnado?

P. 15: Sí, es importante. Ahí todos los profesores tenemos un planteamiento y es ese trato de camaradería que hay algunos profesores que hacen y otros, pues digamos, que mantener siempre una cierta distancia. Yo siempre mantengo una cierta distancia que a lo largo del desarrollo del cuatrimestre, esa distancia se va acortando, es decir, que poco a poco hay posibilidad de entrar ya en otras dinámicas, pero a mí en principio me gusta y creo que a ellos también, que te vean como referente de la asignatura, como jerárquicamente en otro nivel y que esa relación pueda llevar…pues bueno, hay ocasiones en las que puede llevar, no te voy a decir amistad, pero sí a una relación cercana, sí.

**Documento: 1. Arte y Humanidades\P 15 Arte Diseños**

**Peso: 0**

**Posición: 46 - 47**

**Código: 4. Acciones\Relación profesorado-alumnado\4.15. Importancia relación**

E: Y, ¿cuáles son las prácticas que tú haces para llegar a esa cercanía?

P. 15: Pues, para mí, algo muy importante y es que desde muy pronto me gusta aprenderme los nombres de todos. Entonces, el hecho de llamar a cada uno por su nombre, desde muy pronto ayuda muchísimo, es una tontería, pero ayuda, en lugar de decir “tú”, decir “María”. Luego, el pasar de una locución muy estándar, muy neutra, a una locución más cálida en la que quepa la ironía o incluso unas formas de cortesía verbal que aproximen a las personas, el trato personal, por ejemplo, en las prácticas, el estar pendiente y ver lo que están haciendo, acercarte, incluso con bromas… Es decir, son una serie de estrategias muy sencillas, pero que me ayudan a dar ese paso.

**Documento: 1. Arte y Humanidades\P 16 Arte Diseños**

**Peso: 0**

**Posición: 48 - 51**

**Código: 4. Acciones\Relación profesorado-alumnado\4.15. Importancia relación**

E: Otras de las cuestiones es la relación profesor-alumno. Me gustaría preguntarte que para ti, hasta qué punto es importante esa relación.

P. 16: Cuando yo entré aquí, entré a trabajar con 24 o 25 años, hay muy poca diferencia de edad con tus alumnos, entonces hay una relación muy buena porque eres una persona más o menos de su edad. Ahora, yo lo que quiero es que el trato sea lo más cordial posible, sabiendo que tú tienes una responsabilidad y que ellos tienen otra que es distinta a la tuya. Hace mucho tiempo, un compañero mío, el cual está jubilado ya, me dijo que yo tenía un problema porque yo tenía muy buena relación con mis alumnos y los trataba como si fueran amigos míos y que a la larga me iba a dar más problemas que beneficios. A lo que yo respondí, que yo no soy amigo de los alumnos, sino que yo tengo una relación cordial con ellos, ya que, si tengo buena relación, me permite hacer muchas cosas con ellos en el aula y sacarle mucho partido a la clase. Si tienes mala relación y solo le dices al alumno lo que hace mal, pues a ese alumno lo hundes. Por lo tanto, no es una relación de amistad, sino de cordialidad porque cuando tú estés a gusto vas a dar lo máximo de ti; si no estás a gusto, estás perdido.

E: Y, qué tipo de estrategias usas para fomentar la relación con el alumnado.

P. 16: Intento ser lo más cercano posible, pero hay una cosa que por definición has de ser cercano, mis asignaturas son de presentaciones y estas presentaciones se graban en vídeo. Cuando mis alumnos las hacen, yo les digo esto me gusta más, o esto me gusta menos, o esto yo lo pondría así, y ahora tú cuando estés en tu casa, puedes estar de acuerdo o no conmigo, pero esto sería entre el alumno y yo. Y en mi clase, esto no es así, en mi clase se hace en la clase, y el feedback se da en la clase, y el feedback individual se da en mi despacho, siempre de dos en dos, y vemos los vídeos y comentamos las cosas que están bien y las que pienso que pueden mejorar. Todo esto es individualizado. El primer día de clase, la primera sesión que tienen conmigo, es la venta de un esclavo. Los pongo en grupos, y yo elijo los grupos, y les doy 15 minutos para que vayan a cualquier sitio de la facultad, con una libreta, y consigan toda la información que puedan del compañero, porque yo voy a comprar un esclavo y necesito saber toda la información posible para comprar al mejor. Pero, sacarme sobre todo lo positivo y si sacáis lo negativo a mí no decídmelo. Esto lo hago todos los años, el primer día de clase. Después, cuando lo exponen, observo los errores que cometen, como por ejemplo, si no alzan la voz lo suficiente, si le dan la espalda a sus compañeros, etc. Entonces, luego, les dejo un listado para que vengan a tutorías, tienen que venir de dos en dos y cada uno cuenta con 10 minutos. Entonces, vemos el vídeo juntos y hacemos el feedback entre los tres. Esto, se hace durante todo el curso con las tres presentaciones. ¿Qué supone esto? Pues muchas horas, ¿cuál es el beneficio?, que si pongo la presentación del primer día cuando me venden el esclavo y pongo la última presentación, cuando me venden un producto, se puede comparar y han avanzado muchísimo porque a cada uno le dices específicamente qué está mal y cómo puede mejorar.

**Documento: 1. Arte y Humanidades\P 17 Arte Diseños**

**Peso: 0**

**Posición: 74 - 79**

**Código: 4. Acciones\Relación profesorado-alumnado\4.15. Importancia relación**

E: Bueno, vamos a pasar a hablar de la relación entre el profesorado y el alumnado, ¿para ti es importante esta relación?

P. 17: Sí.

E: Por qué.

P. 17: Por lo que te decía antes, porque creo que una buena relación favorece el aprendizaje y porque es la forma en cuánto más… cuánto más directa, más fácil es conocer las necesidades individuales, ¿no?

E: Claro. Y, ¿llevas a cabo alguna estrategia o práctica para favorecer esta relación? Me has hablado de que sueles estar cercana, que les preguntas directamente…

P. 17: Bueno, intento contestar los emails en menos de 24 horas, creo que eso influye, intento, darles tutorías personales para hablar, intento en clase mostrarme cercana… Bueno, muy abierta a que me planteen todas sus casuísticas, problemas e historias, que muchas veces son muchas y de todo tipo, pero suelo estar abierta, aunque, ya te digo, que algunas veces puede jugar en contra porque…

**Documento: 1. Arte y Humanidades\P 18 Arte Creencias**

**Peso: 0**

**Posición: 35 - 35**

**Código: 4. Acciones\Relación profesorado-alumnado\4.15. Importancia relación**

Intento ser muy cercano, no su amigo. Yo siempre les digo desde el principio que yo no soy su amigo, que sus amigos los escogen ellos y son con los que se van a tomar cervezas y a quienes les tienen que llorar si el profesor le suspende. Tampoco soy su padre, es decir, ellos tienen una serie de problemas personales que quienes los tienen que solucionar son su familia y su entorno, y si son problemas de otro tipo pues tendrán que buscar otro tipo de asesoramiento, pero nada. Yo con todo lo que tenga que ver con su formación como universitarios y como personas dentro de la universidad, yo estoy dispuesto a atenderles siempre, ¿no? Pero no soy ni su amigo ni su padre, yo soy su profesor e intentaré ser, como profesor, lo más cercano posible. Favorezco el tuteo, no porque eso me permita ser más cercano, considero que utilizando el voseo también se podría ser cercano, sino que estamos en una sociedad en el que el ochenta por ciento de la gente se tutea y bueno, pues ya está. Si me tratan de usted no pasa nada, pero sí que también les insisto en que un profesor es una persona que necesita y tiene derecho a exigir un respeto. Tiene derecho a que se planteen que es una persona que tiene una formación, una capacidad y una preparación de la que ellos deben hacer el máximo uso, pero que no es su colega, yo siempre digo “a mí me podéis llamar Doctor Miranda, profesor Miranda, P. 18, tratarme de tú o tratarme de usted, pero yo no soy eh tú, ese no soy yo”. Y un correo que se envía a un profesor exige un saludo y una despedida, no es un WhatsApp. Entonces, de un profesor se puede exigir determinadas cosas, pero no se puede exigir determinadas otras, como exigir que esté pendiente de ti las 24h de un domingo a las doce de la noche, que luego te contesta al correo, pero no tiene ninguna obligación. Eso lo dejo yo siempre muy claro, pero eso no impide la cercanía, que ellos sepan es una persona que está ahí para echarte una mano en todo aquello que tenga la obligación y el deber de hacerlo.

**Documento: 1. Arte y Humanidades\P 18 Arte Creencias**

**Peso: 0**

**Posición: 91 - 91**

**Código: 4. Acciones\Relación profesorado-alumnado\4.15. Importancia relación**

Entonces, en ese terreno, mi formación como docente, por ejemplo, me ha ayudado a la hora de plantearme que el alumno nunca es tu amigo, nuestro papel no es ser amigo del alumno. Por suerte para el alumno él puede escoger sus amigos y debe escogerlos, pero sí por lo menos para mi relación con los estudiantes, ¿no? una relación de más cercanía, de más contacto, a lo mejor de exigir no tanto en el aula, pero sí más fuera del aula.

**Documento: 1. Arte y Humanidades\P 18 Arte Diseños**

**Peso: 0**

**Posición: 32 - 33**

**Código: 4. Acciones\Relación profesorado-alumnado\4.15. Importancia relación**

E: En relación con la otra entrevista, comentaste que para ti era importante la relación con los otros estudiantes y que realmente, bueno, que cuando te preguntábamos cómo te caracterizas o cómo crees que te ven los estudiantes, pues sí que en algún momento dijiste como cercano, ¿no? Entonces, ¿qué prácticas o estrategias llevas a cabo para favorecer la relación con los estudiantes?

P. 18: Bueno, fuera del aula, hay una cosa, yo establezco los horarios de tutoría porque tengo que hacerlo, pero yo no creo en los horarios de tutorías regladas, es decir, para mí es fundamental que mi horario se acomode al del estudiante. Mis periodos de clase que se acomoden a los del estudiante. Es decir, si yo me reúno con un estudiante un viernes a las tres de la tarde porque es la única hora a la que él puede, pues yo lo hago. Otra cosa es que el estudiante quiera esa hora, pues yo qué sé, porque por la mañana quiera estar con su novia o novio. Pero es complicado, porque si el estudiante tiene clases, pues yo me acomodo en la medida de lo posible, claro, uno tiene también sus limitaciones. Siempre intento responder a los correos lo más rápido que pueda, siempre estoy dispuesto a cambiar el horario de tutoría si un estudiante me dice que no puede, es decir… Entonces, en ese sentido, digamos que mi receptividad para resolver una cuestión en el correo o por teléfono, el del despacho, no el mío personal, ellos saben que, si me dan una cuestión, les voy a contestar. Entonces, sí, si un estudiante en un momento dado me sorprende y tiene que venir a un examen y no aparece, pues busco su teléfono y le pregunto qué le ha pasado, ¿no? Y busco la alternativa para que pueda hacerle otro día. Y luego en clase, digamos que lo que hago siempre es intentar provocarlos, es decir, cuando veo que hay un tema que puede dar controversia, intento provocarlos, con lo cual, estoy asumiendo el riesgo de que me contradigan, porque ellos pueden plantear que me están contradiciendo no a lo que pienso realmente, sino a lo que yo les digo que estoy pensando, que es distinto, y por supuesto, valoro y ellos se dan cuenta de que estoy valorando su opinión. Es decir, yo no les digo “te voy a llevar la contraria porque creo que sabes menos que yo”, no, “te voy a llevar la contraria porque voy a intentar provocarte para que saques todo lo que se te ocurre y para que tus compañeros, además, discutan contigo”, ¿no? E intento que su opinión sea tan válida como la mía, con independencia de que esté formada o no. Y creo que eso es algo que ellos valoran de forma positiva.

**Documento: 1. Arte y Humanidades\P 20 Arte Diseños**

**Peso: 0**

**Posición: 122 - 133**

**Código: 4. Acciones\Relación profesorado-alumnado\4.15. Importancia relación**

E: Claro. Y pasando al plano de la relación profesor-alumno, ¿para ti es importante?

P. 20: Hombre, pues sí. Desde luego, no tanto como en primaria y secundaria, porque allí es mucho más importante. Aquí eso, en principio, pierde importancia, pero claro, no la pierde toda, sí que es importante que no tengan miedo, ¿no? Sobre todo, el miedo a preguntar, preguntar cosas que parezcan tonterías. “No os preocupéis, que, aunque parezca una burrada, no tengas miedo a pensar que vas a parecer tonto y tal”, o sea, convencerles de eso yo creo que es importante. Yo creo que nos pasa a todas las personas que siempre existe ese miedo a “voy a preguntar esto, pero a ver si es una tontería”, “pregunta hombre, que no pasa nada, lo normal es que, si te ha surgido a ti la duda, la tenga más gente”.

E: Sí, sí.

P. 20: Y aplaudir cuando ocurre eso, pues está muy bien, ¿no?

E: Yo siempre digo “me alegro de que hayas sido valiente y hayas planteado esa duda porque probablemente más gente la tenga”. Y los demás asienten.

P. 20: Claro, así es.

E: Y qué prácticas o estrategias llevas a cabo para favorecer la relación con tus alumnos, para romper un poquito ese miedo que me comentabas.

P. 20: Pues yo qué sé, una vez más el trabajo con poquita gente es importante. Si el grupo es pequeño es más fácil, si el grupo es grande, pues oye, aunque solo sea un poco, el trabajar con pequeños grupos y estar cinco o diez minutos con un grupo de seis o siete personas, pues creo que ayuda mucho, claro. Y luego, pues bueno, hacerles usar las tutorías, pero eso, la verdad es que no estoy satisfecho con lo que he conseguido, ¿no? Porque no termino de conseguir que vengan con más fluidez a las tutorías, ¿no?

E: Ahora te iba a preguntar sobre eso…

P. 20: Sí, también es verdad que es un poco una cuestión de horarios también.

E: Que muchas veces no coinciden.

P. 20: Claro. Y que tienen más asignaturas… En fin, tiene que ver con el agobio de tantos materiales, tantas asignaturas, las prisas, todo cuatrimestral… En fin, creo que tiene que ver con eso.

**Documento: 1. Arte y Humanidades\P 22 Arte Diseños**

**Peso: 0**

**Posición: 56 - 57**

**Código: 4. Acciones\Relación profesorado-alumnado\4.15. Importancia relación**

E: Antes te he preguntado por tu relación con tus estudiantes y me has dicho que para ti era importante, y que te comunicas con ellos por e-mail, aunque sean las dos de la mañana. Y también, me has dicho antes que tu relación con los alumnos influía en su aprendizaje, ¿no?

P. 22: Yo creo que, si tú te implicas con ellos, en cierto sentido, les obliga a ellos a implicarse un poco más por el miedo al qué dirán o qué pasará. Al final, se crea una amistad entre comilla que hace que haya más implicación, pero no por la nota, sino para no fallarte. Ya te digo, cuando corrijo los exámenes y me sacan una mala nota yo me sorprendo. Además, yo corrijo sin la ficha por delante, luego cuando pongo la nota ya pongo la ficha y no me lo creo.

**Documento: 1. Arte y Humanidades\P 23 Arte Diseños**

**Peso: 0**

**Posición: 33 - 33**

**Código: 4. Acciones\Relación profesorado-alumnado\4.15. Importancia relación**

P. 23: Yo tengo con ellos una continua relación, por ejemplo, en mis clases, lo normal es que haya una serie de ejercicios prácticos, en los que ellos están en contacto conmigo, vía e-mail…mi mujer tiene un cabreo de mil demonios, porque dice que estoy todo el día liado, pues sí, para eso me pagan y de hecho me pagan muy bien.

**Documento: 1. Arte y Humanidades\P 23 Arte Diseños**

**Peso: 0**

**Posición: 66 - 69**

**Código: 4. Acciones\Relación profesorado-alumnado\4.15. Importancia relación**

E: ¿Para ti, es importante la relación que se establece entre profesor y alumnado? ¿Por qué?

P. 23: Sí, es muy importante. Es importante porque se aprende mutuamente mucho.

E: ¿Qué prácticas o estrategias llevas a cabo para fomentar la relación con tu alumnado?

P. 23: Ninguna especial, insisto, yo creo que ahí es muy importante cómo tú aparezcas ante ellos. Por ejemplo, a mí, en primero ya me suelen decir Don Juan, y Don Juan me parece muy viejo, pero es que lo soy. Pero también, hay algunos que se atreven a decirme Juan y no tengo ningún problema. A mí, me da igual que me llamen como quieran. Yo sí les hablo de tú, no les hablo de usted. A mí, me parece que lo de usted de un profesor a un alumno, es una forma de distanciarlos, claramente. Entonces, yo sí les hablo a todo ellos de tú, y ellos me hablan como quieran, a veces tú, otras veces usted. Los de Erasmus europeo siempre me llaman profesor. Cada uno, tiene su forma, pero me da igual, ese tipo de asunto no me importa. Otra cosa que no me importa, para nada, es que teniendo en cuenta que estamos en la periferia de Sevilla y que mucha gente vive en el Aljarafe, y otras de otro sitio. A mí, no me importa para nada usar mi casa como despacho. No pasa anda, “mire usted, necesito hablar con usted en una tutoría, ¿cuándo tiene tiempo?”, “pues, pasado mañana por la tarde”, y yo le digo “pues yo pasado mañana no voy a ir a la facultad porque es una hora para ir, otra hora para volver, yo no sé conducir, entonces, le pregunto que dónde viven y les digo que se vengan a mi casa” ese tipo de familiaridad, ellos lo agradecen y lo agradecen mucho. O también, que tú le des tu teléfono, yo les doy el teléfono de mi casa…y la gente se sorprende porque les doy mi número, pero qué me van hacer mis alumnos. Entonces este tipo de asunto, les llama a ellos muchísimo la atención y yo intento llegar a una relación cordial con ellos.

**Documento: 1. Arte y Humanidades\P 24 Arte Diseños**

**Peso: 0**

**Posición: 76 - 81**

**Código: 4. Acciones\Relación profesorado-alumnado\4.15. Importancia relación**

E: En tu relación con el alumnado, ¿tú crees que es importante que haya una buena sintonía entre el estudiantado y tú?

P. 24.: Sí, yo creo que es fundamental. Me gusta trabajar en buena armonía, si es posible. Bueno y en estos casos, cuando ellos vienen a tutoría, si tú eres una persona que les das más confianza, pues supongo que ellos también la tienen.

E: ¿Cómo te comunicas con ellos? ¿Utilizas la plataforma virtual para comunicarte con ellos u otros medios?

P. 24.: A través del aula virtual, pues hablo con todo el estudiantado. Aunque, normalmente, te escriben al correo electrónico. Y también, en tutoría, en clase y en el pasillo.

E: ¿Cómo valoras tú tu relación con ellos?

P. 24.: Pues, en general es buena.

**Documento: 1. Arte y Humanidades\P 24 Arte Diseños**

**Peso: 0**

**Posición: 84 - 85**

**Código: 4. Acciones\Relación profesorado-alumnado\4.15. Importancia relación**

E: Tú consideras que si hay una relación digamos asimétrica entre el docente y el alumnado que no sea tan igualitaria o tan democrática, ¿crees que eso se debería cambiar dentro del aula?

P. 24.: A ver, yo creo que los roles tienen que estar claramente marcados entre el docente o la docente y el estudiantado. Pero eso, no implica que haya una relación cordial y accesible. Yo creo que al final, lo que demandan los estudiantes es que haya esa flexibilidad y que el docente se preste a sus inquietudes y a desarrollar sus dudas y les ayude en el proceso.

**Documento: 2. Ciencias e Ingeniería\P 25 Ciencias Diseños**

**Peso: 0**

**Posición: 68 - 71**

**Código: 4. Acciones\Relación profesorado-alumnado\4.15. Importancia relación**

E: Y, ¿para ti es importante la relación que se establece entre el alumnado y el profesorado?

P25: Pues sí, es muy importante, la confianza mutua, el aprecio… Todo, todo va llegando y todos recordamos a un profesor especialmente y no porque a lo mejor te explicara mejor que nadie, sino porque te ha llegado de otra manera, porque se ha interesado porque tú te enteres… Entonces, esas cosas se cuidan, claro.

E: Y, ¿qué estrategias llevas a cabo para fomentar la relación con tu alumnado?

P25: Bueno, pues el trabajo casi que, en colaboración desde el primer día, y luego procuramos también hacer actividades un poco fuera, tenemos una semana en arquitectura que es la semana blanca, que hacemos viajes, no siempre vienen todos, viene el que quiere o el que puede, pero con los que tenemos más experiencia, pues es una cosa más personal, se establecen otro tipo de…claro, de vínculos y es muy importante. Y cuando esa fecha es tempranita en el curso va muy bien, porque se pierden todos los miedos que son unos frenos muy importantes para esta actividad.

**Documento: 2. Ciencias e Ingeniería\P 26 Ciencias Diseños**

**Peso: 0**

**Posición: 76 - 79**

**Código: 4. Acciones\Relación profesorado-alumnado\4.15. Importancia relación**

E: Vamos a hablar ahora de la relación profesor-alumno, ¿para ti es importante la relación que se establece entre el estudiante y el profesor?, y ¿por qué?

P26: Para mí lo que es importante es que haya una relación de confianza, porque si…yo me pongo en el papel del estudiante, si tú confías en el docente que tienes delante, aprendes. Si el docente que tienes delante no te da una relación de confianza, pues ya tienes reticencias, y, entonces, yo creo que sí que es importante. Hombre, antes lo tenía más fácil, porque antes era más jovencito, entraba en clase, parecía un estudiante más… Desde hace algunos años ya, que me empiezan a hablar de usted, digo “uf”, chungo, ¿no? Ese salto generacional sí que empiezo a notarlo ya y me parece un pequeño hándicap, ¿no?, pero bueno, yo intento que aquí vengamos todos a trabajar, no soy yo el malo que te obliga a estudiar y luego te va a poner un cero en el examen, sino que tu vienes porque quieres aprovechar mejor algunos conocimientos que yo tengo para que te guíen a la hora de elaborar los tuyos, ¿no?, entonces, yo creo que, esa relación, es una relación de confianza distendida, que te puedan decir claramente lo que piensan o…yo creo que está bien.

E: Y, ¿qué serie de estrategias llevas a cabo para favorecer la relación con tus estudiantes?, ¿haces algo especial o…?

P26: Yo intento tratar a todo el mundo por igual, yo trato a mis estudiantes igual que a ti, no sé, lo cual algunos me recriminan, algunos profesores de mi departamento dicen que, bueno, “es que irte con los estudiantes a tomar un café, eso está muy mal”, y digo, “bueno, también me voy contigo, ¿no?”. No hago…

**Documento: 2. Ciencias e Ingeniería\P 27 Ciencias Diseños**

**Peso: 0**

**Posición: 50 - 53**

**Código: 4. Acciones\Relación profesorado-alumnado\4.15. Importancia relación**

E: Bueno, con respecto a la relación profesor-alumnado, ¿para ti es importante?

P27: Sí, me parece importantísimo porque la transmisión de conocimientos se basa en la confianza, en la percepción que tenemos los unos de los otros… Entonces, me parece muy importante.

E: ¿Qué medios utilizas para comunicarte con tu alumnado?

P27: Pues ninguno en concreto. Yo quiero que mis alumnos me perciban como una persona que hace bien su trabajo, que le importa su trabajo y que los considera.

**Documento: 2. Ciencias e Ingeniería\P 27 Ciencias Diseños**

**Peso: 0**

**Posición: 56 - 57**

**Código: 4. Acciones\Relación profesorado-alumnado\4.15. Importancia relación**

E: Y, cómo valoras tú tu relación con los estudiantes.

P27: Bueno, yo espero que sea una buena relación y lo que sé, vamos, lo que me llega es que es buena. Es decir, el hecho de que yo me considere satisfecho de mi actividad docente, en términos generales, se basa en la experiencia que tengo como docente, que, por las evaluaciones, he tenido algún premio de docencia, etc. Veo que es positiva, de hecho, este año no ha ocurrido, pero el año pasado, un alumno me mandó un correo, a veces ocurre, ¿no? Bueno, la parte de la evaluación, la opinión y tal, y son buenos, o por lo menos no he tenido nunca ningún correo de estos en los que me pongan a caldo ni nada de esto.

**Documento: 2. Ciencias e Ingeniería\P 27 Ciencias Diseños**

**Peso: 0**

**Posición: 60 - 61**

**Código: 4. Acciones\Relación profesorado-alumnado\4.15. Importancia relación**

E: ¿Consideras que se debe cambiar el modo en cómo un estudiante se relaciona hoy en día con su alumnado?

P27: Sí, bueno, no sé si se puede cambiar. De todas formas, la manera en la que nos relacionamos con el alumnado, o sea, yo no creo que se pueda cambiar, depende mucho del carácter, de la profesionalidad del profesor, etc. Yo no veo que eso se vaya a poder cambiar. Realmente, es que son relaciones personales, no veo que ahí se puedan hacer muchos cambios. Yo, desde luego, soy partidario de…cuando fui alumno, los profesores que aprecié fueron muy profesionales, muy serios, muy poco arbitrarios… Todo lo que…ya cuando era estudiante me quedé un poco vacunado a todo este…a veces un poco de colegueo y todo esto, yo no, ni me gustó cuando era estudiante, no aprecié para nada a los profesores con ese tipo de…y desde luego, no lo hago como docente.

**Documento: 2. Ciencias e Ingeniería\P 27 Ciencias Diseños**

**Peso: 0**

**Posición: 62 - 63**

**Código: 4. Acciones\Relación profesorado-alumnado\4.15. Importancia relación**

E: Entonces, cómo crees que tiene que ser la relación del profesorado y el alumnado para que influya positivamente.

P27: Bueno, en primer lugar, se tiene que basar en el respeto mutuo y luego en la profesionalidad, en el sentido de que realmente la actividad docente sea una cosa seria, robusta, que estemos todos implicados… Todo eso es muy importante.

**Documento: 2. Ciencias e Ingeniería\P 29 Ciencias Diseños**

**Peso: 0**

**Posición: 50 - 53**

**Código: 4. Acciones\Relación profesorado-alumnado\4.15. Importancia relación**

E: ¿Para ti es importante la relación que se establece entre el alumnado y el profesorado?

P29: Esto es como el psicólogo que le dice tengo que tener una distancia con el paciente…obviamente hay que ser objetivos, pero hay que establecer un vínculo. Es fundamental tener un vínculo con el alumnado, que ellos vean que pueden contar contigo y que tú estás ahí. Yo a veces escucho las movidas de mi alumnado que no tienen nada que ver con mi asignatura, pero lo hago para que se desahoguen. Entonces, es muy importante ya que si confías en una persona lo que te dice tiene un mayor pavor. Entonces, sí que hay que establecer un poco de empatía.

E: ¿Qué prácticas y estrategias llevas a cabo para fomentar esta relación con el alumnado?

P29: Hombre, pues en las prácticas de laboratorio se nota. Es que he tenido años en los que no ha habido prácticas de laboratorio y las evaluaciones que he tenido por parte de ellos han sido más bajas. Y cuando he tenido estas prácticas, pues al ser grupos más reducidos, es más personal y da mucho más pie a las bromitas. Entonces, es aquí cuando ven la normalidad del profesor de universidad, y que no somos todos unos siesos ni nada por el estilo como nos pintan. Entonces, en las primeras clases me miran con mucho respeto y sé que los tengo muy asustados. Entonces, intento hacer las clases muy amenas y con cosas de la vida cotidiana y demás. Además, les hago bromitas, es que sé llevármelo a mi terreno. En definitiva, ser serio pero siendo natural. También, hay que mostrar que la asignatura te gusta y que vienes con ganas.

**Documento: 2. Ciencias e Ingeniería\P 30 Ciencias Diseños**

**Peso: 0**

**Posición: 82 - 85**

**Código: 4. Acciones\Relación profesorado-alumnado\4.15. Importancia relación**

E: Ya, vale. Y alguna cosita más, porque nos gustaría saber más sobre la relación entre profesorado y alumnado. Entonces, para ti, ¿es importante la relación que se establece entre el alumnado y el profesorado?

P30: Sí y no. Sí porque si te odian tus alumnos, pues yo creo que va a ser todo más complicado. Tiene que haber una relación cordial, no tienen por qué ser sus amigos, soy un profesor, pero tiene que ser una relación normal, cordial, lo más práctica posible, pero cordial. Y eso es lo más importante porque la receptividad cambia. No es igual “yo paso de este tío, no lo aguanto”, no es la misma receptividad y, por tanto, yo creo que tener ese pequeño grado de cordialidad es importante, que no es esencial. No pretendo ser amigo de nadie, ni de ninguno de mis alumnos ni pretendo que tengan un póster mío en casa. Yo solo quiero que estén receptivos a la información que les ofrezco y que lo hagan lo mejor posible. Luego chico, ya está, seguimos cada uno nuestra vida y ya está, que no haya ningún problema ni ninguna rencilla y ya está, que pueden venir de donde menos te lo esperes. Una frase sacada de contexto y ya, te hacen una cruz. Yo intento siempre tener esa precaución de ser lo más escéptico posible.

E: ¿Y para fomentar la relación con tu alumnado sigues algunas prácticas o estrategias concretas?

P30: Pues ser lo más accesible posible, entonces, si necesitan algo de mí. Yo explico en mis tutorías virtuales, pero también tengo un horario de tutorías y el horario de tutorías está marcado en el calendario, pero entiendo que ni los alumnos ni yo puedan adaptarse estrictamente al horario de tutorías, entonces digo “el horario es ese porque me obligan a ponerlo, pero en cualquier momento que necesitéis una tutoría presencial quedáis conmigo y ya está”. Que igual el día que tengo programadas las tutorías pues tengo otra cosilla e igual no puedo ir y entonces les digo que queden conmigo y se lo repito a lo largo del semestre cosa que hasta el día de hoy ha funcionado muy bien.

**Documento: 2. Ciencias e Ingeniería\P 31 Ciencias Diseños**

**Peso: 0**

**Posición: 172 - 185**

**Código: 4. Acciones\Relación profesorado-alumnado\4.15. Importancia relación**

E: Bueno, vamos a pasar a otra cuestión, ¿para ti es importante la relación profesorado-alumno?

P31: Para mí es esencial, si no tienes relación, no te comunicas, y si no te comunicas, no puedes transmitir nada, ningún conocimiento ni nada. Lo que sí que es verdad es que es muy difícil cuando tienes grupos muy grandes. Entonces, al final, la relación, digamos, se hace más en las prácticas y en las actividades dirigidas que en clase. En clase, en realidad, solo te relacionas con los que te responden cuando tú estás preguntando, y con los poquitos que te vas quedando con las caras y eso…

E: Entonces, ¿utilizas alguna estrategia para fomentar la relación con el alumnado?

P31: No.

E: Aunque tú creas que no…

P31: No sé yo de ninguna estrategia para fomentar la relación. De hecho, a lo mejor se relacionan conmigo más de la cuenta, a través de los foros, del correo electrónico…

E: Una estrategia puede ser, por ponerme en ese nivel, tratar temas que a ellos le interesen…

P31: Bueno sí, eso sí, eso sí lo hago…

E: Porque tú me has dicho que cuentas chistes…

P31: Cuento chistes, anécdotas curiosas…

E: Pues eso son las estrategias.

P31: Y, a lo mejor pierdo un rato de la clase para contarles eso que se les queda a todos…

E: Sí, sí, pues todo eso que estamos hablando son estrategias.

P31: Ah mira, pues sí.

**Documento: 2. Ciencias e Ingeniería\P 32 Ciencias Diseños**

**Peso: 0**

**Posición: 48 - 49**

**Código: 4. Acciones\Relación profesorado-alumnado\4.15. Importancia relación**

E: Y si hablamos ahora de la relación, ¿para ti es importante la relación profesor-alumno?

P32: Yo creo que vean que si tienen algún comentario o duda que te lo puedan hacer, y que seas accesible, yo creo que es positivo. Ya lo de ser amigos y colegas creo que no toca, pero a lo mejor sí que estudiantes que han hecho el trabajo de fin de grado y has visto que se esfuerzan y hacen mucho, pues a lo mejor haces más de la cuenta. Alguien que…y a lo mejor sí que llegas, y luego hacen la tesis o sí que tienes un poco más de relación a nivel personal, pero a nivel de profesor, con que vean que eres accesible o, yo qué sé, cualquier comentario te lo pueden hacer sin verse ellos forzados o de no decirlo por si pueda haber represalias o alguna cosa de esas, pues, está bien.

**Documento: 2. Ciencias e Ingeniería\P 33 Ciencias Diseños**

**Peso: 0**

**Posición: 36 - 37**

**Código: 4. Acciones\Relación profesorado-alumnado\4.15. Importancia relación**

E: Si hablamos ahora de la relación alumno-profesor, me gustaría saber hasta qué punto esa relación es importante para ti y qué tipo de relación tienes con tus alumnos.

P33: Pues, para mí desde luego es muy importante que la relación sea buena, sea fluida y que haya buena comunicación. A mí me gusta que los alumnos sean conscientes de que yo les quiero ayudar en todo lo que esté a mi alcance, que no crean que yo lo que quiero es ponerles trabas, sino que es todo lo contrario. Entonces, les intento dar la confianza suficiente para que ellos en clase pues pregunte. Lo que no me gusta es caer en una relación demasiado cercana, cordial, sino que intento mantener esa distancia y creo que es bueno. Además, creo que la mejor forma para que ellos lo vean a través del ejemplo, si ellos ven que tú le ayudas, pues entonces yo creo que como mejor se predica es con el ejemplo. No sé si ellos lo perciben así, pero ésta es mi intención y además, yo en clase lo digo que me pregunten que yo estoy aquí para ayudarles.

**Documento: 2. Ciencias e Ingeniería\P 34 Ciencias Diseños**

**Peso: 0**

**Posición: 42 - 43**

**Código: 4. Acciones\Relación profesorado-alumnado\4.15. Importancia relación**

E: Y sobre la relación profesor-alumno, ¿tú crees que es importante la relación que tienes con ellos para su aprendizaje?

P34: Hombre, supongo que sí que si intentas tener un poco un trato más cercano, pues supongo que la gente, tendrá menos miedo entre comillas a venir a lo mejor a tutorías a preguntar y eso, que quieras que no, tiene que favorecer el aprendizaje, pero quitando esa parte, pues la actitud que tengas con el alumno, siempre que sea de respeto y tal, no va a influir mucho más, pero si consigues un poco más de cada uno, pues a lo mejor hay gente que tiene menos problema de venir a tutorías o a preguntar dudas incluso en clase que cuando alguien impone un poco más de respeto o de miedo, entre comillas, ¿no? A lo mejor la gente no pregunta si tiene dudas de la asignatura. Pero, yo creo que a veces es más el que a ellos les guste el contenido de la asignatura que eso, ¿no? Hay gente que no le gusta la asignatura porque a lo mejor no les gusta ese temario y entonces tienden a trabajar otras asignaturas que les gustan más y eso influye en el aprendizaje, claro, no te motiva, el aprendizaje va a ser mucho más difícil y más lento, ¿no?

**Documento: 2. Ciencias e Ingeniería\P 35 Ciencias Creencias**

**Peso: 0**

**Posición: 36 - 37**

**Código: 4. Acciones\Relación profesorado-alumnado\4.15. Importancia relación**

E: Entonces, podemos decir que te caracteriza el ser un profesor cercano al alumnado.

P35: Sí, lo que pasa que siempre hay que mantener…yo es que tengo esa estrategia. La cercanía la exploto en las clases de laboratorio, ya que estoy junto al alumno y hay una relación directa o sino, en las tutorías.

**Documento: 2. Ciencias e Ingeniería\P 35 Ciencias Diseños**

**Peso: 0**

**Posición: 64 - 73**

**Código: 4. Acciones\Relación profesorado-alumnado\4.15. Importancia relación**

E: Para el estudiantado con discapacidad, ¿hay alguna de las metodologías docentes que utilizas que sea más efectivas?

P35: Pues, yo creo que las mismas porque los discapacitados siempre tienen el riesgo de que se sientan excluidos. Así que por eso hay que utilizar las mismas.

E: ¿Es importante la relación que se establece entre el profesor y el alumnado?

P35: Sí, por supuesto.

E: ¿Por qué piensas esto?

P35: Sí hay que conectar, pero esto no significa que haya que ir de copas o de fiesta de ellos. Pero, dentro del campus la relación personal es muy importante, ya que los alumnos tienen que sentirse en el mismo plano que los profesores.

E: ¿Por qué piensas esto?

P35: Porque así se facilita la integración y la inclusión sean ya capacitados o discapacitados. Es que si a un alumno le hablas sin establecer un plano de nivel distinto, pues te van a responder aún mejor.

E: ¿Qué prácticas y estrategias llevas a cabo para fomentar tus relaciones con tu alumnado?

P35: Pues, sobre todo establecer la discusión y hacer preguntas en clase, pues yo lo que hago es moverme por los pasillos, no me quedo en la tarima o en la mesa, sino que me acerco al alumno para que se sientan cómodos y para que no tengan vergüenza a la hora de responder. A veces esto lo consigo y otras veces, pues no lo consigo.

**Documento: 2. Ciencias e Ingeniería\P 36 Ciencias Creencias**

**Peso: 0**

**Posición: 56 - 56**

**Código: 4. Acciones\Relación profesorado-alumnado\4.15. Importancia relación**

P36: Bueno, yo creo que las que te he dicho, porque yo estoy hablando por mí mismo, o sea, simplemente, tiene que tener, sobre todo, empatía por los estudiantes, hasta cierto punto, porque luego también tienes que tener autoridad, porque tú eres el profesor y ellos son los alumnos, o sea, mis alumnos no tienen mi teléfono, hay algunos que, bueno, lo podrían tener, pero, en realidad, no tengo la comunicación de que tengan mi teléfono ni me voy de copas con ellos, o sea, está claro que yo soy el profesor y ellos son los alumnos. O sea, no es exactamente…puedes ser cercano, pero no soy coleguita, hay una gran diferencia.

**Documento: 2. Ciencias e Ingeniería\P 36 Ciencias Diseños**

**Peso: 0**

**Posición: 82 - 85**

**Código: 4. Acciones\Relación profesorado-alumnado\4.15. Importancia relación**

E: Vamos a hablar ahora de un tema que me has dicho también que es importante para ti, el tema de la relación entre tú como profesor y los estudiantes, ¿vale? Para ti, ¿es importante la relación que hay entre un estudiante y un profesor?

P36: Sí, sí, lo es. Es una relación humana, tú simplemente estás intercambiando algo, o sea, intercambiando conocimientos por esfuerzo para aprenderlos. Entonces, es importante el saberte el nombre de los estudiantes, el reconocerlos, y simplemente, una cuestión también de crear un buen ambiente en la clase. A mí me molesta mucho que me hablen en la clase y no es lo mismo decir “dejadme en paz porque…”, que decir “Pepito, cállate porque me estás molestando”, porque no lo toman como algo… dicen “ah, vale, perdón”, es la respuesta que esperas, no esperas intimidar a nadie, no quieres intimidar a nadie.

E: Y, ¿qué es lo que haces tú para fomentar esa buena relación con los estudiantes?

P36: Por ejemplo, ya te lo he dicho, llamarle a cada uno por su nombre, aprendérmelos, que me cuesta un montón. No les hago fotos el primer día, entonces, tengo una foto de cada uno con el nombre, luego me las estudio. O sea que, normalmente, hago algo de trampa ahí.

**Documento: 2. Ciencias e Ingeniería\P 37 Ciencias Diseños**

**Peso: 0**

**Posición: 46 - 49**

**Código: 4. Acciones\Relación profesorado-alumnado\4.15. Importancia relación**

E: Muy bien. Vamos a hablar ahora de la relación entre profesor y alumno. ¿Para ti es importante la relación que se establece entre los estudiantes y el profesorado? Y, ¿por qué?

P37: Yo creo que los alumnos deben confiar en el profesor en el sentido de confiar en lo que les cuenta. Y en que lo que les está contando es verdad. Si un alumno no confía o no se cree al profesor, no va a ser capaz de asimilar lo que el profesor le está contando. Entonces, hay que llegar un poco con pies de plomo y asegurar que lo que tú estás contando allí es verdad para conseguir que los alumnos te crean y confíen en ti en el sentido en el que lo que tú estás contando es verdad. Yo personalmente soy bastante abierta en la relación con los alumnos. Yo no les hablo de usted. Soy incapaz. Yo tengo compañeros que hablan a los alumnos de usted. Compañeros más jóvenes que yo que les hablan a los alumnos de usted por aquello de crear la...

E: La distancia.

P37: La distancia, que también creo que es necesario. Crear una... O sea, podemos acercarnos, pero cuidadito, que aquí el profesor soy yo. Entonces, yo les hablo de tú, les dejo que me llamen por mi nombre y que se dirijan a mí por mi nombre, pero no como “oye, tú”. “Oye tú”, no. Puedes decir “mira P37, que no sé qué...”. Entonces, guardando un poquito de distancias, yo creo que soy bastante cercana a los alumnos. Yo, verás... que les invito a mi despacho siempre que lo necesiten. Verás, los intento tratar como a mí me gustaría que me tratasen simplemente.

**Documento: 2. Ciencias e Ingeniería\P 37 Ciencias Diseños**

**Peso: 0**

**Posición: 50 - 51**

**Código: 4. Acciones\Relación profesorado-alumnado\4.15. Importancia relación**

E: Claro. Y, ¿qué prácticas o que estrategias llevas a cabo para fomentar la relación con tu alumnado? Si haces algo especial para fomentar esa relación.

P37: La verdad es que no. No. Yo creo que estas cosas van un poco en la actitud personal de las personas, valga la redundancia. Yo no hago nada especial. Yo llego, me presento, les cuento de qué va la asignatura... Sí que es verdad que ellos notan un poquito más de cercanía cuando, como te he dicho antes, les cuentas historias. Y yo les cuento historias, Ya te digo, a veces reales, a veces inventadas, pero intento sacarles de cada tema alguna cosa en particular. O alguna diapositiva que esté relacionada, ¿no? Ahora lo de la serie de *The Big Bang Theory* nos ayuda mucho, oye. Y a veces pone una diapositiva con los personajes porque en un capítulo hablaron de esto que estamos comentando ahora. Y yo creo que esa manera de dar las clases un poco más distendida, ayuda a que los alumnos se acerquen más a ti.

**Documento: 2. Ciencias e Ingeniería\P 38 Ciencias Creencias**

**Peso: 0**

**Posición: 107 - 107**

**Código: 4. Acciones\Relación profesorado-alumnado\4.15. Importancia relación**

P38: No, no, pero es porque la universidad ha cambiado Víctor, seguro que tú la ves de manera diferente a cómo te la enseñaron a ti, ¿a que sí?, ¿a que tú intentas estar más próximo a tus alumnos? Y no es que te tengan menos respeto del que tú tenías a tus profesores, porque tú había profesores a los que ni si quiera ibas a tutorías, ¿verdad?, ni si quiera le mirabas a los ojos, y a lo mejor no era problema del profesor, ¿eh?, a lo mejor era problema del entorno, ellos y nosotros tampoco nos relacionábamos normalmente con ellos. Ahora decimos, “es que son unos desvergonzados”, no, es que las relaciones personales han cambiado también, y eso se nota. Entonces, la manera de enseñar y de percibir el proceso de enseñanza-aprendizaje ha cambiado, pero es que la sociedad ha cambiado, afortunadamente. Hemos pasado un poco de vergüenza si no se controla, pero yo creo que es mejor, porque si quieres enseñar y que aprendan tienen que sentirte próximo. Eso no quiere decir que te vean como un coleguita, y te puedes tomar cervezas con ellos, pero no eres su coleguita, no lo eres, pero no tienes por qué estar encima de una tarima inaccesible, ¿sabes? Entonces, ahora la red falla por nosotros.

**Documento: 2. Ciencias e Ingeniería\P 38 Ciencias Diseños**

**Peso: 0**

**Posición: 190 - 205**

**Código: 4. Acciones\Relación profesorado-alumnado\4.15. Importancia relación**

E: Sí, sí. Vamos a hablar ahora de algo que ya has comentado antes varias cosas, que es la relación entre el profesorado y el estudiante, la relación profesorado-alumnado, ¿para ti es importante la relación que se establece entre alumno y profesor?, y ¿por qué crees tú eso?

P38: Por supuesto, por supuesto. Tiene que ser totalmente fluida, empática, y bidireccional, ¿eh?, yo necesito una respuesta. Cada respuesta que tú me des, yo voy a volver a interaccionar contigo. Yo eso lo necesito, y proximidad, yo necesito proximidad con los alumnos, yo, personalmente. También la proximidad con la suficiente distancia, ¿sabes? A mí me gusta que me cuenten sus cosas, yo les acabo cogiendo…ellos no lo saben, pero les acabo cogiendo cariño y te acuerdas de ellos, te acuerdas mucho de ellos, los ves en cuarto y te dicen “¡hola P38!”. Mira, me pasó una vez una cosa muy graciosa, estaba comiendo con mi familia, en Bormujos, en la Huerta de San José, no sé si has estado, es una finca muy bonita, y un camarero que estaba allí me viene y me dice “¿no te acuerdas de mí P38?”, y digo “no”, y me dice “sí, estuve contigo en fundamentos de ciencias de la materia, soy Roque”, digo, “ya está, grupo seis, por la tarde, sacaste un nueve y pico”, claro, rápidamente lo ubiqué, le dije “sí, estabas en el grupo seis, te fue bien, sacaste un nueve y pico”, mira, se quedó, estaba ya por cuarto, pero me acordé. Claro, también es verdad que era un nombre particular, no he tenido ningún Roque, pero digo, y sé los alumnos del grupo en el que estaba, digo los alumnos de su pequeño grupito de trabajo.

E: Sí, sí.

P38: Entonces, te agrada que ellos se acuerden de ti, pero también a ellos les agrada que te acuerdes de ellos.

E: Sí.

P38: En las prácticas de aquí de laboratorio de química son nueve días. Yo me obligo entre el primer y el segundo día, para el tercero me tengo que saber el nombre de los doce. Entonces, cuando les voy recogiendo los papeles, si son niño y niña lo tengo fácil, por los puestos, pero si son dos niñas digo ¿quién es María y quién es Ana? Ana con gafas, María sin gafas. Busco algo, porque luego al tercer día “oye María”, ya los ubico en ese puesto, pero les gusta, les gusta Víctor. No son números, son personas.

E: Sí, sí, es verdad.

P38: Son personas que van allí, que van a estar un tiempo y quieren sentirse que son ellos, que no son uno más del cual no sabes su nombre. A mí me hubiera gustado que se supieran mi nombre.

E: Y, ¿llevas algún tipo de práctica o de estrategia concreta para favorecer o facilitar la relación con tus estudiantes?

P38: Bueno, no me sale, no es pensada, esto que te digo de los alumnos, esto de aprenderme sus nombres. En la clase también lo intento, ¿eh?, porque al principio te vas conociendo la primera fila… Ah, sí les digo que se presenten, sí les digo las normas, les digo que ellos saben mi nombre pero que yo no sé el suyo, así que juego en desventaja. Son muchos y yo soy una y conocida. Así que si no se presentan les digo, “perdona, ¿tú eras…?”, “María”, “Ah”. Pero luego también un poco yo por las fichas miro, miro la ficha, les obligo a que me den fichas, “es que están en Internet”, es que muchas veces las fotos que están en Internet…

E: No tienen nada que ver.

P38: Entonces, o tienen el gorro así puesto, o salen así de chiquititos con la Torre Eiffel detrás…sí, es que es verdad, o sujetando la torre de Pisa, es decir, que no se les ve. Entonces, les voy pidiendo una foto y voy viendo su…este es de letras, este de ciencias, y voy viendo sus caras ahí, es decir, que intento conocerlos mucho. Y, con los partes de asistencia, a lo mejor les pregunto y les digo “venga, hoy voy a chequear”, y no es chequear, a lo mejor es asociar un nombre con una cara…dedico ratitos de esos, pero siempre voy preguntando el nombre, y la tercera o cuarta vez ya les digo, “Ah, venga, Inés, qué quieres preguntar”, y se quedan, claro. Pero, ya ellos van viendo…

E: Sí, son iniciativas.

P38: Claro, es que el grupo…eso se lo digo muchas veces, “el grupo no soy yo, somos todos y esto es trabajo de todos”, eso siempre se lo digo, pero claro, siempre hay alguno que se me pasa su nombre, es que claro, somos muchos, somos muchos Víctor. Aquí que van menos a clase…pero es que allí van muchos a clase, ¿eh?, lo cual es bueno, y tú les dices…un año me enfadé mucho y estuve dos días sin pasar parte, y les dije que no lo iba a pasar más porque no paraban. Y, además que me vieron salir muy, muy, muy enfadada, diez minutos antes cerré… Esa es la cosa, que no confraternizo porque no creo que deba, pero sí establecer un clima de confianza y un clima cálido. Pero hay alumnos que eso lo confunden, entonces, hay veces que tienes que pegar un carpetazo, eso me ha pasado un año solo, que me fui diez minutos antes sin decir nada, y dije “señores, la clase se ha terminado”, y al día siguiente, volví, y volví incluso un poquito más delante de donde lo había dejado, pero ni chistaron, lo hice a propósito.

E: Todos se dieron cuenta.

P38: Ni chistaron, ¿eh?, y les dije que habíamos finalizado ya los partes de asistencia y que la prebenda se había terminado, y me duró tres días el enfado, y luego ya…yo no puedo estar permanentemente enfadada, ya les dije “¿hemos aprendido algo?”, como a los niños, les dije “¿retomamos nuestras viejas costumbres o continuamos con las nuevas?”, “no, no, P38, de verdad”, y la cosa fue bien, pero algunas de esas se viven, ¿no? Pero no me sale programado, lo hago, cada año una cosa. Lo de presentarse sí, eso sí les digo mucho “presentaos”, o “tú eras…que no me acuerdo”, y van ya…

**Documento: 3. Ciencias de la Salud\P39 Salud Diseños**

**Peso: 0**

**Posición: 39 - 39**

**Código: 4. Acciones\Relación profesorado-alumnado\4.15. Importancia relación**

Yo es que tengo muy buena relación con ellos porque yo no me comporto como el típico profesor que deja una barrera entre ellos, yo es que me considero más un profesional de la sanidad, un enfermero que profesor…lo único que intento es transmitirles lo que yo sé de la mejor manera posible para que aprendan. Entonces, claro, los alumnos me ven cuando han terminado la carrera…yo tengo compañeros míos que han sido alumnos míos…

**Documento: 3. Ciencias de la Salud\P39 Salud Diseños**

**Peso: 0**

**Posición: 64 - 65**

**Código: 4. Acciones\Relación profesorado-alumnado\4.15. Importancia relación**

E: En cuanto a la relación del profesor con el alumnado, ¿para ti es importante la relación que se establece entre el alumno y el profesor? y ¿qué prácticas o estrategias llevas a cabo para fomentar esa relación con el alumnado?

P39: En primer lugar, hay que ser muy honesto con el alumno, no intentes nunca aparentar lo que no sabes. Yo creo que un profesor que no sabe o que no tiene experiencia sobre lo que va a explicar, creo que, por propia honestidad, no debería de hacerlo. No creo que cualquier profesor…como me decía la directora de mi escuela, cualquier profesor que entre tiene que estar preparado para dar cualquiera asignatura, y yo empecé a reírme. Primero, ser muy honesto con el alumno y si eres honesto, explicas bien y le facilites al alumno el aprendizaje, ya te ganas el respeto.

**Documento: 3. Ciencias de la Salud\P40 Salud Diseños**

**Peso: 0**

**Posición: 74 - 79**

**Código: 4. Acciones\Relación profesorado-alumnado\4.15. Importancia relación**

E: En relación con la relación del profesorado con el alumno para ti, ¿es importante la relación que se establece entre el alumno y el profesor?

P40: Eso es fundamental.

E: ¿Por qué?

P40: Yo soy consciente que a ciento cuarenta alumnos no se llega. Yo soy consciente, pero a los veinte o treinta alumnos que han tenido contacto contigo, que han ido a tutorías, a esos les llega perfectamente, y a veces, esos son los que son imprescindibles para muchas cosas, esos son los que al final te encuentras de supervisor en Francia o en Inglaterra, porque son los que… a ver cuántas veces les he dicho a los alumnos, “mira el alumno que entre aquí con un ocho y salga con un ocho y medio, no me interesa; me interesa más aquél que entra con un cinco y sale con un siete porque por lo menos ha tenido más capacidad que el otro, porque de ocho a ocho y medio solo hay medio punto, así que eso es fundamental y hay que buscarnos los recursos y hay…”. Bueno, pues si tú llegas a un grupo de alumno con los que tienes cierta relación, los que te preguntan en cafetería, los que te dicen “profesor tienes algún tiempo para mí porque me gustaría que…”, eso es fundamental. El alumno que no te conoce, que viene a clase por pura dinámica, que a las tutorías no va, esos alumnos…algunos si aprueban y pueden sacar hasta un diez, pero no creo que sean los que necesitamos en enfermería.

E: ¿Qué estrategias utilizas tú para fomentar esa relación con el alumnado?

P40: Yo no me levanto por las mañanas pensando mira hoy voy hacer todo esto, no tengo una estrategia concreta, sino que va conmigo. No tengo una estrategia concreta, sino que va conmigo. La estrategia que tengo es que me gusta la docencia y me gusta que aprendan y no me importa perder el tiempo para responder una pregunta ni perder el tiempo para quedar y darle una clase particular a un grupo de alumnos, o incluso alumnos que no han venido desde segundo o tercero y me digan “Paco mira vamos a empezar el digestivo y por qué no nos das una charla de esta parte del estómago”, pues yo se la doy…bueno también tengo una familia que me ayuda porque si voy por la mañana y por la tarde y me llevo todo el día en el trabajo…pero yo creo que va conmigo, que no tengo ninguna estrategia concreta, estoy abierto, estoy asequible, disponible para adaptarme y cuando he tenido problemas de tiempo he buscado otro tiempo y ya está.

**Documento: 3. Ciencias de la Salud\P40 Salud Diseños**

**Peso: 0**

**Posición: 82 - 83**

**Código: 4. Acciones\Relación profesorado-alumnado\4.15. Importancia relación**

E: Claro, y todo eso fue debido porque tú lo conoces, por la relación que tenías con él.

P40: Claro, porque tú vas viendo. Verás, yo sé cómo enfoco mi asignatura y, además, yo pregunto a mis alumnos cómo les van en otras asignaturas. Entonces, si tú no te preocupas de eso…no sé…si me preocupo de mi asignatura nada más, pues de mi currículum sabré mucho, pero de lo que es mi profesión, la enfermería, de esto voy a saber bien poco. Pues lo mismo es, si tú no te preocupas…si un alumno que ha sacado sobresaliente en todas las asignaturas y en la tuya saca un cinco pelado, nos tenemos que replantear qué ha pasado, o bien no he llegado el alumno, o no me he sabido explicar…y en los exámenes de tipo test, hay preguntas que no contesta ningún alumno y yo las quito, pero ¿por qué? las quito, pues probablemente las quito porque el problema es mío, o que esté mal redactada la pregunta o que no la haya explicado bien; también cabe la posibilidad de que el alumno no se lo haya estudiado, pero que ningún alumno te haya contestado a una pregunta…y eso ¿qué requiere? Que tú tengas las notas y tú veas en el tipo test cuáles son aquellas que no han sido contestadas. ¿Eso qué es? Tiempo tuyo, sí pero si eso lo ve el alumno, de alguna forma se crea un vínculo importante.

**Documento: 3. Ciencias de la Salud\P41 Salud Diseños**

**Peso: 0**

**Posición: 100 - 105**

**Código: 4. Acciones\Relación profesorado-alumnado\4.15. Importancia relación**

E: Muy bien. Vamos a hablar un poco, aunque ya han salido pinceladas, pero vamos a profundizar en la relación profesor-alumno ¿Es importante para ti la relación que se establece entre tú como profesor y los estudiantes?

P41: Sí, sí, totalmente, porque la actitud del alumno cambia completamente. Cuando tú sabes que, cuando el alumno llega desde primero y le ha preguntado al de tercero y le ha preguntado al de cuarto, cómo es esta persona, ya tú lo notas rápidamente, y notas que la persona, al principio, viene en defensa, viene con su escudo, no se abre, no es receptivo, y eso se nota rápidamente. Entonces, la relación es fundamental porque tu clase va a ser todo eso. A no ser que tengas un paradigma tan positivista que yo doy clases, tú tomas apuntes y al examen. Entonces, yo me callo, si tú vienes a dar una clase magistral, para coger apuntes y después volcarlo en un examen, entonces ya… Ahí no tienes nada que hacer, ¿no? Yo soy más de un paradigma socio-crítico, que no solamente es trabajar y aprender, sino que ahora lo llevo a cabo, lo llevo a cabo y veo dónde me he equivocado y si puedo mejorar, ¿no? Entonces, claro, desde ese paradigma, la relación es todo, ¿no? La relación, alumno…iba a decir alumno paciente. La relación alumno o alumna con el profesor lo es todo, para mí por lo menos, lo es todo.

E: Y, ¿cómo fomentas esas relaciones positivas o cercanas con ellos?

P41: Pues simplemente hablando con ellos, y siendo cordial, no sintiéndome un ser superior, como pasa muchas veces. Yo siempre digo cuando empiezan las clases, que, si me preguntas algo y no tengo ni idea, yo te lo voy a decir, “no lo sé”, y, además, siempre les digo, mirar para aquí, ¿no? Y tengo siempre detrás al final de la clase un atril con libros, siempre, todos los años, todos los días. Digo, “si no lo sé, lo buscaremos aquí, y si no abrimos la puerta y buscaremos a alguien que sepa más que nosotros, que hay mucha gente que sabe más que nosotros, entonces, cuando me preguntéis algo que yo no sepa no os voy a responder con cualquier cosa, diré, pues mira, no lo he hecho en mi vida, no tengo ni idea y no pasa absolutamente nada”. Entonces, esa actitud desde el primer día ya rompe barreras vamos, porque o te consideran un inútil y te dicen “pues vaya el que nos va a dar la clase que no sabe nada” o decir, “pues mira, este tío es un poco consciente y sabe decir las cosas”.

E: Y no nos miente, ¿no?

P41: No, no, yo no les voy a mentir, vamos.

**Documento: 3. Ciencias de la Salud\P42 Salud Diseños**

**Peso: 0**

**Posición: 78 - 81**

**Código: 4. Acciones\Relación profesorado-alumnado\4.15. Importancia relación**

E: Muy bien, vamos a profundizar un poco ya que ya lo hemos hablado antes, sobre tus consideraciones entre las relaciones entre profesor-alumno, ¿vale? ¿Es importante para ti la relación que estableces con los estudiantes?

P42: Sí, para mí es una parte fundamental de mi trabajo, una parte de mi labor investigadora en la universidad. Como profesor docente investigador es eso, porque para eso nos contratan en el fondo en la docencia. Aunque luego nos miran por indicadores de investigación, nos contratan para la docencia y una parte central es el alumno. Para mí es central, lo más importante de mi faceta docente, aunque a veces lo miremos con otras variables, mi cliente fundamental es el alumno, igual que en el sistema sanitario yo trabajo por y para el paciente, aquí trabajo por y para el alumno. Entonces, esa parte es clave.

E: Y ¿qué prácticas o estrategias llevas a cabo para fomentar una buena relación con los estudiantes?

P42: Un poco ya lo hemos comentado estos días, pero sí que, para resumir, la disponibilidad y la implicación que como responsable de sus procesos de aprendizaje intento transmitir continuamente, esa es mi estrategia. Ellos son protagonistas y para mí son muy importantes, entonces, cuando yo asumo esas dos cosas, que son tan importantes o más que el decanato, por ejemplo, entienden que son de verdad importantes y participan del proceso, que es lo que busco.

**Documento: 3. Ciencias de la Salud\P43 Salud Diseño**

**Peso: 0**

**Posición: 76 - 79**

**Código: 4. Acciones\Relación profesorado-alumnado\4.15. Importancia relación**

E: En relación, ahora, a la relación entre el profesorado y el alumnado, ¿para ti es importante la relación que se establece entre el alumno y el profesor?

P43: Para mí es fundamental la relación, porque yo pienso que, en general, una relación de confianza y de respeto, por supuesto mutuo. Pero, sobre todo, un espacio libre, en el que se pueda trabajar sin ningún tipo de miedo, se pueda expresar lo que cada uno quiera comentar sobre lo que sea, sobre lo que cada uno quiera. Es decir, un espacio crítico y reflexivo y abierto a las distintas opiniones, yo creo que eso es fundamental. Yo pienso que el profesor tiene una influencia importante en el alumno, si el profesor está desmotivado con una asignatura, el alumno, rara vez va a estar motivado. Si el profesor transmite entusiasmo e ilusión por lo que está enseñando, eso también llega al alumno. Y, entonces, yo creo que la relación profesor-estudiante es fundamental.

E: ¿Qué estrategias llevas a cabo para favorecer la relación con tu alumnado?

P43: Yo las estrategias que sigo son hablar mucho y mostrarme cercana, preguntar, aprenderme los nombres, interpelar usando el nombre y hacer partícipe al alumno de la clase continuamente.

**Documento: 3. Ciencias de la Salud\P44 Salud Diseños**

**Peso: 0**

**Posición: 60 - 63**

**Código: 4. Acciones\Relación profesorado-alumnado\4.15. Importancia relación**

E: En cuanto a la relación profesorado-alumno, ¿para ti es importante la relación que se establece entre el alumno y el profesor?

P44: Es fundamental, porque es la clave, o sea, somos seres relacionales y en base a cómo establecemos las relaciones, eso marca todo lo que hay detrás. Entonces, para mí eso hace un buen proceso de enseñanza-aprendizaje, si no hay una buena relación, pues no hay un buen camino trazado.

E: Y, ¿qué prácticas llevas a cabo para favorecer esa relación con tus alumnos?

P44: Bueno, yo creo que es fundamental el que todos los días cuando llego, lo primero que hago es dar los buenos días, presentarme, y de alguna manera, además, me tengo pactado que el segundo día o tercero, que me tengo que saber todos sus nombres para poder ir llamando a cada uno por su nombre, porque para mí es fundamental, a mí me gusta que me llamen por mi nombre y yo creo que eso ellos también lo reconocen y le dan mucho valor. Que tú el segundo día, por ejemplo, para pedirle aportación para una imagen que tú pongas y le digas “a ver, Pepito Pérez, tú que es lo que consideras que estás viendo ahí”, es un poco que se quedan como ostras, sabe mi nombre, ¿no? Y eso es para mí muy importante, al igual que estos rompe hielos que te digo, que parece una tontería, pero no lo es, esos tres minutos, para mí son vitales en la clase porque de alguna manera los prepara emocionalmente a que puedan desarrollar unas mejores condiciones de poder estar en ese momento y tú recibirles a ellos al cien por cien, que creo que eso también es fundamental.

**Documento: 3. Ciencias de la Salud\P45 Salud Diseños**

**Peso: 0**

**Posición: 60 - 63**

**Código: 4. Acciones\Relación profesorado-alumnado\4.15. Importancia relación**

E: En cuanto a la relación profesor-alumno, ¿para ti es importante?

P45: Bueno, no es que es importante, es que es imprescindible porque mira, a ver, un profesor que no conecta con un grupo…el profesor es un comunicador, si tú no estás conectando con los que te escuchan, tú no vas a obtener nada, absolutamente nada, te pongas como te pongas, digas lo que digas, dibujes como dibujes. Después, puedes dibujar mal, bien, ser más o menos simpática, tener más o menos conocimientos, pero es importante conectar.

E: Y qué estrategias llevas a cabo para favorecer esta relación.

P45: Yo no tengo reglas mágicas, no sé, a mí me enseñaron de una manera determinada de hacer las cosas, a lo mejor después por mi manera de ser, bueno, a lo mejor tengo cierta facilidad para esto, pero es que además el alumno requiere muy poco, o pide muy poco en el sentido de que quiere alguien que se interese por ellos, para empezar, y una persona que llega a clase y no da ni los buenos días, pues poco…una imagen bastante deficiente, a mi modo de ver. Y después, pues que, si hay algún problema, que lo escuche, que a lo mejor es insalvable, que a lo mejor plantean algo y no estás de acuerdo, pero al menos que lo entiendan el porqué de ciertas situaciones, de ciertas cosas que pueden surgir, pero es que no hay más secretos. Al contrario también, ¿eh? Hombre, nosotros somos humanos pasamos por situaciones difíciles, hay veces que no das la clase de tu vida, porque tú tienes detrás una historia personal brutal, te lo digo por experiencia personal, que ha habido momentos que mal no, lo siguiente. Y yo he pedido disculpas, a mí no se me caen los anillos, yo he dado una clase mala y me he dado cuenta y al final les he dicho “lo siento, no es mi mejor día, de todas maneras, cualquier duda cuando leáis lo que sea la resolvemos el próximo día”, y me he ido tan tranquila. Es verdad que al contrario también lo he hecho, que he visto que no me responden, que a los alumnos les importa un pito lo que estoy diciendo porque hay unos cuantos que siempre pido disculpas porque pagan justos por pecadores, y me he ido también de una clase, ¿eh? Y he dado el tema por explicado, o sea, que no soy tan buena. Pero mira, el sentido común es muy importante para todo en la vida.

**Documento: 3. Ciencias de la Salud\P46 Salud Diseño**

**Peso: 0**

**Posición: 108 - 113**

**Código: 4. Acciones\Relación profesorado-alumnado\4.15. Importancia relación**

E: Muy bien. En cuanto a las relaciones entre el profesorado y el alumnado, ¿para ti es importante la relación que se establece entre el alumno y el profesor? ¿Por qué?

P46: Mucho, mucho. Porque yo estoy para eso, son mis alumnos, yo estoy para que ellos aprendan. Yo soy un medio de enseñanza, entonces, para mí, mucho. Es lo que más me importa.

E: ¿La relación con ellos verdad?

P46: Sí, sí. Y yo la relación con el alumnado me la tomo muy en serio, y me afecta.

E: Y, ¿tienes algunas estrategias para tu práctica, digamos, que favorezcan esa relación?

P46: No, solamente atenderlos. Si me preguntan, contestar, estudiar para las clases, para mejorar la docencia, ponerle todos los medios del mundo…

**Documento: 3. Ciencias de la Salud\P46 Salud Diseño**

**Peso: 0**

**Posición: 128 - 129**

**Código: 4. Acciones\Relación profesorado-alumnado\4.15. Importancia relación**

E: Un feed-back previo.

P46: Sí, el alumnado para mí es muy importante. Además, yo mantengo una relación futura. Estos alumnos que me han llamado ahora por teléfono, un antiguo alumno mío que está haciendo el TFG y está en Navarra…Sí, sí, mantengo una buena relación con el alumnado, me gusta.

**Documento: 3. Ciencias de la Salud\P47 Salud Diseños**

**Peso: 0**

**Posición: 54 - 57**

**Código: 4. Acciones\Relación profesorado-alumnado\4.15. Importancia relación**

E: Vamos a hablar ahora de la relación profesor-alumno, ¿para ti es importante esta relación?

P47: Mucho. Porque es la empatía, una parte del aprendizaje. Entonces, si el profesor es antipático, la actitud que tú tienes con la asignatura es mala. Y si a ti los alumnos te resultan antipáticos, también la actitud que llevas a clase es muy negativa y eso se nota. Yo casi en el primero o segundo día de clase soy capaz de saber si ese grupo me va a…claro, es que yo les digo que es también como cuando los papá pájaros llegan al nido y el papá les revuelve el buche, ¿no? entonces, yo cuando más me piquen en el buche, más revujizo cosas que sean buenas. Empatía entre los dos.

E: Y qué cosas utilizas para relacionarte con los alumnos y por qué.

P47: Hombre a mí me gustaría usar más las tutorías como te he dicho. Uso el Blackboard, el correo electrónico, yo siempre les contesto, y luego, saliendo de clase, entrando en clase. Si hay algún grupito me dirijo a ellos para ver si podemos establecer alguna relación. Eso básicamente

**Documento: 3. Ciencias de la Salud\P48 Salud Diseños**

**Peso: 0**

**Posición: 32 - 33**

**Código: 4. Acciones\Relación profesorado-alumnado\4.15. Importancia relación**

E: Vale. Mira como de metodología y de evaluación ya hemos hablado bastante durante la entrevista y también me has hablado quizás, de un poco de puntilla, sobre la relación que tienes con tu alumnado, pues me gustaría preguntarte si crees que la relación que tienes influye en sus aprendizajes.

P48: A ver, yo he tenido experiencias de todo tipo. Experiencias de tener una mala relación y situaciones de tener una buna relación. Yo creo que ahora mi relación es bastante buena, es una relación abierta de explicarles cada cosa que hacemos y que ellos nos den su opinión, que sepan que cuando quieran acceder a mí, que pueden hacerlo, etc., pero eso, no quiere decir que no les exija lo que quieres conseguir con ellos, o sea, que no es una cuestión de decirle que sí a todo, ni mucho menos. Hay un contacto muy de tú a tú y cuando la cosa se tiene que pone exigente, pues no hay problema. Entonces, a veces, el tener esta relación tan directa puede dar lugar a la confusión de que todo vale. Recuerdo un grupo de cuatro o cinco alumnos, y pasaban de todo y no había forma, todo era bronca y demás. Y recuerdo, que a la hora de exponer el póster, fueron los únicos que lo suspendieron, pero porque lo hicieron muy mal y no pidieron ayuda para nada. Luego, cuando tuvieron que recuperarlo, les dije, “veis lo fácil que es trabajar juntos, si no es tan complicado”. Entonces, pues eso, la relación con ellos, en general, es buena y sobre todo, porque es de respeto mutuo.

**Documento: 3. Ciencias de la Salud\P49 Salud Diseños**

**Peso: 0**

**Posición: 62 - 63**

**Código: 4. Acciones\Relación profesorado-alumnado\4.15. Importancia relación**

E: Ya, ya. Vale. Y si hablamos ahora de la relación profesor alumno, bueno, una pregunta un poco obvia, pero, ¿para ti es importante esa relación?

P49: Sí, bueno, porque como he dicho antes, antes no había ninguna relación con los profesores antiguos, no había nada, tenías que llegar, si tenías alguna duda y a ver si podían… Y ahora ellos tienen un acceso muy fácil, ahora con nosotros, bueno, normalmente con todos, puede haber algún profesor especial que les ponga pegas, que ponga distancias entre ellos, pero a mí no me gusta que haya distancias, de hecho, no nos vamos a tomar cervezas todos los días, pero que podría ser, o sea que… Y, al final de la carrera, por ejemplo, como son seis años, pues creas un poco una relación casi, casi, de amistad ya, porque claro, han sido muchos años y ellos lo agradecen y vamos, yo creo que tiene que ser así. Ellos tienen acceso a nosotros y es muy fácil.

**Documento: 3. Ciencias de la Salud\P50 Salud Diseños**

**Peso: 0**

**Posición: 104 - 107**

**Código: 4. Acciones\Relación profesorado-alumnado\4.15. Importancia relación**

E: Muy bien. En cuanto a la grabación de profesor-alumno, ¿para ti es importante esa relación que se establece entre tú como profesor y los estudiantes?

P.50: Hombre, sería ideal que fuese una relación fluida, pero incluso si al alumno le caes mal y a ti te cae mal el alumno, porque somos humanos, ¿no? Pero el alumno cumple su función, yo cumplo mi función, debería ir perfectamente. Lo ideal sería que te respetase y que tú al alumno lo apreciases, pero esto no es un mundo ideal.

E: Y, ¿haces algo para fomentar esa buena relación con los estudiantes?

P.50: Sí, o sea, es difícil llegar al equilibrio entre que el alumno te respete, pero que no te vea como un guardia civil o como una autoridad que cause hasta miedo, ¿no? Sino que vean que tú respetas al alumno y que el alumno te respete a ti. Entonces, conseguir ese equilibrio necesita años, pero yo creo que, más o menos, el alumno, yo tengo mi nivel de autoridad y de respeto, pero saben que me pueden hacer cualquier pregunta perfectamente y no creo que estén en una tensión de una autoridad y una persona que depende de una autoridad. Yo creo que ese equilibrio siempre se podrá mejorar y en algunas clases será diferente a otras porque ya depende del alumnado. Tengo clases en inglés de 15 alumnos y clases en español de 160, no es lo mismo, ¿no? Pero yo intento que “oye, soy tu profesor, no lo olvides”, pero fuera de clase si terminan las clases y yo me tengo que tomar algo con un alumno, eso sí lo hago. Pero que el alumno vea que soy accesible, que se puede dirigir a mí, que soy respetuoso… Pero eso tampoco significa que esto sea un cachondeo y que me escriban un correo y me digan “mira qué pasa”, o sea, un equilibrio. Y eso tú no llegas y dices allí “quiero esto”, sino que predicas con el ejemplo, claro.

**Documento: 3. Ciencias de la Salud\P51 Salud Diseños**

**Peso: 0**

**Posición: 56 - 61**

**Código: 4. Acciones\Relación profesorado-alumnado\4.15. Importancia relación**

E: Vale. Y acerca de la relación profesorado-alumnado, ¿para usted es importante la relación que se establece entre ambos?

P51: Sí.

E: ¿Por qué?

P51: Porque considero que me van a facilitar la comunicación, que me van a contar más cosas, que van a verme más accesible y facilitadora y que, por tanto, se implicarán más en la clase.

E: Y qué prácticas o estrategias suele llevar a cabo para fomentar la participación con su alumnado.

P51: Pues, normalmente suelo preguntarles al principio de la clase que cómo están, cómo van… Me intereso por ellos.

**Documento: 3. Ciencias de la Salud\P52 Salud Diseños**

**Peso: 0**

**Posición: 60 - 63**

**Código: 4. Acciones\Relación profesorado-alumnado\4.15. Importancia relación**

E: Claro. Y con respecto a la relación profesorado-alumnado, ¿consideras que es importante?

P52: Sí, absolutamente. Les tienes que caer bien, si les caes mal, no van a aprender. O sea, el prejuicio puede poder más. Entonces, tienes que hacer todo lo posible por caerles bien, siempre que eso no suponga…o sea, yo no soy su amiga, eso lo dejo muy claro desde el primer momento, yo soy su profesora. Pero la relación puede ser agradable y reforzante, no hace falta que sea una relación para sufrir, ¿sabes?

E: Sí, sí. Y qué prácticas utilizas para favorecer esa relación.

P52: Yo uso muchísimo el humor. Me río de mí misma, de ellos… El humor está presente en toda mi docencia porque yo creo que es básico. Que no se lo tomen con angustia o se sientan presionados, sino que aprender es divertido, tiene que serlo para ellos.

**Documento: 3. Ciencias de la Salud\P53 Salud Diseños**

**Peso: 0**

**Posición: 46 - 47**

**Código: 4. Acciones\Relación profesorado-alumnado\4.15. Importancia relación**

E: Y, esto también más o menos lo deduzco por lo que se ha ido comentando a lo largo del discurso de que la relación profesorado-alumnado la considera muy importante.

P53: Sí, claro, porque, vamos a ver, si simplemente son receptores pasivos de unos mensajes, está bien, pero es bastante incompleto, es mejor cuando participan de manera activa y…vamos a ver, no hace falta ir a extremos, no les voy a montar una obra de teatro cada vez que tenga que explicarles un tema, pero sí hacerles partícipes, darles material que pueda resultar atractivo, sin olvidarnos de los libros y presentaciones y tal, pero a lo mejor sí que podemos acudir a cierto material que les resulta atractivo, especialmente en una generación que está tan en contacto con el medio audiovisual y que, como consecuencia de esto, es una generación que tiene mucha menos atención sostenida en una tarea. Entonces, sin alimentar la superficialidad de estar siempre conectados a mensajes cortos, sin alimentar eso, sí que tratas un poco de adaptarte a lo que más les gusta.

**Documento: 3. Ciencias de la Salud\P54 Salud Diseños**

**Peso: 0**

**Posición: 54 - 57**

**Código: 4. Acciones\Relación profesorado-alumnado\4.15. Importancia relación**

E: Vale. Y en cuanto a la relación profesorado-alumnado, ¿para ti es importante esta relación?

P54: Yo creo que es fundamental para motivarlos y para que tengas feedback de si les va bien o no, de si les cuesta o no les cuesta, yo creo que sí, yo creo que es fundamental.

E: Y qué prácticas llevas a cabo para favorecer la relación con tu alumnado.

P54: Pues, digamos, a nivel formal, creo que ninguna, solamente ser plano, ser directo, estar receptivo, abierto a que vengan a cualquier hora al despacho, mientras yo esté. Digamos, no poner trabas a…incluso a la comunicación entre ellos, quiero decir, es una…lo que pasa que creo que es parte de mi manera de ser, que creo que ayuda a la motivación un poco.

**Documento: 4. Ciencias Sociales y Jurídicas\P 56 CCSS Creencias**

**Peso: 0**

**Posición: 38 - 41**

**Código: 4. Acciones\Relación profesorado-alumnado\4.15. Importancia relación**

E: Sí te veo ahí fotos de…

P. 56: Sí, son de los niños de alumnas que me decían que tenían hijos y les digo “tú te lo traes a clase”. Entonces me dibujaban los niños en clase con todas sus cosas. Les digo “¿tú vas a perderte a clase por tener un niño? Ya te lo puedes estar trayendo.”

E: Ah, muy bien.

P. 56: Y eso, yo creo que nosotros no somos los amigos de los alumnos en absoluto, tampoco tienes que adoptar ese rol, pero tú tienes que intentar hacer todo lo posible para mejorarles la vida en todo lo necesario, y sobre todo en la labor que tiene que hacer el profesor desde el minuto cero es orientar bien en las salidas profesionales, no porque tengas que decirle qué es lo que van a hacer o porque ellos tengan que tener claro qué es lo que quieren hacer, sino que ellos tienen que tener claro qué es lo que no quieren hacer cuando acaben la carrera, y en función de eso decidir.

**Documento: 4. Ciencias Sociales y Jurídicas\P 56 CSS Diseños**

**Peso: 0**

**Posición: 40 - 43**

**Código: 4. Acciones\Relación profesorado-alumnado\4.15. Importancia relación**

E: Vale. ¿Para ti es importante la relación profesor-alumno?

P. 56: Para mí es importante, pero reitero, yo no soy el colega de ellos, a mí no me pueden tratar como un colega ni yo me llamo “oye”, porque así no pueden hablarle a un juez ni a un abogado dentro de dos años cuando estén en la calle. Entonces, ellos tienen que aguantar una formalidad como la aguanto yo. Aunque después tenga alumnos que hoy en día son mis amigos, pero todo desde el punto de vista del respeto que yo les tengo y que ellos me tienen que tener a mí.

E: Vale. Y para comunicarte con ellos, qué medios utilizas.

P. 56: O bien en clase o bien por correo electrónico.

**Documento: 4. Ciencias Sociales y Jurídicas\P 57 CSS Diseños**

**Peso: 0**

**Posición: 34 - 39**

**Código: 4. Acciones\Relación profesorado-alumnado\4.15. Importancia relación**

E: En relación con la relación entre el profesor y el estudiante, ¿tú crees que esta relación es importante?

P. 57: Yo creo que sí, que es fundamental.

E: ¿Tú qué medios utilizas para comunicarte con el alumnado?

P. 57: Habitualmente, directamente cuando vienen a clase, por correo electrónico, por la plataforma y en uno de los casos que tuve, por contacto telefónico.

E: ¿Tú cómo valoras tu relación con los estudiantes?

P. 57: Yo creo que es buena, no digo que sea excepcional, pero sí es buena.

**Documento: 4. Ciencias Sociales y Jurídicas\P 58 CSS Diseños**

**Peso: 0**

**Posición: 70 - 75**

**Código: 4. Acciones\Relación profesorado-alumnado\4.15. Importancia relación**

E: Y, ahora sobre la relación entre el profesorado y el alumnado, ¿para ti es importante la relación que se establece entre ambos?

P. 58: Sí.

E: Por qué crees que es tan importante.

P. 58: Porque es una relación que no debe ser fría, sino que debe ser cercana en la medida de lo posible y para si quieres fomentar que el estudiante participe, esté motivado… Entonces tienes que tener una relación adecuada, cercana dentro del respeto, por supuesto, ¿no? Simplemente una relación correcta, cercana, empática… Por parte del profesorado, pero vamos, por parte del estudiante, insisto, tiene que poner de su parte, pero tiene que ser una relación que sea sana, ¿no? que el estudiante vea que estás ahí, que estás dispuesto a ayudarlo… Dentro de la razonabilidad. Hay cosas que no, que piden los estudiantes que no son razonables, y es que no, pero para otras cuestiones, es importante que el profesor esté ahí, ¿no?

E: Y qué prácticas o estrategias llevas a cabo para favorecer la relación con los estudiantes.

P. 58: Pues yo intento hablar con ellos, yo no soy una persona que vaya a la clase a soltar el rollo e irme, ¿no? Sino que llego, intento hablar con ellos, intento conocerlos… Aprenderte el nombre de tantísimos estudiantes es imposible, pero sí que intento aprenderme sus nombres, de algunos al menos, preguntarles para que participen, intento también hablarles de otras cosas de la vida también que son importantes y no solo del contenido de la asignatura, también de la universidad en general, que no solamente es ese aula y ya está, sino que es mucho más… Entonces, todo ese tipo de cosas intento transmitirlas.

**Documento: 4. Ciencias Sociales y Jurídicas\P 59 CSS Creencias**

**Peso: 0**

**Posición: 31 - 31**

**Código: 4. Acciones\Relación profesorado-alumnado\4.15. Importancia relación**

Yo trato de memorizar el nombre de mis alumnos y alumnas, yo les llamo por su nombre y cuando veo que alguien, pues viene poco a clase o da mucho por culo en clase y perdón la expresión, o veo que tiene alguna rareza… Yo tenía un alumno que pensaba que me iba a pegar, yo decía “este un día en la clase…”

**Documento: 4. Ciencias Sociales y Jurídicas\P 59 CSS Creencias**

**Peso: 0**

**Posición: 37 - 37**

**Código: 4. Acciones\Relación profesorado-alumnado\4.15. Importancia relación**

En ese equilibrio que ti también sabrás que hay que mantener entre “no soy tu profesor que está en el altar, pero tampoco soy tu colega”, ¿no? Porque es que esa línea es muy delgada, de cómo te acercas tú, pero manteniendo la posición de profesor-alumno. Pero bueno, yo no me meto tanto en esos berenjenalitos, doy un pasito atrás creo, a lo que hacen otros compañeros míos, ¿no?

**Documento: 4. Ciencias Sociales y Jurídicas\P 60 CSS Diseños**

**Peso: 0**

**Posición: 56 - 59**

**Código: 4. Acciones\Relación profesorado-alumnado\4.15. Importancia relación**

E: Vamos a hablar ahora de la relación entre el profesor y el alumno, para ti, ¿es importante la relación que se establece entre ambos? Y por qué.

P. 60: Pues mira, para mí es muy importante la relación que se establece entre el profesorado y el alumnado, a veces, creo que hay que tener una línea divisoria porque ellos todavía creo que tienen que madurar en el sentido de las relaciones sociales en general, pero yo muchas veces traspaso esa línea e intento que no, pero la vuelvo a traspasar ¿Por qué? Porque creo que es básico para ellos que vean en ti, independientemente, de que tú seas su profesor y ellos tus alumnos, pero es que no creo que de los alumnos al profesor tenga que haber más respeto que del profesor al alumno o viceversa. Creo que hay que difuminar un poco esas diferencias tan antiguas y que no se te vaya de las manos por supuesto, que no estés en un grupo de WhatsApp con tus alumnos, ¿vale? Que no se tiene que llegar a eso, pero sí que tengan confianza, libertad, que se puedan expresar, que no tengan miedo, que no se avergüencen si dicen cualquier tontería o algo que no esté vinculado con la materia, que no… O sea, que estén tranquilos en clase y con confianza, con serenidad y relajados a la hora de aprender. Yo creo que eso es lo que más hago con ellos vamos.

E: ¿Y pones en práctica alguna estrategia para favorecer esa relación con tu alumnado?

P. 60: Más allá de decírselo y de demostrarles a ellos la misma confianza que les pueda yo transmitir, no.

**Documento: 4. Ciencias Sociales y Jurídicas\P 61 CCSS Creencias**

**Peso: 0**

**Posición: 120 - 131**

**Código: 4. Acciones\Relación profesorado-alumnado\4.15. Importancia relación**

E: ¿Tienes algún problema de disciplina en tu clase? Porque yo veo que tú tienes el modelo de lo que todos queremos… Muchas veces yo, personalmente en mi aula, me encuentro a veces como con algún problema de disciplina o con los móviles, con entrar y salir de la clase… ¿Cómo manejas todo eso tú? Porque es difícil el equilibrio.

P. 61: Sí, sí. Hay que trabajar el equilibrio porque te tienes que mostrar cercano, pero te tienes que mostrar también…es muy difícil. Gracias también a que paso lista y que tengo identificados los focos, pues a continuación, muchas veces, cuando he visto algún foco, le mandaba un correo y les digo “me gustaría hablar con usted”. Les trato de usted para evitar alguna barrera, porque normalmente trato de hablarles de tú, pero entonces sí que pongo alguna barrera “me gustaría hablar con usted”. En esos casos me pongo muy estricto, vienen aquí y entonces los reincluyo. Es difícil identificar los focos, pero es necesario abordarlos enseguida, no dejarlos pasar y más o menos así me ha funcionado.

E: Cuando, por ejemplo, ves un móvil en clase…

P. 61: Sí, móviles enseguida…

E: No toleras. No sé si te pasa a ti, pero a mí, alguna vez, a pesar de haber dicho muchas veces, encuentro que están por debajo de la mesa enviando un mensaje o mirando no sé qué.

P. 61: Alguna vez veo los móviles y pienso que están cogiendo las transparencias, pero tampoco…

E: Ah bueno.

P. 61: Pero siempre…otra cosa que no he dicho, una táctica que sigo es saberme los nombres desde muy pronto, llamarles por su nombre. Por lo que, si veo alguna persona que está dispersa o hablando, digo “a ver Juan, tal, tú qué opinas, ¿estás conmigo?”, o cuando los veo dispersos “tal, ¿estás siguiendo?”. Pues sí que…pero el saberse el nombre de cada uno, también hace muchísimo en cuanto a que vean que por parte del profesor hay más que solo la docencia. Pero yo creo que abordarlos muy puntualmente los focos y que te vean que no dejas pasar esos casos, el llamar…

E: Al orden.

P. 61: Decir “oye me gustaría que vinieras a hablar porque no veo…y en el caso de que no cambies, pues me gustaría que…”. En esos casos sí que soy…

E: Más inquisitivo, sí.

P. 61: Sí, sí. Ahí cambias radicalmente. Desde tratarles de usted hasta ponerte muy… Yo recuerdo que, en los primeros años, decía que en caso de volver a ocurrir que no siga con mi asignatura y que no le voy a…” porque yo valoro eso más que el examen y que no va a aprobar mi asignatura. Al final han cambiado muchísimo, alumnos así, que eran muy conflictivos, de repente son muy brillantes, incluso después de ser alumnos me han agradecido muchísimo. Es decir, abordarlos muy puntualmente, tener identificados siempre los focos y luego también de lo que me he dado cuenta, al margen de que pase lista, yo comento que no es obligatorio asistir a mi clase, que sí que es verdad que a quien asiste le es mucho más sencillo sacar la asignatura. No porque yo...estamos haciendo lo mismo que en el examen, sino que el que venga realmente, viene por interés y; sin embargo, consigo tasas de asistencia enormes.

**Documento: 4. Ciencias Sociales y Jurídicas\P 62 CCSS Creencias**

**Peso: 0**

**Posición: 66 - 67**

**Código: 4. Acciones\Relación profesorado-alumnado\4.15. Importancia relación**

E: P. 62, ¿qué actitudes personales crees tú que ayudaron? Tuyas, personales tuyas.

P. 62: Sí. Pues yo creo que una actitud de cercanía. Yo creo que eso le ayudó, o sea, me sentía como una persona dispuesta a apoyarle en lo que hiciera falta siempre y cuando tuviese como compensación ver que respondía positivamente. Eso también se lo dije “mira, si yo estoy aquí dejándome la piel para que las cosas te vayan sobre ruedas, que no tengas ningún problema con la asignatura, si empiezas en plan pasota, paso yo más que tú, que yo pasar también sé”. Y entonces, yo creo que se creó esa especie de magia de “tú estás apostando por mí y no voy a defraudarte y aquí estoy al cien por cien y vamos a terminar teniendo una relación más allá casi de profesor-alumno, más casi de amistad”.

**Documento: 4. Ciencias Sociales y Jurídicas\P 62 CCSS Creencias**

**Peso: 0**

**Posición: 68 - 70**

**Código: 4. Acciones\Relación profesorado-alumnado\4.15. Importancia relación**

E: ¿Sigues manteniendo contacto con él?

P. 62: Al principio sí, me escribió un par de veces, pero luego ya no. Y me contó que le iba bien, tenía miedo al ámbito profesional a enfrentarse al mundo de las empresas y cómo podría ser su futuro y le dije que lo desconocía cómo podría ser porque es inevitable que al final, a veces te dé más o menos corte. Pero yo le decía que la preparación la tenía y las ganas también y hay gente que tiene la preparación y no tiene las ganas y hay gente que tiene las ganas y no tiene la preparación. Entonces, está bastante equilibrada su situación. Parecía que podía ser una persona con posibilidades. También es verdad que tenía que medicarse. Eso lo tenía clarísimo. Si dejaba la medicación es que se le notaba, ¿sabes lo que te digo? Es que se le notaba.

E: Claro.

**Documento: 4. Ciencias Sociales y Jurídicas\P 62 CCSS Creencias**

**Peso: 0**

**Posición: 126 - 131**

**Código: 4. Acciones\Relación profesorado-alumnado\4.15. Importancia relación**

E: ¿Conoces a todos los alumnos? Ya me imagino, claro.

P. 62: Sí, bastante. Suelo aprenderme los nombres porque me parece que es una manera también de hacer muchísimo más cercana la situación de tarima-no-tarima.

E: Tú te bajas de la tarima, ya veo.

P. 62: Yo siempre. Le digo “Lucas” y Lucas dice “ah, sabe cómo me llamo” y entonces es “si ella sabe cómo me llamo, yo debería estar atento”.

E: Claro, claro.

P. 62: Sí, entonces es fundamental. Para que te hagas una idea, yo les paso asistencia con el Administ, pero les paso asistencia…mira, es una tontería. Pero tampoco las puedo tener preparadas porque depende de la sesión y de cómo se han portado. Mira la clase de hoy es esta, entonces yo les paso todas las sesiones.

**Documento: 4. Ciencias Sociales y Jurídicas\P 62 CSS Diseños**

**Peso: 0**

**Posición: 82 - 85**

**Código: 4. Acciones\Relación profesorado-alumnado\4.15. Importancia relación**

E: Claro, qué interesante. Y acerca de la relación profesorado-alumnado, ¿crees que puede influir en su aprendizaje?

P. 62: Estoy absolutamente convencida.

E: Por qué.

P. 62: Si ellos te ven como una persona que les va apoyar, y no solo les va a valorar, entonces su actitud es muchísimo más receptiva y colaborativa.

**Documento: 4. Ciencias Sociales y Jurídicas\P 63 CCSS Creencias**

**Peso: 0**

**Posición: 31 - 31**

**Código: 4. Acciones\Relación profesorado-alumnado\4.15. Importancia relación**

O sea, intento estar lo más cerca de ellos porque creo que es la única forma en la que realmente les puedo ayudar a que aprendan y a que aprendan no sé, no solamente los contenidos, que al fin pues es el objetivo, sino que, no sé, que vean que el mundo laboral es complicado pero que al final son todos personas. Las empresas son personas, la sociedad son personas. Entonces, yo creo que es lo que trato, el ser lo más próximo a ellos.

**Documento: 4. Ciencias Sociales y Jurídicas\P 63 CCSS Diseños**

**Peso: 0**

**Posición: 44 - 45**

**Código: 4. Acciones\Relación profesorado-alumnado\4.15. Importancia relación**

E: Nosotros lo que vemos P. 63 es que tú fomentas muy bien las relaciones con los estudiantes. Entonces, a parte de las prácticas que haces al inicio de tus clases para fomentar la relación con el alumnado, pues ¿haces algo para fomentar la relación con tus alumnos?

P. 63: Hombre, yo creo que es muy importante el hecho de que tú les llame por su nombre. Además, al saber los nombres, pues creo que ellos ponen más interés.

**Documento: 4. Ciencias Sociales y Jurídicas\P 64 CCSS Creencias**

**Peso: 0**

**Posición: 119 - 119**

**Código: 4. Acciones\Relación profesorado-alumnado\4.15. Importancia relación**

No, pues yo creo que ahí la forma de eso es buen material... Hombre, yo creo que la cercanía es importante. Yo creo que por ir de más estirado los estudiantes no mejoran necesariamente. Entonces, me parece que yo soy... Procuro ser cercano. Evidentemente, si alguien se pasa de cercano dices “ay muchachín, muchachín, mucho cuidado, ¿vale?” Pero normalmente me pasa muy poco. Normalmente yo creo que la gente no se pasa en general. Entonces, yo creo que esa combinación... Y también es importante, creo yo, que te sientas seguro con lo que estás explicando. Es decir, que tú controles la materia realmente. No se trata de hacerlo mono, sino de hombre, también saber lo que... Yo creo que no hay peor docente cuando uno no conoce bien su materia. No sé si te he ayudado, pero bueno.

**Documento: 4. Ciencias Sociales y Jurídicas\P 64 CCSS Diseños**

**Peso: 0**

**Posición: 78 - 79**

**Código: 4. Acciones\Relación profesorado-alumnado\4.15. Importancia relación**

E: Y, en cuanto a tu relación con los alumnos, me gustaría preguntarte si para ti es importante esa relación.

P. 64: Hombre, yo creo que si es importante, supongo que sí, no lo sé. Yo desconozco qué cosas les estimulan más a estudiar o a aprender, no lo sé. Pero vamos, yo siempre procuro ser bastante cercano a los alumnos y no sé, y sin faltarles al respeto ni tomarme confianzas que no proceden y viceversa, pero no sé, dándoles pie a…no sé, a que haya una… Algunos me llaman de usted, “oye, que tengo una cosa… ¿mañana por la tarde vas a estar?”, no sé, eso que podría ser normal cuando yo empezaba porque nos llevábamos muy poquitos años, pues, evidentemente, que con el paso de los años que se mantenga esa relación, pues es una forma de…también es cierto que eso ahora ha cambiado. Es raro que…les cuesta hablar de usted, por ejemplo. Incluso en alguna empresa ha supuesto un problema para ellos, sobre todo en el caso de la gente que atiende al público, y les cuesta muchísimo, o sea, no saben hablar dirigiéndose de usted. Me acuerdo una vez un agente de hoteles, que decía que tenían que hacerles un entrenamiento porque tú no sabes…tú tendrás que hablarle de usted a la gente e incluso resistirte como gato panza arriba. Y el problema está en que no sabían conjugar, a veces, de forma adecuada, es decir, era “usted quieres…”, no, no. Entonces claro, se cambiaban, tenían que hacer un pequeño training de un día o dos y decir, “vamos a ver, cómo…”, es decir, que en eso te adaptas. Yo no tengo mayor problema en relacionarme con ellos y ser cercano…un profe que vaya de estirado ni nada de eso, lo importante, lo que cuenta es lo que transmites y ya está.

**Documento: 4. Ciencias Sociales y Jurídicas\P 65 CCSS Diseños**

**Peso: 0**

**Posición: 49 - 50**

**Código: 4. Acciones\Relación profesorado-alumnado\4.15. Importancia relación**

E: Y en cuanto a la relación profesor-alumno, ya me has comentado que para ti es muy importante, pero qué estrategias llevas a cabo para desarrollar esa relación que tenéis.

P. 65: Buen humor. Ahí no entra un profesor con un palo en el culo, ahí entra un comercial que entra de la calle, pero que es un profesional. Entonces, el primer día es lo que les digo “esto es un consejo de administración y cada vez que venimos a clase esto es una sesión de trabajo. ¿Os imagináis a una persona ahí mascando chicle?, ¿verdad que no?, ¿os imagináis que van a revisar las cuentas del año y no han mirado las cuentas?, ¿os imagináis bostezándole en la cara al presidente de la compañía?, ¿verdad que no?”. Entonces ya se ponen en situación. Para mí es muy importante que entiendan la faceta del profesor, pero también la suya.

**Documento: 4. Ciencias Sociales y Jurídicas\P 66 CCSS Diseños**

**Peso: 0**

**Posición: 78 - 83**

**Código: 4. Acciones\Relación profesorado-alumnado\4.15. Importancia relación**

E: ¿Para ti es importante la relación que se establece entre alumnado y profesorado?

P. 66: Sí, aunque me gustaría que hubiera más relación. Pero estamos en un sistema en el que los estudiantes que están estudiando nuestra especialidad, pues no están motivados porque para algunos de ellos fue la tercera o la cuarta opción que pusieron en sus preferencias y a lo mejor por eso vienen a clase y atienden y no quieren tener una buena relación con el profesor.

E: ¿Por qué crees que es importante mantener esta relación?

P. 66: Bueno es una relación única y exclusivamente académica. La relación es importante porque es una de las claves para el aprendizaje es equivocarse, pero cuando te equivocas, pues te tiene que decir en qué te has equivocado y cómo puedes mejorar. Éste es el primer aspecto. El segundo aspecto, es porque necesitas distintos puntos de vista, muchas veces sobre un tema determinado tú tienes una opinión y siempre te interesa saber la opinión que puede tener otra persona. En un problema de matemáticas relacionado con la vida real, pues puede haber muchas soluciones en función de las diferentes perspectiva. Entonces, el intercambiar puntos de vista y discutir el problema con otras personas pues te puede dar luz sobre un determinado aspecto que tú no habías visto.

E: ¿Qué prácticas o estrategias llevas a cabo para fomentar la relación con tu alumnado?

P. 66: Es que es muy simple lo que yo hago, es decir yo planteo una cuestión, dejen que se expresen y escucho. Ya n hago más nada, además, trato de no contestar, sino que dejo que sean los propios compañeros quien les conteste. Entonces, procuro que lo estudiantes hablen, opinen y yo trato de dirigir el debate y procuro no ser yo quien conteste, sino que sean sus compañeros quienes le contesten.

**Documento: 4. Ciencias Sociales y Jurídicas\P 67 CCSS Diseños**

**Peso: 0**

**Posición: 40 - 43**

**Código: 4. Acciones\Relación profesorado-alumnado\4.15. Importancia relación**

E: ¿Y es importante para ti la relación con el alumnado?

P. 67: Sí, es importantísima, básica. Si no hay buena relación, el estudiante se desincentiva mucho a la hora de involucrarse en la asignatura, por eso la empatía es fundamental.

E: ¿Tú qué medios utilizas para comunicarte con los estudiantes?

P. 67: A ver, utilizo el aula virtual, la mensajería del aula virtual y la comunicación oral en los pasillos, en el despacho o cuando nos vemos en el aula. Yo soy muy accesible como te he dicho y me suelen abordar en cualquier lado, incluso en la cafetería, para cerrar tutorías. Obviamente, cuando es algo oficial, como notas, comunicados, pues todo se centraliza y canaliza a través del aula virtual. Dudas individuales, aspectos sobre la asignatura o las pruebas, en persona bien en las clases o en el despacho en tutoría.

**Documento: 4. Ciencias Sociales y Jurídicas\P 68 CCSS Diseños**

**Peso: 0**

**Posición: 58 - 59**

**Código: 4. Acciones\Relación profesorado-alumnado\4.15. Importancia relación**

E: Claro. Y, ahora hablando de la relación tuya con tus alumnos…la pregunta es un poco obvia, pero bueno, ¿es importante para ti la relación que tienes con tus alumnos?

P. 68: Hombre, por supuesto, a ver, aquí hay varias estrategias. A la hora de enseñar, creo que una es el terror, porque he tenido profesores que infundían terror y eso te presiona a esforzarte para superar la asignatura y luego es como un teatro al final, porque el profesor no tiene nada en contra tuya, ¿no? Pero sí que parecía que funcionaban infundiendo este terror, ¿no? Para incentivar al estudiante a esforzarse y aprender. Y luego te llevas genial con ese profesor, ¿no? Pero era como un teatro. Y luego, otra perspectiva es, pues intentar ser más amable, intentar incentivarlos por otro lado, mostrándoles la importancia de la asignatura, siendo tú más compañero o acompañante en el proceso de aprendizaje que imponer, ¿no? Entonces, esta segunda opción es la que yo intento, que ellos me vean como un recurso más que les acompaña para ayudarles a entender la asignatura. Ellos podrían coger el libro por internet, pero yo lo he hecho antes para luego proveerles de lo que creo que es más importante o lo que puede ayudarles a entenderlo. Yo tenía un compañero que decía que el docente era un facilitador de conocimiento, es decir, tú te lo estudias antes, lo comprendes, has tenido experiencia laboral al respecto, y luego tú en el aula, si enseñas lo que trabajas, lo que has investigado antes, pues tu tarea es darles a conocer esa disciplina y facilitarles el aprendizaje, resolviéndoles dudas, guiándoles en los problemas a los que se pueden enfrentar… Entonces, yo creo que eso es lo que intento, pero repito, no sé si lo consigo, porque, por supuesto, voy a tener estudiantes que me odien en clase, por supuesto y no pocos, pero yo intento que esto no ocurra, intento eso, que se vayan sin dudas, que entiendan las cosas y doy mis facilidades en el sentido de eso, de “vale, las clases son de dos horas, no lo entendemos, pero venga, vamos a tutorías, vamos a verlo o cuelgo esta solución y lo repasáis y si tenéis dudas me lo decís, o cuelgo algo y por email, cualquier duda me preguntáis…”, intento ser accesible y facilitar el conocimiento. Entonces, bueno, yo creo que algunos eso lo valoran y esa es la idea.

**Documento: 4. Ciencias Sociales y Jurídicas\P 69 CCSS Diseños**

**Peso: 0**

**Posición: 78 - 83**

**Código: 4. Acciones\Relación profesorado-alumnado\4.15. Importancia relación**

E: Cada una aporta algo. Y en cuanto a la relación profesorado-alumnado, ¿para ti es importante esta relación?

P. 69: No mucho.

E: Por qué.

P. 69: Porque creo que no aporta demasiado a lo que necesitan y en el proceso de aprendizaje, no.

E: Entonces, imagino que no llevas a cabo ningún tipo de estrategia para fomentar la relación con el alumnado…

P. 69: No, nada.

**Documento: 4. Ciencias Sociales y Jurídicas\P 70 CCSS Diseños**

**Peso: 0**

**Posición: 26 - 29**

**Código: 4. Acciones\Relación profesorado-alumnado\4.15. Importancia relación**

E: Con respecto a la relación del profesor con los alumnos, ¿tú qué medios utilizas para comunicarte con los alumnos?

P. 70: Además, de la clase, pues el correo electrónico y la plataforma.

E: Tú relación con los estudiantes cómo la valoras.

P. 70: Creo que bien. No he tenido ningún problema con algún alumno y si lo he tenido, pues el alumno no me lo ha dicho. Seguro que habrá un alumno por ahí que diga que lo he tratado mal. Mira, pues con este alumno en concreto tuve un malentendido un día que no me acuerdo…pero bueno, tuvo la cosa de verbalizarlo y planteármelo y lo aclaramos. Fue porque le hice una broma en clase y a él no le sentó muy bien. Pero vamos que lo aclaramos y punto. Aunque ya te digo que esto depende del grado de madurez del alumno, porque cuanto más maduro eres, menos te cuesta verbalizar los problemas y hablarlos.

**Documento: 4. Ciencias Sociales y Jurídicas\P 71 CCSS Diseños**

**Peso: 0**

**Posición: 82 - 93**

**Código: 4. Acciones\Relación profesorado-alumnado\4.15. Importancia relación**

E: Y, ¿para ti es importante la relación profesor-alumno?, ¿crees que influye en el aprendizaje?

P. 71: Sí, el cómo los trates influye muchísimo en el aprendizaje.

E: ¿Tú cómo te definirías?

P. 71: Yo con cercanía.

E: Te consideras una persona cercana, ¿no?

P. 71: Cercanía. De vez en cuando me estudio alguna ficha, las tengo aquí, y voy viendo quienes son y ese día, pues le toca al que me haya estudiado la ficha, y les sorprende mucho, porque cuando tú les digas “Menganito”, eso les sorprende mucho, porque dicen “ostras, que sabe mi nombre”, entonces, en cada clase me tocan tres o cuatro alumnos para preguntarles directamente.

E: Esa es una de tus estrategias que no fallan para fomentar la relación, ¿no?

P. 71: Eso lo hacía un catedrático que tenía yo en primero de carrera de derecho, que es un personaje, Gil Arévalo, y yo no sé seguro, yo creo que era capaz de aprendérselos, a mí me conocía seguro, a mí me conocía de antes de empezar la carrera, pero yo no sé si es que era capaz el tío de aprenderse las caras de todo el mundo, que me lo imagino que es posible, y nos sorprendía mucho, porque estábamos así y tal, y si te veía distraído “¡Fulanito! ¿Qué es lo que estábamos haciendo?”.

E: Y, te lo voy a relacionar con otra, porque, ¿tú piensas que al tener esa relación con el alumnado eso hace que estén más motivados?

P. 71: Sí. Yo creo que les motiva muchísimo porque les implico, es que yo les implico.

E: Les haces partícipes, ¿no?

P. 71: Sí. Yo lo que quiero es que piensen “tengo que aprobar porque si no Jesús va a estar triste”, “a Jesús le va a sentar fatal si suspendo”, y yo les atraigo para eso, para que ellos estén en mi lado y piensen “no, no, tengo que aprobar, el profesor me ha preparado para que apruebe”.

**Documento: 4. Ciencias Sociales y Jurídicas\P 72 CCSS Diseños**

**Peso: 0**

**Posición: 110 - 119**

**Código: 4. Acciones\Relación profesorado-alumnado\4.15. Importancia relación**

E: Y, ¿para ti es importante la relación entre el alumnado y el profesorado?

P. 72: Sí.

E: Por qué.

P. 72: Es importante. Es verdad que hay profesores que solo dan una asignatura y yo, por ejemplo, en relaciones laborales, las clases son bastante numerosas. Sobre todo, en los primeros cursos son muy numerosas. Esta no porque es en tercero y yo tengo 45-50 alumnos, que no es mucho, pero en sistemas, que es en segundo, tengo 80 alumnos en un grupo y no he contado ni los de tarde porque no me quiero todavía agobiar. Entonces, no es lo mismo, no es lo mismo que tú puedas plantear una cuestión u otra. Es que depende, por ejemplo, en esta que te comento que es de segundo, todas las cuestiones estas prácticas son en grupo, hacen un trabajo de un modelo de un país de relaciones laborales en grupo, en grupos de cinco personas, sino es que a mí no me da tiempo a corregir, no puedo.

E: Y, qué cosas puedes hacer en el aula para facilitar esa comunicación entre el alumnado y el profesorado.

P. 72: Verás, yo intento…yo, para facilitar la comunicación, yo soy seria, pero más bien cercana. Entonces, en ese caso, sí es cierto que bueno, que intento poner ejemplos, algunos míos, es decir, hoy estábamos viendo una cosa en clase y yo he puesto mi ejemplo, “mira, pues a mí me pasó esto y al final, pues acepté este trabajo”. Entonces, intentar que ellos puedan también…que, aunque se equivoquen, intentar que participen. Esa cercanía hace que, a lo mejor pierdan el miedo a dar su opinión, ¿no? hoy, por ejemplo, estábamos valorando una ocupación, y uno de ellos ha considerado que no era adecuada por los kilómetros, por la distancia, yo no me puedo…pues, eso es un error, no simplemente… “¿estás seguro?”, intentar que ellos bueno, pues vean que es verdad que están equivocados. Que yo, cuando le corrija la práctica le diga que eso está mal y que, en vez de tener un tres en esa práctica, pues vas a tener un dos, pero bueno, eso ya es distinto, es simplemente, para intentar…

E: Darles confianza, ¿no?

P. 72: Darles confianza, porque, aunque soy seria, también participar, porque si no esto es una monotonía, yo explico, yo soluciono, yo… Es que si no ellos nunca pierden el miedo a dar su opinión. Además, como yo siempre les digo “tienen que estar fundamentados”, pues “mira Rosa”, “pues no, mira, te has confundido, ¿todo el mundo piensa igual?, ¿alguien opina lo contrario?”. Son formas para que…porque no es echarle una bronca porque se equivoque, es decir, tienes que darle una confianza y, además, que estamos aprendiendo y es normal. Entonces, esa forma, al menos de dar las clases a mí me viene bien para que ellos cuenten su experiencia si es que se trata de contar su experiencia o que ellos aporten la solución que han dado, y hacerles ver que no. Yo, cuando era alumna “ha cometido usted un error enorme”, era horrible, es que a la gente le daba miedo dar una solución porque te formaban allí… Es simplemente “no, te has confundido, esto no es así porque…mira, lee bien esto”, se lo intentas explicar, es que, si no, no participa nadie.

E: Y luego, me imagino que cuando estás con un grupo, las clases prácticas son diferentes, ¿no? Más pequeños grupos…

P. 72: Sí, sí, claro. Además, como yo he dado asignaturas en relaciones laborales, yo he tenido alumnos en segundo y ahora han pasado a tercero, entonces, de muchos de ellos me conozco el nombre y el apellido. Todos no suelo sabérmelos, pero, o bien el nombre o bien el apellido, digo esta o este es… Además, al hacer trabajos en grupo, cuando le voy corrigiendo ese trabajo en grupo, le voy diciendo “¿esta parte quién la ha hecho?, ¿tú?”, y se te va quedando el nombre, “pues mira, en esta parte te falta esto, esto o esto”, para que me lo entreguen en el trabajo final. Hacen entregas parciales, para que en el final tiene que estar incluido eso que yo le he dicho. Entonces, más o menos la relación es así. Aunque, ya te digo, soy seria, cumplo con mi horario, tengo que dar mis clases… Pero realmente es una posibilidad. Cuando yo estudié, esa cercanía no la había, eso yo lo puedo decir claramente, porque yo no abría mi boca ni levantaba la mano, era él el que “la que está situada en, que tiene tal y tal, que corrija…”. Así. Yo a veces no me sé el nombre, pero digo “dime tu nombre, no sé cuánto”, pero también incentivo primero, “venga, quién quiere ser voluntario, quién participa, quién opina lo contrario…”. Entonces, es totalmente distinto, antes te formaban… “cómo ha podido usted decir eso”, eso, yo, por lo menos, ahora, en mis clases no lo hago.

**Documento: 4. Ciencias Sociales y Jurídicas\P 73 CCSS Diseños**

**Peso: 0**

**Posición: 48 - 51**

**Código: 4. Acciones\Relación profesorado-alumnado\4.15. Importancia relación**

E; ¿Para ti es importante la relación que se establece entre alumnado y profesorado? ¿Por qué?

P. 73: Para mí es fundamental la relación profesor-alumno, porque si no mantienes un clima de confianza y una comunicación efectiva, difícilmente se podrán solucionar los problemas que tenga mi alumnado. Lo importante es poder crear un ambiente agradable en clase.

E: ¿Qué prácticas/ estrategias llevas a cabo para fomentar la relación con tu alumnado?

P. 73: Pues que sepan que pueden contar conmigo en cualquier momento. Siempre desde el respeto, por supuesto. Yo les doy hasta mi número personal de móvil, de Whassap, que muchos profesores lo critican, pero lo cierto es que nunca mis alumnos me han llamado o me han enviado mensajes a horas que no sean oportunas, que me los pueden mandar, porque yo por las noches pongo el móvil en silencio, pero nada, siempre que han tenido que consultarme algo han sabido que estoy a disposición de ellos.

**Documento: 4. Ciencias Sociales y Jurídicas\P 74 CCSS Diseños**

**Peso: 0**

**Posición: 54 - 59**

**Código: 4. Acciones\Relación profesorado-alumnado\4.15. Importancia relación**

E: Y en cuanto a la relación profesorado-alumnado, que ya me has comentado que para ti sí que es importante esta relación. ¿Qué haces para favorecer la relación con tu alumnado?

P. 74: Pues no sé, yo creo que no sigo ninguna estrategia, que es lo que me sale.

E: Sí, que eres más cercana y…

P. 74: Sí, por ejemplo, en la facultad de derecho de la universidad de Burgos, una cuestión que es que la mayoría del profesorado es joven, a ver, que son 45 años, pero yo me considero todavía joven, ¿sabes?

E: Claro.

P. 74: Entonces claro, eso también te facilita un poco el que no estés anclado en un viejo sistema, ¿no? Sino que te vayas un poco adaptando. Gente que tiene hijos jóvenes, hay hijos que están estudiando en la universidad… Entonces, tú ves la problemática que están teniendo y te vas adaptando… Yo creo que todo eso favorece, ¿eh? Somos muy cercanos la verdad, que parece feo decirlo, pero no soy yo, somos un grupo grande de personas que somos cercanas. O sea, estás tomando algo en la cafetería y te vienen y te preguntan algo y lo atiendes, o sea, no estás diciendo “no, mira, que estoy aquí tomando un café”, le atiendes, o sea que…

**Documento: 4. Ciencias Sociales y Jurídicas\P 75 CCSS Creencias**

**Peso: 0**

**Posición: 63 - 63**

**Código: 4. Acciones\Relación profesorado-alumnado\4.15. Importancia relación**

pero partiendo de la base de que la relación con este tipo de alumnos no deja de ser, entre comillas, muy superficial, porque es un alumno más, yo doy clase en un cuatrimestre a 285 alumnos.

**Documento: 4. Ciencias Sociales y Jurídicas\P 75 CCSS Diseños**

**Peso: 0**

**Posición: 108 - 117**

**Código: 4. Acciones\Relación profesorado-alumnado\4.15. Importancia relación**

E: Y, ¿para ti es importante la relación que se establece entre el alumnado y el profesorado?

P. 75: Importante no, es fundamental. Es que la enseñanza es recíproca.

E: Tú me das y yo te devuelvo.

P. 75: Claro, es que si no… Y, además, es lo que yo les digo, “no es que el profesor quiera enseñar, es que ustedes tienen que querer aprender”, y si el profesor le enseña, y de hecho, yo los años que más dificultad tengo de alcanzar, lo que estamos hablando, los objetivos y tal, es cuando, por las circunstancias que sea, se produce un mayor porcentaje de desconexión, o de no entendimiento con los alumnos, para mí es fundamental vamos.

E: Y, qué estrategias llevas a cabo para fomentar esa relación con el alumnado.

P. 75: Es que yo creo que ya es un poco…aunque solo sea de manera…por los treinta años de docencia, que sea…es una dinámica, que, en función de lo que estamos hablando, te vas adaptando y te vas…pero, por supuesto que para mí la estrategia es la de la incentivación a la participación, la de estar continuamente lanzando preguntas y haciendo toques de llamadas de atención, ir evaluando el interés… Yo hay veces que ha habido momentos que me he parado en seco y digo “¿no os interesa ya, nada de nada de lo que estamos hablando?” estoy en algo que estoy pensando qué es, pero les veo, y veo las caras, que ese día vienen de un examen, o por el motivo que sea…

E: Que están cansados.

P. 75: Y yo ese día digo “no tiene sentido seguir”, porque es lo que estábamos hablando, si tú estás en ese punto de desconexión, que, además, yo no me las quiero dar de experto en nada, pero yo me fijo mucho en la comunicación no verbal, muchísimo, yo analizo los gestos de los alumnos, y entonces, simplemente…

E: Te va dando información eso mismo.

P. 75: Y lo planteo. Entonces, llega un momento en que digo “¿no os interesa nada?, pues bueno, ya lo retomaremos o lo harán ustedes por su cuenta”, y corto, corto y paso a otra cosa, o intentamos ya terminar la clase, o darle la vuelta.

**Documento: 4. Ciencias Sociales y Jurídicas\P 76 CCSS Diseños**

**Peso: 0**

**Posición: 72 - 75**

**Código: 4. Acciones\Relación profesorado-alumnado\4.15. Importancia relación**

E: ¿Para ti es importante la relación que se establece entre alumnado y profesorado?

P. 76: Sí, sí, para mí es muy importante, porque el alumnado, hay alumnado que es tímido, hay alumnado con discapacidad, por ejemplo, precisamente, porque tiene esa discapacidad, muchos de ellos son tímidos, inseguros, y el hecho de que el profesor les conozca, que les dé su tiempo para hablar, le pregunte a él, ¿sabes? Eso les da confianza. Y a todos también, ¿eh? Yo soy de las que me acuerdo más de los nombres que de los apellidos, entonces, tuve un alumno, me acuerdo del nombre, hace dos años, y “Daniel, cómo estás”, me acuerdo de los nombres, me acuerdo más de los nombres que de los apellidos. Entonces, yo creo que eso les da a ellos esa seguridad que, en algunos casos es necesaria, a otros no, pero a algunos sí.

E: Y ¿qué prácticas o estrategias llevas a cabo para fomentar esa relación con el alumnado?

P. 76: Pues hablar con ellos, yo procuro hablar con ellos. Hago tutorías, por ejemplo, de los trabajos y esas cosas y ahí les conozco más y ellos también me conocen más ahí en esa relación porque soy más cercana. En la clase no siempre puedo hablar con todos, pero aquí, me permite conocerles más.

**Documento: 4. Ciencias Sociales y Jurídicas\P 77 CCSS Creencias**

**Peso: 0**

**Posición: 37 - 39**

**Código: 4. Acciones\Relación profesorado-alumnado\4.15. Importancia relación**

P. 77: Yo creo que soy cercana con los alumnos porque, pues igual que con un alumno con discapacidades, pues me fijo si uno se cortó el pelo, si uno se lo pintó, le pregunto, a alguno lo veo triste y le digo “¿qué te pasa? ¿tienes problemas en tu casa?” O sea, que tanto a los que tienen cierta discapacidad como a los que no tienen, pues soy muy cercana con ellos. Les pregunto muchas cosas, me intereso mucho por su vida personal porque me parece que está muy vinculada al desempeño en la asignatura.

E: Estupendo. Y, ¿cómo cree que la ven sus estudiantes?

P. 77: Pues yo creo que cercana también. Pero también creo que el problema, en cierto sentido, es que también soy un poco blandengue porque me mueven el corazoncito a la hora de evaluar, ¿no? Porque pienso que se sienten muy cercanos a mí, muy familiares, me cuentan los problemas que tienen y que no tienen nada que ver con la asignatura, vienen al despacho y me cuentan cosas...

**Documento: 4. Ciencias Sociales y Jurídicas\P 77 CCSS Diseños**

**Peso: 0**

**Posición: 58 - 61**

**Código: 4. Acciones\Relación profesorado-alumnado\4.15. Importancia relación**

E: Y, ¿para ti es importante la relación que se establece entre el alumnado y el profesorado?

P. 77: Sí, es importante, yo pienso que ellos deben de tener una relación de confianza para decirte hasta dónde y demás, y confiar en ti para poder amar la asignatura también. Sí, yo pienso que sí que es importante.

E: Y, ¿qué prácticas o estrategias llevas a cabo para fomentar la relación con tu alumnado?

P. 77: Pues mira, en principio intento preguntarles mucho y cuestionarles mucho. Por otro lado, intento aprenderme los nombres, que eso les sorprende un montón, y siendo un grupo tan grande, que de repente me sepa el nombre y apellido de uno, y se quedan un poco impresionados. Trabajo con la lista de clase en mano, entonces, leo Juan Pérez, y veo a Juan Pérez y me lo voy aprendiendo, y eso, pienso que ese trato de saberme nombres y eso les va a gustar mucho, al mismo tiempo que se van sintiendo comprometidos, porque cuando tú preguntas “¿un voluntario?”, siempre son los mismos tres, en cambio, cuando dices tú “Rafael Pérez”, y uno que está ahí intentando esconderse, pues ya lo incluyes sin darte cuenta, ¿no?

**Documento: 4. Ciencias Sociales y Jurídicas\P 78 CCSS Creencias**

**Peso: 0**

**Posición: 25 - 25**

**Código: 4. Acciones\Relación profesorado-alumnado\4.15. Importancia relación**

Yo cuando llego a clase lo primero que les digo es que cojan una cartulina y pongan el nombre en grande porque yo tengo miopía y no solamente por eso, sino porque normalmente tendemos al individualismo, a la frialdad, a no saber el nombre, como si fueran un número…

**Documento: 4. Ciencias Sociales y Jurídicas\P 78 CCSS Creencias**

**Peso: 0**

**Posición: 25 - 25**

**Código: 4. Acciones\Relación profesorado-alumnado\4.15. Importancia relación**

Cuando llego a clase les digo cómo me llamo y que me hablen de tú. Yo creo que todos y cada uno de nosotros sabemos dónde están nuestros límites y el tú o el usted no nos va a diferenciar de nada.

**Documento: 4. Ciencias Sociales y Jurídicas\P 78 CCSS Creencias**

**Peso: 0**

**Posición: 25 - 25**

**Código: 4. Acciones\Relación profesorado-alumnado\4.15. Importancia relación**

Creo que es la cercanía…también estoy muy criticada por los compañeros porque hay veces que dice que extralimito…yo creo que son miradas diferentes de la pedagogía, porque la distancia, el hacer esperar, el no contestar correos… No es mi modelo. Me siento muy identificada cuando encuentro profesores que son de la misma línea.

**Documento: 4. Ciencias Sociales y Jurídicas\P 79 CCSS Creencias**

**Peso: 0**

**Posición: 34 - 35**

**Código: 4. Acciones\Relación profesorado-alumnado\4.15. Importancia relación**

E: ¿Es muy importante eso para ti?

P. 79: Para mí, es importante que confíen en mí, que tengan una relación de confianza porque si no, no se puede trabajar bien con los alumnos y las alumnas.

**Documento: 4. Ciencias Sociales y Jurídicas\P 79 CCSS Creencias**

**Peso: 0**

**Posición: 59 - 61**

**Código: 4. Acciones\Relación profesorado-alumnado\4.15. Importancia relación**

Y a mí, me parece que es muy importante estar cerca de ellos, estar cerca y disponible para que ellos tengan la confianza de venir a tutorías y de plantearte dudas y qué tú puedas entrar en mayor profundidad con ellos.

E: Mostrarte accesible a ellos, ¿no?

P. 79: Sí, accesible.

**Documento: 4. Ciencias Sociales y Jurídicas\P 79 CCSS Diseños**

**Peso: 0**

**Posición: 97 - 102**

**Código: 4. Acciones\Relación profesorado-alumnado\4.15. Importancia relación**

E: Hablamos ahora de la relación profesor-alumno que también ha salido antes. Para ti, ¿es importante la relación que se establece entre el profesorado y el alumnado?

P. 79: Para mí, este es mi principal recurso.

E: ¿Por qué consideras que es importante?

P. 79: Porque estás trabajando con personas, entonces, tú tienes que tener una relación con ellos. Tú trabajas con ellos, y si trabajas con ellos, tú tienes que tener una relación con ellos, los tienes que conocer, tienes que estar cerca, tienes que ver de qué van, de qué no van, hasta dónde llegan, hasta dónde no llegan. Entonces, la relación es fundamental. Así, que creo que hay que intentar tener una buena relación, no ir de nada. Ni del profesor magnífico ni de cabrón.

E: Muy bien. ¿Tú qué estrategias pones en marcha para fomentar la relación con tu alumnado?

P. 79: Pues mira. Yo soy muy intuitiva. Yo no me planteo nada. A mí me sale, yo me dejo llevar.

**Documento: 5. Ciencias de la Educación\P80 EDU DISEÑOS**

**Peso: 0**

**Posición: 319 - 326**

**Código: 4. Acciones\Relación profesorado-alumnado\4.15. Importancia relación**

E: Ahora vamos a hablar un poquito de lo que es la relaciones entre el profesorado y el alumnado, que ya el otro día tú me contabas algo, en la otra entrevista ¿Para ti es importante la relación que se establece, que tú estableces con tu alumnado, entre el alumnado y el profesorado?

P80: A mí me parece muy importante.

E: Por qué.

P80: Hombre, porque a mí me gusta la cercanía, y yo creo que la cercanía ayuda mucho a motivar. Si tú te limitas a llegar, dar la clase, y no te preocupas de cómo se llaman, que yo intento aprenderme los nombres, aunque es muy complicado, pero me quedo con algunos, o les pregunto cómo están, o de vez en cuando les cuento algo gracioso, como este año lo de mi sordera, digo, “anda, ahora resulta que tengo más”.

E: Eso te iba a preguntar, que qué cosas haces.

P80: Y se parten de risa, “ah, nos vas a poner el oído en el examen”, digo, “pues no sé yo”, y les he puesto el ojo.

E: Que eso te iba a preguntar, que qué prácticas o qué cosas haces, qué estrategias llevas a cabo para fomentar esas relaciones, ¿no?, con tu alumnado.

P80: Bueno, hablar con ellos, preguntarles de vez en cuando cosas fuera de la asignatura, o decir, “venga, hoy ya hemos acabado, vamos a hacer otra cosa”, no sé.

**Documento: 5. Ciencias de la Educación\P81 EDU DISEÑOS**

**Peso: 0**

**Posición: 78 - 83**

**Código: 4. Acciones\Relación profesorado-alumnado\4.15. Importancia relación**

E: Preguntas ahora relacionadas con tu relación con el alumno. Para ti, ¿es importante la relación que se establece entre el alumnado y el profesorado?, ¿por qué?

P81: Yo creo que es muchísimo más importante que incluso lo que se puede aprender en la clase, desde mi punto de vista. En definitiva, nosotros podemos ser facilitadores, cualquier persona entra en una carrera para encontrar trabajo y nosotros, pues por nuestra situación, personal, profesional, o porque simplemente tenemos más años que ellos, tenemos más acceso a diferentes puestos laborales. Entonces, yo creo que lo principal es el acercamiento al alumnado.

E: ¿Qué medios utilizas tú para acercarte al alumnado?

P81: ¿Medios? Pues yo les doy a ellos mi teléfono, a mí no me importa que tengan mi teléfono, creo que puede tener sus connotaciones negativas por problemas que puedan ocurrir, pero bueno, de momento me ha ido bien. Yo les doy mi teléfono, tienen mi correo, por supuesto, el teléfono del despacho, yo siempre digo que estoy abierto a cualquier mensaje en cualquier momento del día o de la semana, y, por supuesto, yo, por ejemplo, una cosa que hago con las tutorías…sí es cierto que las tutorías me pongo un horario, pero siempre les digo a los alumnos que, si ellos van a venir a una tutoría que me avisen, y si quieren cualquier otro día que me avisen el día anterior y quedamos. O sea, que no me cierro solamente a las seis horas de tutoría porque tampoco tiene sentido.

E: ¿Cómo valoras tu relación con los estudiantes?

P81: Yo creo que es muy buena.

**Documento: 5. Ciencias de la Educación\P82 EDU CREENCIAS**

**Peso: 0**

**Posición: 17 - 17**

**Código: 4. Acciones\Relación profesorado-alumnado\4.15. Importancia relación**

Entonces, me cuesta mucho trabajo algunas veces, adaptar, no el contenido y tampoco las actividades, porque mis actividades están bastante adaptadas, pero sí la forma de relacionarme, entonces, intento ser muy agradable, muy amable, y al final ellos te lo agradecen un montón, y entonces, me da mucha alegría porque terminan viéndote, como que les ayudas, por poco que sea, porque no es que haga yo nada, pero por poco que sea, lo agradecen muchísimo. Entonces, te dan mucho cariño, a mí lo que me pasa es que me dan mucho cariño.

**Documento: 5. Ciencias de la Educación\P82 EDU DISEÑO**

**Peso: 0**

**Posición: 98 - 103**

**Código: 4. Acciones\Relación profesorado-alumnado\4.15. Importancia relación**

E: Vale. Me has hablado muchas veces de la relación que tienes con tus estudiantes, en la otra sesión me dijiste que eran muy cariñosos contigo y tal, ¿es importante para ti esa relación?

P82: Para mí sí. De hecho, yo siempre intento, el primer día siempre les cuento cosas de mi vida personal, dónde vivo, por qué vivo aquí, cómo me vine, si tengo o no novio, lo único que no les digo son los años que tengo porque eso, siempre da mucho juego durante mucho tiempo de la asignatura, saber…adivinar cuántos años tiene P82, y entonces, es muy gracioso. Pero siempre intento que sea, una…es cercano, pero sin pasarnos, quiero decir, que al final, tú eres mi alumno y yo soy tu profesora, y nos llevamos genial, y yo siempre al final de curso me voy con ellos a tomarme una cerveza…pero a mí me parece que una relación cercana favorece el aprendizaje de los alumnos.

E: Te iba a preguntar qué medios utilizas tú para comunicarte con ellos, aparte de cuando estás en clase con ellos…

P82: Por correo electrónico y la plataforma, sobre todo por correo electrónico de delegados, porque ellos siempre tienen un grupo de WhatsApp, con toda la gente de la clase, entonces, yo me comunico con los delegados y los delegados con los demás.

E: Cómo valoras tú tu relación con los estudiantes.

P82: Yo, muy buena. Vamos, yo me llevo muy…pero que ellos también conmigo, yo me llevo muy bien con ellos, y tengo…vamos, estoy muy contenta con la relación que tengo con ellos.

**Documento: 5. Ciencias de la Educación\P82 EDU DISEÑO**

**Peso: 0**

**Posición: 112 - 117**

**Código: 4. Acciones\Relación profesorado-alumnado\4.15. Importancia relación**

E: ¿Tú crees que todo el profesorado tiene la misma relación que tú con sus estudiantes?

P82: No.

E: Y qué te parecen esos tipos de relación.

P82: Bueno, cada uno, yo es que…

E: Cómo crees que debería de ser la comunicación para que fuera…

P82: Hombre, a mí me parece que la comunicación debería de ser como la tengo yo, pero tampoco sé si todo el mundo es capaz de hacerlo así. Quiero decir, es que yo, como la tengo tan abierta, tan normal y tan…me parece, yo estoy aquí muy…cuando tengo que poner un límite, lo pongo muy claro, pero que si no, vamos, yo soy muy flexible y a mí no me importa, pero que todo el mundo no es capaz de eso, también porque en la universidad, creo que lo dije el otro día, tenemos esa manía de controlar, o sea, de ser los poseedores del conocimiento y que los alumnos, están ahí, pero vamos, que yo me dedico, generalmente a la investigación y vengo a dar clase porque es obligatorio hacerlo, que en realidad no te gusta la docencia. Entonces, cuando no te gusta la docencia, el tipo de relación que vas a tener con tus alumnos es nefasto, porque no te gusta, entonces no quieres relacionarte con ellos. Tú te puedes encontrar dentro de clase, alumnos que te caen mejor, o que te caen peor, como pasa en la vida. Gente con la que tienes más feeling, gente con la que menos, pero, porque no te puedes llevar bien con todo el mundo, la cuestión es si tú de verdad crees que la docencia sirve, es que yo creo que hay compañeros que no se plantean el tipo de relación que tienen con los alumnos porque no creen en la docencia, entonces, es imposible cambiarlo.

**Documento: 5. Ciencias de la Educación\P83 EDU DISEÑOS**

**Peso: 0**

**Posición: 104 - 113**

**Código: 4. Acciones\Relación profesorado-alumnado\4.15. Importancia relación**

E: Qué tipo de relación tienes con tus alumnos, ¿es importante para ti esta relación?

P83: Sí.

E: Por qué.

P83: Yo no sé, lo considero un igual, no sé cómo decirte, me llevo muy bien con ellos. Si es verdad que a lo mejor mi forma de ser haya alguno que no le guste, pero en general, me suelo llevar muy bien con ellos. Creo que soy un buen apoyo para ellos y ellos para mí.

E: ¿Qué apoyos utilizas para comunicarte con ellos? Bueno, todos los que me has dicho antes, redes sociales…

P83: No, yo las redes sociales, las utilizamos, pero en ese aspecto, es una relación profesor-alumno, cuando ya dejan de ser mis alumnos, no tengo problema, pero no va más allá de eso.

E: Sí bueno, pero me refiero, que te relacionas con ellos a través de la plataforma, las redes… Aunque todo sea en relación a la asignatura… ¿pero te relacionas con ellos por esos medios o más en clases y tutorías?

P83: Sobre todo en clase y tutorías. Yo, clases, tutorías, correo electrónico, por teléfono… Pero yo, en los medios del aula virtual, a no ser que sea por un correo y tal, no la uso mucho la verdad. Y las redes sociales, cuando dejan de ser alumnos sí, pero mientras no, porque pueden generar poca objetividad y le cojas cariño a alguien y tú tienes que evaluarlo.

E: Cómo valoras tu relación con los estudiantes.

P83: Para mí es un 10 con ellos. Ellos para conmigo no lo sé.

**Documento: 5. Ciencias de la Educación\P83 EDU DISEÑOS**

**Peso: 0**

**Posición: 118 - 119**

**Código: 4. Acciones\Relación profesorado-alumnado\4.15. Importancia relación**

E: Pero cuál crees tú que es la mejor manera.

P83: La mejor manera es la que motive al alumno, ¿no? Conozco profesores que son los más siesos del mundo y motivan al alumno. A mí no me gusta darle un carácter más cordial, más de humor, aunque la primera semana no sea así, o sea, no sé. Yo digo lo que a mí me puede funcionar, pero tampoco tiene que ser eso ahí mundial, de hecho, podría ser un error. Estoy imaginándome algún profesor que haya tenido yo, interactuando como yo interactúo con los alumnos, y no le pegaría, sería super forzado. Me acuerdo de uno de derecho de Granada, que sería mortal, vamos.

**Documento: 5. Ciencias de la Educación\P84 EDU DISEÑOS**

**Peso: 0**

**Posición: 72 - 77**

**Código: 4. Acciones\Relación profesorado-alumnado\4.15. Importancia relación**

E: ¿Para ti es importante la relación que tienes con el alumnado?

P84: Muy importante. Yo he tenido la suerte que los alumnos que he tenido con discapacidad, han sido chavales muy risueños y muy alegres.

E: Me refiero en general.

P84: Sí, es muy importante. Quizás, con las personas tímidas que te comenté antes, pues me costaría un poco.

E: ¿Qué medios utilizas para comunicarte con ellos?

P84: Una aplicación móvil que se llama “Remind”, ahí pongo cualquier recurso, vídeo que yo vea interesante y eso le llega de inmediato al móvil. Lo utilizaba yo en el colegio para todas las circulares y nos ahorrábamos el papel.

**Documento: 5. Ciencias de la Educación\P85 EDU DISEÑOS**

**Peso: 0**

**Posición: 55 - 58**

**Código: 4. Acciones\Relación profesorado-alumnado\4.15. Importancia relación**

E: Vale. En cuanto a lo que sería la relación del profesorado y el alumnado, ¿para ti es importante la relación que se establece entre alumnado y profesorado?, ¿por qué?

P85: Claro, sí. Para mí es muy importante porque si yo estoy un poco como…si yo soy la guía para él en esa asignatura, pues yo quiero que el alumno se sienta con confianza pues, para comentarme sus dudas o sus miedos, sus intereses o sus motivaciones. Entonces, es importante porque si no creas un vínculo de confianza te pierdes mucha información que te llegue del alumno. Entonces, claro, eso lo hace el trato que vayas consiguiendo con ellos día a día, las metodologías que hagas, en las que el alumno participe más, como te encuentras de todo, alumnos más tímidos y alumnos menos, pero yo creo que es importante pues, para que por lo menos, que el proceso de enseñanza-aprendizaje sea un poco mayor, porque si no, te crees a lo mejor que están aprendiendo, y no, a lo mejor están pensando en otras cosas, entonces claro, es importante esa relación.

E: ¿Qué prácticas o estrategias llevas a cabo para fomentar la relación con tu alumnado?

P85: La comunicación, sobre todo, la escucha, el escuchar, el preguntarles. Como yo les digo, el hacer muchas preguntas, yo muchas veces soy muy pesada y les hago muchas preguntas, quiero indagar y quiero que ellos reflexionen y no, como yo les digo, no os creáis lo que yo os cuento ni lo que leéis, tenéis que…fomentar su curiosidad, su interés, ¿no?, el “¿esto es verdad?, ¿esto no es verdad?”, porque si no…Lo que sí percibo si no, es que se acostumbran a eso, a escuchar y escuchar y me quedo todo, y como yo les digo, “no, no, yo os cuento esto, pero no os lo creáis”. Entonces, muchas veces, se quedan descolocados, pero las estrategias que utilizo son esas, el preguntarles y que ellos se estrujen el cerebro por así decirlo.

**Documento: 5. Ciencias de la Educación\P86 EDU DISEÑOS**

**Peso: 0**

**Posición: 74 - 79**

**Código: 4. Acciones\Relación profesorado-alumnado\4.15. Importancia relación**

E: Bien, también cambiamos de preguntas, preguntas en relación a lo que es la comunicación, o lo que es la relación profesor-alumno. ¿Para ti es importante la relación que se establece entre el alumno y el profesorado, y por qué?

P86: Sí claro, es evidente, porque influye en el proceso de enseñanza-aprendizaje, en el caso de la música es evidente. La música se transmite del, lo que llaman los músicos, maestro, al músico, o sea, todos los músicos, cualquiera que sea, tiene su maestro y es de ese maestro del que han aprendido, o sea, la razón es obvia, y también en la didáctica, siempre hay alguien que te enseñó cómo hacer esto, cómo hacer aquello o cómo hacer lo otro.

E: ¿Qué medios utilizas para comunicarte con tus alumnos?

P86: En primer lugar, la voz, la comunicación presencial, digamos, y me es muy útil el correo electrónico, cada vez hago más tutorías electrónicas. Me gusta, también, queda escrito, no solamente para personas que lo necesiten, si no para otros estudiantes.

E: ¿Cómo valoras tu relación con tus alumnos, así en general?

P86: Yo creo que es buena.

**Documento: 5. Ciencias de la Educación\P87 EDU DISEÑOS**

**Peso: 0**

**Posición: 82 - 85**

**Código: 4. Acciones\Relación profesorado-alumnado\4.15. Importancia relación**

E: Bien. Voy a preguntarte ahora sobre la relación profesor-alumnado, en este caso, profesora-alumnado, ¿vale? También estuvimos hablando de esto en la entrevista anterior, pero, ¿es importante para ti la relación que tienes con tu alumnado?

P87: Súper. Muy importante. Esencial. Me parece que, si no están a gusto con la clase, con los compañeros o conmigo, no funciona todo lo que tiene que funcionar.

E: Bien. Cómo te comunicas tú con tus estudiantes, por qué medios.

P87: Bueno, por email, por supuesto, por la plataforma y, sobre todo, personalmente.

**Documento: 5. Ciencias de la Educación\P88 EDU DISEÑOS**

**Peso: 0**

**Posición: 68 - 75**

**Código: 4. Acciones\Relación profesorado-alumnado\4.15. Importancia relación**

E: Preguntas sobre la relación profesor-alumno, ¿para ti es importante la relación que se establece entre el profesorado y el alumnado?

P88: Importantísima, claro, la relación es fundamental. Aquí destaco dos vías, la vía técnica y la vía relacional. La vía técnica son todos tus conocimientos, cómo tú te preparas, cómo tú aprendes, tus creencias… Pero luego está la vía relacional, si eres mala persona y así es cómo te relacionas con ellos, poco vas a calar en ellos.

E: ¿Qué medios utilizas para comunicarte con tu alumnado?

P88: Correo electrónico, presencial, a través del delegado o delegada de grupo…

E: Muy accesible a ellos.

P88: Sí, sí. Además, todos los días miro el correo y contesto, que, a veces me dicen “uy, ¡qué rapidez!”.

E: ¿Cómo valoras tu relación con los estudiantes? Bueno, ahora estamos empezando el curso, pero en general.

P88: Yo creo que es buena, para mí es buena.

**Documento: 5. Ciencias de la Educación\P88 EDU DISEÑOS**

**Peso: 0**

**Posición: 78 - 81**

**Código: 4. Acciones\Relación profesorado-alumnado\4.15. Importancia relación**

E: ¿Consideras que se debe cambiar el modo en el que el profesor se relaciona con el alumnado?

P88: En general yo creo que ahora, en el siglo XXI no creo que las relaciones sean malas. Por los comentarios de los compañeros, las relaciones son positivas. Algún profesor que pueda tener un mal momento o un mal día, pero es una cosa puntual.

E: Creo que me has respondido, pero bueno, ¿de qué manera crees que debe ser esa relación alumno-profesor para que influya positivamente en el alumnado?

P88: Yo creo que debe ser lo más positiva posible y escuchando y poniéndose en el lugar de la otra persona.

**Documento: 5. Ciencias de la Educación\P89 EDU DISEÑOS**

**Peso: 0**

**Posición: 70 - 73**

**Código: 4. Acciones\Relación profesorado-alumnado\4.15. Importancia relación**

E: Y vamos a pasar ahora… Bueno, podríamos habernos extendido mucho en las metodologías, pero lo hemos visto de forma general, vamos a ver, porque también has comentado en algunos momentos la importancia de la relación que estableces con el alumnado, ¿no?, yo entiendo que para ti es importante y me gustaría saber por qué.

P89: Es fundamental. Todo lo que nos pasa en la vida ocurre en un momento emocional concreto, si tú estás tensa en una clase en la que consideras, que puedes quedar en evidencia por algo, hay mucha distancia entre el profesor y el alumnado. Si tú no estás cómodo, si no te sientes libre de opinar, si no estás en un contexto en el que te sientes respetado e incluso apreciado por tus compañeros, y también por el docente o la profesora, yo creo que los aprendizajes no son tan fluidos ni tan significativos. Además, que yo creo que, para dar ciertos contenidos y metodologías es necesaria una complicidad con el grupo. Y entonces, pues para mí, es muy importante, yo creo que los contextos emocionales positivos favorecen mucho más el aprendizaje…

E: ¿Y utilizas alguna estrategia práctica para fomentar esta…ya has dicho algunas, así como la bienvenida y tal… ¿Qué truquillos tienes así para favorecer estas relaciones?

P89: Pues, yo intento como ponerme un poco a su nivel. Hacerle bromas de las de su edad, hablar con algunas palabras que ellos utilizan, les hago bromas, intento que me vean cercana, que haya una relación simétrica entre ellos y yo… Pues no lo sé, a veces incluso nos vamos a tomarnos algo después de clase… No lo sé, yo creo que me siento como una más en el grupo, yo diría que, al final me encuentro como una más.

**Documento: 5. Ciencias de la Educación\P90 EDU DISEÑOS**

**Peso: 0**

**Posición: 90 - 95**

**Código: 4. Acciones\Relación profesorado-alumnado\4.15. Importancia relación**

E: Vale, en cuanto a la relación del profesorado con el alumnado, ¿para ti es importante la relación que se establece entre tu alumnado y tú?

P90: Para mí mucho, de hecho, yo trabajo con la pedagogía de la sonrisa, a veces no me apetece sonreír, pero, hago un esfuerzo por llegar a clase con una sonrisa y a veces los alumnos me dicen “P90, ¿siempre tienes ganas de reírte?” y digo, “pues no, a veces no tengo, pero cuando vengo aquí, hago el esfuerzo para que eso ocurra” y ellos lo agradecen, ¿no? Entonces, la pedagogía de la sonrisa te ayuda a, realmente, acercarte al alumnado, y transmitir el conocimiento con ilusión y motivación, y eso, me parece fundamental.

E: Y por qué crees que es tan importante esa relación.

P90: Hombre, pues porque, yo creo que la relación bidireccional entre un docente y un discente pues, garantiza de una manera muy significativa el desarrollo de todo proceso didáctico.

E: Vale, bien. Y qué prácticas o estrategias, además de la pedagogía de la sonrisa, llevas a cabo para fomentar esa relación con el alumnado.

P90: Bueno, pues propicio mucho el poner ejemplos de mi vida cotidiana, que piensen que soy una persona más, que soy uno más en medio de este proceso. O sea, mostrarme cercano, una persona en la que se puede, de alguna forma, no sé, confiar en ella, hablar con ella…

**Documento: 5. Ciencias de la Educación\P91 EDU DISEÑOS**

**Peso: 0**

**Posición: 52 - 55**

**Código: 4. Acciones\Relación profesorado-alumnado\4.15. Importancia relación**

E: ¿Para ti es importante la relación que se establece entre alumnado y profesorado? ¿Por qué?

P91: Sí, muy importante. Porque el profesorado, en definitiva, pese a que yo quiera hacer un conocimiento horizontal, es el “dinamizador”, es el guía, es el impulsor del conocimiento del alumnado.

E: ¿Qué prácticas o estrategias llevas a cabo para fomentar la relación con tu alumnado?

P91: Bueno pues, las múltiples tipos de metodologías docentes que doy, los múltiples tipos de materiales o de soportes en los que enseño el contenido que doy, las evaluaciones, por ejemplo, que las dialogo y son ellos los que deciden qué tipo de evaluación van a tener, la capacidad de decisión que les doy dentro de mis clases de una manera muy autónoma con los debates y creo que el tratarlos de una manera más adulta, más autónoma y más responsable genera una relación, también, de empatía con el profesor muy grande.

**Documento: 5. Ciencias de la Educación\P92 EDU DISEÑOS**

**Peso: 0**

**Posición: 58 - 61**

**Código: 4. Acciones\Relación profesorado-alumnado\4.15. Importancia relación**

E: Vale. ¿Qué recursos utilizas para comunicarte con tus alumnos?

P92: Moodle siempre que a través del correo electrónico. Solo admito este correo para que todo quede registrado, no admito ni Gmail, ni Hotmail. Además, están expuestos mis horarios de tutorías presenciales.

E: ¿Cómo valoras esta relación con tus estudiantes?

P92: Muy bien, siempre me ha gustado mucho. Yo me dedico a esto porque me gusta trabajar con las personas.

**Documento: 5. Ciencias de la Educación\P93 EDU CREENCIAS**

**Peso: 0**

**Posición: 17 - 17**

**Código: 4. Acciones\Relación profesorado-alumnado\4.15. Importancia relación**

El primer día, en plan en privado, entras, ves un poco, ¿no? Yo creo que lo hacemos todos. Intentar reconocer nombre-foto, “no voy a pasar lista todos los días, tranquilidad”, pero sí que ahora un primer contacto, que yo, por lo menos, soy un desastre con los nombres, pero con las fotos sí que reconozco.

**Documento: 5. Ciencias de la Educación\P93 EDU CREENCIAS**

**Peso: 0**

**Posición: 19 - 19**

**Código: 4. Acciones\Relación profesorado-alumnado\4.15. Importancia relación**

Yo le digo a todo el mundo que “por supuesto, cuando queráis me lo pedís presencialmente, me lo pedís por correo, quedamos un momento determinado y nos vemos”. Yo se lo digo así, no establezco calendario salvo con trabajos en grupos en que se lo pida, pero entonces es a todo el mundo por igual.

**Documento: 5. Ciencias de la Educación\P93 EDU CREENCIAS**

**Peso: 0**

**Posición: 32 - 39**

**Código: 4. Acciones\Relación profesorado-alumnado\4.15. Importancia relación**

E: ¿La alumna en silla?

P93: No, la alumna.

E: La alumna con discapacidad visual.

P93: Sí, entonces yo creo que en ese sentido sí que lo hemos trabajado y ya te digo, que me consta eh, me consta.

E: Porque se sentía más segura, ¿tú crees?

P93: Posiblemente, sí. Y a mí me ha costado, a base de ir hablando de, cómo te digo, yo no quiero que lo que en principio sea para ayudar termine hiriendo, o sea, para nada, ¿no? Y bueno, “que no es que sea nada de lo que tienes que avergonzarte, en absoluto, todo lo contrario, siéntete orgullosa”. Yo creo que todos estos años, sí que le han servido a ella para ir mejorando en cuanto a exteriorizar pues, “bueno, que tienes esta situación y no pasa nada”.

E: A ganar confianza también.

P93: A ganar confianza, claro. Pero ya te digo, o sea, lo que, si es beneficioso o no, perjudicial creo que no lo es para nada, me parece poca inclusión es si no está trabajando en grupo y lo que te digo, o sea, mi experiencia en estos dos casos varía mucho de cómo es la persona, si ser extrovertida completamente a una persona mucho más tímida. No sé ahí hasta qué punto estamos hablando de variable discapacidad o de variable…

**Documento: 5. Ciencias de la Educación\P93 EDU CREENCIAS**

**Peso: 0**

**Posición: 65 - 65**

**Código: 4. Acciones\Relación profesorado-alumnado\4.15. Importancia relación**

somos profes, pero la cercanía sí que creo que es importante. O hay un clima de cercanía con normas y con límites o no conseguimos lo que queremos.

**Documento: 5. Ciencias de la Educación\P93 EDU DISEÑOS**

**Peso: 0**

**Posición: 15 - 16**

**Código: 4. Acciones\Relación profesorado-alumnado\4.15. Importancia relación**

P93: Atención. O sea, acercarte a estas personas y hablarles, decirles “pues mira, he recibido esto, estas indicaciones. Tú en concreto, pues qué crees que es lo que tengo que hacer para que puedas seguir bien la clase…”. O sea, lo primero desde luego es hablar con las personas.

E: La comunicación profesorado-alumnado.

**Documento: 5. Ciencias de la Educación\P94 EDU DISEÑOS**

**Peso: 0**

**Posición: 32 - 35**

**Código: 4. Acciones\Relación profesorado-alumnado\4.15. Importancia relación**

E: En relación a tu relación con el alumnado que para ti es muy importante. Nos puedes decir qué prácticas o estrategias llevas a cabo para fomentar la relación con tu alumnado.

P94: A ver estrategias creo que ninguna. Una de las cosas que yo si trabajo para intentar no dejarme llevar por la vanidad, ya que los profesores nos sentimos muy bien al saber que sabemos muchos, pues yo hago un ejercicio de humildad cuando yo entro en las clases, ya que pienso que los estudiantes que tengo delante, pues dentro de cuatro años serán mis colegas de profesión. Esto para mí fue un autentico descubrimiento. Es que cuando yo descubrí que dentro de cinco años o de cuatro estos estudiantes se iban a convertir en colegas míos, pues me hizo desarrollar una serie de estrategias de conexión con el alumnado que me hace ser tremendamente cercana y valorada por el estudiantado como ser humano y no como algo divino y todo esto me permite ofrecerles lo que sé y lo que no sé. Entonces, ellos perciben que soy humilde y honesta con lo que les ofrezco. Entonces, yo creo que son competencias y habilidades afectivos-emocionales.

E: ¿Utilizas, por ejemplo estrategias como llamarles a cada uno por su nombre o tienes algún tipo de relación…?

P94: Los primeros días de clase yo me comprometo a saberme los nombres de los alumnos y les explico el por qué. Es que el mero hecho de saberte los nombres demuestra respeto, cercanía y nivelación, es decir, para que ellos sientan que yo les tengo en cuenta.

**Documento: 5. Ciencias de la Educación\P95 EDU DISEÑOS**

**Peso: 0**

**Posición: 64 - 67**

**Código: 4. Acciones\Relación profesorado-alumnado\4.15. Importancia relación**

E: Vale. En cuanto a la relación del profesorado con el alumnado, ¿para ti es importante esa relación que se establece entre el profesorado y el alumnado?

P95: Hombre, sí. En una asignatura como es la de matemáticas, me parece muy obvio que un profesor que te guste, hace que a lo mejor una materia que al principio te resulta muy difícil o que tú te encuentras muy limitado en ella, que le pasa a muchos estudiantes, tener un profesorado accesible, que te guste como explique, que te entienda, les influye muy positivamente a perder el miedo, a desbloquearse y a intentar superar esas limitaciones. Con lo cual, yo eso lo tengo clarísimo, porque cuando les pregunto me dicen, “a mí se me daba fatal las matemáticas, hasta que me encontré con un profesor en secundaria que hizo que me encantara y a partir de entonces, tal”. Entonces, para mí en matemáticas es muy fácil que un profesor pueda hacer que las aborrezcas o las ames. Por lo cual, intento que la relación sea cercana y que por lo menos, no infundirles miedo ni rechazo por la asignatura, sino intentar ilusionarles, aunque sé que eso es muy difícil.

E: ¿Qué prácticas o qué estrategias llevas a cabo para fomentar esa relación?

P95: Pues, ya te digo, intentar darles la voz, que sean ellos los protagonistas desde el primer momento, y luego, pues en los grupos grandes, intento ser muy cuidadosa en la forma de contestar cuando algunos hacen preguntas. En matemáticas, a los estudiantes les cuesta mucho preguntar y si preguntan a lo mejor creen que el profesor les puede decir que cómo se atreve a hacer esa pregunta que eso ya lo debería saber. Este tipo de comentario, seguramente que han recibido mucho a lo largo de sus vida, y yo pues intento tener mucho cuidado con eso. Y luego, utilizo el sentido del humor muchas veces porque creo que eso hace que te vean más cercano. Y también, intento transmitirles la utilidad de las cosas que estamos aprendiendo, la aplicación práctica en el aula, y además, les intento comunicar que las matemáticas pueden ser creativas, entretenidas, divertidas y útiles.

**Documento: 5. Ciencias de la Educación\P96 EDU CREENCIAS**

**Peso: 0**

**Posición: 28 - 29**

**Código: 4. Acciones\Relación profesorado-alumnado\4.15. Importancia relación**

E: Con respecto al rol docente y a las actitudes de los docentes, ¿podrías decirme algunas características que te definan a ti como docente?

P96: Yo creo que soy muy humilde y muy cercana al alumnado. Entonces, al ser muy cercana al alumnado, el alumnado te habla con sinceridad. Y eso hace que te enteres de todo, de todos sus miedos, y si tienen, verás, si tú conoces sus miedos puedes actuar sobre ellos, si no los conoces no. Entonces, sí es cierto que, el poder dialogar con el alumnado es lo importante, y, además, es realmente, para el profesorado que piensa que es mejor que no hablen, digamos, es totalmente al contrario porque si sabes lo que piensan, puedes defenderte. Que incluso hasta en esa opción es más favorable que puedas hablar con ellos y con ellas porque te van a decir hasta dónde, qué es lo que les gusta o lo que no les gusta, qué es lo que les está viniendo bien y lo que no, cuáles son las propuestas de mejora, que siempre las hay en una asignatura, además, cuando se repite a lo largo del tiempo. Y, yo creo que eso es, al menos lo que me dice el alumnado.

**Documento: 5. Ciencias de la Educación\P96 EDU DISEÑOS**

**Peso: 0**

**Posición: 56 - 61**

**Código: 4. Acciones\Relación profesorado-alumnado\4.15. Importancia relación**

E: En relación a lo que sería la relación profesorado-alumnado, ¿para ti es importante la relación que se establece entre alumno y profesorado? Y, ¿por qué?

P96: Totalmente. Yo creo que es muy importante. Es muy importante porque, en realidad, tú tienes una responsabilidad como docente, no solo académica, sino también con un alumnado que además son chavales a punto de...en mi caso de cuarto, están a punto de salir como docentes, con muchos miedos, con muchas responsabilidades también para ellos y yo creo que es necesario un buen clima. Además, si no hay un buen clima, después no hay inquietud por aprender. Y entonces, esto luego se extiende a cafetería, se extiende a los pasillos y se extiende a todos lados. Y es importante porque, aunque sí es cierto que el respeto...yo siempre hablo del respeto, que el respeto no va en el usted, el respeto va en que tú no quieras hacerme daño a mí y me tengas en cuenta antes de hacer daño de la misma forma que yo lo voy a hacer contigo. Y eso se nota. Y cuando tienes ese clima no hay...además la confianza en ti hace que puedas trabajar cosas que un principio hubiesen dicho que no.

E: Claro.

P96: Y se esfuerzan más y trabajan muchísimo. Trabajan mucho, trabajan un montón.

E: ¿Qué prácticas o estrategias llevas a cabo para fomentar la relación con tu alumnado?

P96: Nosotros hacemos muchas dinámicas. Muchas dinámicas en relación a conocernos, a hablar de nosotras mismas. De hecho, yo me presento yo con mis historias. Es decir, yo me presento como profesora de universidad, si soy especialista en tal, pero, además, tengo tres hijos, soy...familia, mis amigos son estos, yo...lo que espero de esta asignatura es esto, yo espero de vosotros esto y “tengo mi Facebook, si queréis contactar conmigo. Además, por si queréis verme, queréis conocerme pues podéis tal en el Facebook”. Y entonces yo ya si es cierto que tienes ahí un...tú también sales de tu zona de confort porque te estás mostrando al gran público, que tiene sus pros y sus contras. Entonces, ellos ven en eso la confianza necesaria para poder también abrirse y poder contar y explicar sus circunstancias distintas.

**Documento: 5. Ciencias de la Educación\P97 EDU DISEÑOS**

**Peso: 0**

**Posición: 62 - 65**

**Código: 4. Acciones\Relación profesorado-alumnado\4.15. Importancia relación**

E: ¿Para ti es importante la relación que se establece entre el profesorado y el alumnado?

P97: Sí, para mí es muy importante, y sin embargo, sé que tengo problemas sobre cuáles son los límites de las relaciones. Esto es también, una crítica muy gorda que nos hacen muchas veces, y nos dicen “que no podemos ser tan mamaítas de nuestros estudiantes”. Y yo, pues me lo tomo como un alago, cuando me dicen lo de mamaíta. Pero sí que es cierto, que establecer un límite, en plan, hasta dónde es un alumno y no es tu amigo, etc., para mí eso es muy difícil, porque al final, muchos de ellos, terminan siendo amigos nuestros prácticamente y terminan pues, trabajando con nosotros en los proyectos, terminamos dirigiéndoles las tesis y esas cosas.

E: Y ¿qué estrategias utilizas para fomentar esa relación con el alumnado?

P97: Creo que sobre todo la cercanía, el humor, ya que muchas veces, a pesar de ser una persona muy tímida, creo que al entrar en el aula me transformo, intento llamar la atención para que vean que me importa. El otro día, me di cuenta que había dos alumnos que no habían estado en la clase pero que estaban en pequeños subgrupos. Pues les escribí un correo y le dije “oye me he dado cuenta que no habéis venido a la clase de gran grupo que era después, ¿os ha pasado algo?”, así, ellos se dan cuenta que tú estás interesada, o lo ves por los pasillos y les dices “oye me debes una clase porque el otro día no viniste”, o también, muchas veces, les cuento experiencias personales, como por ejemplo, las experiencias que vamos viviendo con los coles. También, les gusta que muchas veces, me ponga en plan muy especialista y les cuente pues, cosas que a lo mejor les interesa, como cosas de Psicología, de cómo son los niños y niñas cuando nacen, es que los aspectos psicológicos les interesan mucho. Y, además, como lo ven de una manera muy técnica y muy teórica, pues algo que no le interesa, pues desmotivan, pero cuando tú le cuentas como es el crecimiento de un niño o una niña, el primer tipo de encuentro, etc., todo este tipo de experiencia relacionado con la Psicología, pero mucho más vivencial, les interesa bastante.

**Documento: 5. Ciencias de la Educación\P98 EDU CREENCIAS**

**Peso: 0**

**Posición: 33 - 33**

**Código: 4. Acciones\Relación profesorado-alumnado\4.15. Importancia relación**

A ver, los alumnos antes de venir a clase a conocerme, yo le envío una carta donde más o menos les explico quién soy y qué vamos hacer en las clases. Después, cuando ya nos conocemos, dedico pues un par de semanas a…no doy ningún tema, pero creo que es muy buena inversión. Verás, no les hablo de la asignatura, pero sí que les explico la estructura, el temario, sobre lo que vamos hacer, ellos me dicen por qué han elegido la asignatura, etc., es decir, todo el tema de crear lazos de comunicación, ya que esto es muy importante porque después plantas algo y es más fácil que crezca.

**Documento: 5. Ciencias de la Educación\P98 EDU DISEÑOS**

**Peso: 0**

**Posición: 42 - 43**

**Código: 4. Acciones\Relación profesorado-alumnado\4.15. Importancia relación**

E: Hemos hablado ya en entre la relación que existe entre el profesorado y el alumnado y la importancia que tú le das a esa relación y a cualquier relación humana. Entonces, en clase qué estrategias llevas a cabo para fomentar esa relación. Hemos dicho sentarnos en círculo y así nos vemos y hablamos…entonces, qué más.

P98: Pues, la relación es mínima porque vamos todos muy deprisa y los alumnos tienen que atender mucho fuego y los profesores también. A veces, puede ser también por tema de carácter. Yo es que soy una persona muy tímida. Yo cuando entro en un sitio no empiezo a saludar a todos ni nada, sino que yo entro en silencio y procuro sentarme sin molestar. Conclusión, que a los alumnos los trato como mucho respeto y demás, pero que no me voy a cenar con ellos ni mucho menos. A mi cada año me cuesta mucho trabajo el poner una nota exacta, porque yo no sé cómo lo pueden hacer mis compañeros cuando ponen 3,568 y yo me veo incapaz de hacer esto y mira que lo he intentado. Además, en las clases siempre hay un espacio libre para que ellos me pregunten o para que yo pregunte. Además, me muestro accesible por la tutoría virtual, pero siempre digo que los fines de semana no voy a contestar, pero siempre acabo contestando aunque me enfado conmigo mismo. Ahora no siempre consigo comunicarme con mis alumnos, este año, por ejemplo, he tenido un alumno que se ha ido y no he sido capaz de comunicarme con él, ni por teléfono ni por correo electrónico. Pues no sé uso cosas sencillas, el tema de los cuentos, por ejemplo, que son cosas cotidianas o de actualidad, o que venga alguien de fuera a dar la clase, pues son detallitos que parece que no tienen importancia, pero sí que la tienen y mucho y ayudan a tener un buen clima. Pero tampoco es todo felicidad, pero sí que hay un buen ambiente y podemos hablarnos tranquilamente.

**Documento: 5. Ciencias de la Educación\P99 EDU DISEÑOS**

**Peso: 0**

**Posición: 52 - 55**

**Código: 4. Acciones\Relación profesorado-alumnado\4.15. Importancia relación**

E: Vale. En cuanto a la relación que existe entre el profesor y el alumno, ¿para ti es importante esta relación?

P99: Sí. Cuando no haya un poco de empatía mal vamos, porque he tenido la experiencia de que, si no me gusta este profesor, su asignatura ya se estudia menos. Entonces, yo pienso que la relación es fundamental.

E: Vale. Y, ¿qué prácticas o estrategias llevas a cabo para fomentar esa relación que existe entre el profesorado y el alumnado?

P99: Nada, la propia clase. Hay clases que son más cercanas y otras que se mantiene más distante. En principio, cuando damos la teoría, al yo estar preguntando y ellos respondiendo, ya es un momento más de cercanía. Cuando luego, están haciendo las prácticas y te acercas de uno en uno y tal, pues también es un momento más cercano porque es más individual, pero no hay ninguna estrategia pensada para intentar ser cercano. Es que yo soy así, no me lo propongo.

**Documento: 5. Ciencias de la Educación\P100 EDU DISEÑOS**

**Peso: 0**

**Posición: 70 - 77**

**Código: 4. Acciones\Relación profesorado-alumnado\4.15. Importancia relación**

E: Estupendo. En cuanto a la relación del profesorado y el alumnado, ¿para ti es importante esa relación?

P100: ¿La relación profesor-alumnado?

E: Sí.

P100: Hombre, es necesaria total y absolutamente, lo que pasa es que, a veces es difícil, es decir, en el caso mío concreto, me falta tiempo, es decir, no puedes crearte una relación muy estrecha con un alumnado que lo ves un día a la semana dos horas y que hay 90 alumnos. Como te comentaba antes, ha habido grupos en los cuales te has implicado más porque las características concretas de ese grupo, requerían de eso, acudían más a ti, han ido a tutorías y tal, y ha habido grupos pues, que no han requerido de tanta relación interpersonal. Realmente es muy difícil, en un grupo de 90 alumnos, un profesor que va dos horas a la semana, en mi caso, si me hablas del Centro de Adultos, te diría que total y absoluta, es decir, aquí nos vamos hasta de cena juntos, pero en la universidad con 90 alumnos y el tiempo que tienes, pues es muy difícil.

E: Sí, es difícil. Por qué le das importancia a esta relación entre el profesor y el alumno, a la comunicación, aunque sea escasa por las condiciones o por las circunstancias, por qué es importante.

P100: Hombre, porque si tú conoces toda la realidad que envuelve al alumno en un momento determinado, le puedes ayudar de mejor manera. A parte de las personas con algún tipo de problema que me pueda indicar la USE, que están claramente diagnosticados, hay personas que tienen otro tipo de problemas, problemas personales, familiares, etcétera, etcétera, que en un momento determinado, pues son tan importantes como lo otro a la hora del rendimiento académico, y, por tanto, si tuviésemos esa vinculación, sería conocedor de esos problemas, y muchas veces pues no puede ser porque no llegas, pero como todo, es importante conocer, como el médico conoce a sus pacientes, pues tú, a tu alumnado, es importantísimo conocerlo. Pero en la universidad, las relaciones son más impersonales, evidentemente, por este problema de tiempo y de número de alumnado.

E: Estoy totalmente de acuerdo. Qué prácticas o estrategias utilizas para fomentar esa relación.

P100: Bueno, pues yo, intentar ser cercano, es decir, yo, lo que te he comentado de dar mi número de teléfono del despacho a una hora por la tarde, pues, no es una cosa muy natural, muy normal, quiero decir, que yo creo que ahí demuestro que tengo, por lo menos, interés en que la persona, en un momento determinado, si me requiere y me necesita, pueda contar con ese recurso. Yo no tengo por qué hacer eso, yo, mi obligación es cumplir con mi horario de la universidad, irme a mi otro trabajo y olvidarme completamente de eso, pero yo creo que, también por mi forma de ser y conforme he trabajado toda la vida con personas adultas con este tipo de vinculación, pues, y más, yo trabajo en mi pueblo, es decir, yo voy por la calle y voy contestando las dudas o problemas que tengan…un pueblo de 100.000 habitantes, pues, yo voy matriculando a la gente en las fiestas del pueblo, es decir, vienen “mira, que quiero apuntarme al curso de no sé qué”, y mi mujer dice, “¿y tú no puedes desconectar?”, pues no, porque forma parte de mí. Entonces, yo creo que esto forma parte de mi carácter y se ha ido creando a través de eso. Evidentemente, de la universidad…bueno, me ocurren casos, ¿eh?, mis vecinos de mi casa son alumnos míos este año, bueno, y coincide que han estudiado magisterio, les ha tocado conmigo, y encima uno es amigo de un hijo mío, pues bueno, ahí hay una vinculación más cercana por circunstancias y por el azar, ¿no?, pero, en general, yo intento ser cercano porque es mi forma de ser.

**Documento: 5. Ciencias de la Educación\P101 EDU CREENCIAS**

**Peso: 0**

**Posición: 103 - 103**

**Código: 4. Acciones\Relación profesorado-alumnado\4.15. Importancia relación**

Y después, el primer día de clase, digo “venga, que voy a pasar lista”, y los pongo en fila, por orden alfabético, un juego, y se tienen que ordenar en función de la altura, del lugar de residencia… Y digo, “ea, y ahora os ordenáis alfabéticamente”, y pum, y voy con el móvil grabándoles mientras ellos van diciendo sus nombres, y yo después, eso, pum, pum, a memorizarlo. Si te sabes sus nombres, es increíble cómo cambia la cosa, incluso después, al cabo del tiempo, de lo que se acuerdan es de que tú te sabías sus nombres. Y, si, además, que eso viene en la ficha, te sabes sus cumpleaños y los llamas cuando es su cumpleaños, pues, es un detalle, ¿no? Un correíllo “mira, que felicidades, que tal”, pues ya te los tienes ganados, ¿no? Eso, que son trucos que son emocionales, que no eres un bulto, que no eres un número. Lo de los cumpleaños, como hay veces que lo consideran como algo invasivo, ¿no? Dices, “¿qué haces tú un domingo llamándome pare felicitarme?”, mi teléfono, mi tal… Y entonces, pues lo que haces es que el viernes en clase le dices “oye, felicidades adelantadas, ¿eh?”, entonces… Son truquillos de esos. Y después, en las actividades, por ejemplo, participar con ellos y jugar con ellos, que te vean que tú eres uno más. Después, cada vez que hay una clase en la que faltan, ¿no? que te dicen “pues oye, es que no puedo ir porque no sé qué, no sé cuánto”, ¿no? pues ellos te están contando parte de su vida y, normalmente, es por problemas que tienen de enfermedad o de familiares, o por temas de trabajo y, oye, yo con eso, les respondo les digo “gracias por avisar, espero que te recuperes, o que no sea grave…”, ¿no? Y después, para la siguiente clase, yo me lo apunto para preguntarle, porque oye, si alguien te dice que se le está muriendo la madre y a la siguiente clase pum, pum, pues oye, esto qué es. Es el tratarlos como tratarías a cualquier persona, ¿no? Oye, si te ha contado eso…si no te lo cuenta, pues no lo sabes, pero si te lo ha contado, pues oye, pregúntale en la siguiente clase, ¿no? Yo creo que es un poco eso, el ambiente emocional. Si consigues crear el ambiente ese, pues el aprendizaje es más probable que fluya. Después, también, dar feedback individuales. Si alguien participa en alguna tarea, pues “oye, muchas gracias por participar en el foro, muy interesante”. Reforzar, si lo ha hecho bien, pues oye, díselo, ¿no? Dile que lo ha hecho bien o refuérzale en ese aspecto, ¿no? También hay una encuesta inicial donde ellos me tienen que contar si han trabajado antes, la formación previa que tienen, qué es lo que esperan de la asignatura…

**Documento: 5. Ciencias de la Educación\P101 EDU CREENCIAS**

**Peso: 0**

**Posición: 103 - 103**

**Código: 4. Acciones\Relación profesorado-alumnado\4.15. Importancia relación**

El otro día, teníamos que hacer una relajación y tal y había una chica que tenía problemas de otro tipo y que le gusta Alejandro Sanz, pues pones la música y pum, de Alejandro Sanz para la relajación, pues ya te la tienes ganada, ¿entiendes? Se trata de que la planificación de la sesión, no hacerla solo con el contenido exclusivamente, sino saber que estás trabajando con personas y que las personas tienen emociones, ¿qué te gustaría que hicieran contigo? Pues hacerlo tú con ellos. Y, entonces, bueno, de eso se trata. Yo, hay veces que antes de la primera clase, mis compañeros se ríen porque te dicen “qué haces”, y dices “estoy preparándome la primera clase y memorizando los nombres”, ¿no? Con las fichas, tal, tal, tal, y ellos “pero bueno, esto qué es”, y sí, es que es más importante que les llame por su nombre a lo que les vaya a contar, y más en este tipo de trabajo donde ellos en el futuro van a trabajar con personas. Cómo vas a estar tú con alumnos de los que no te sepas ni el nombre, ¿no? ¿Qué currículum oculto les estamos transmitiendo?

**Documento: 5. Ciencias de la Educación\P101 EDU DISEÑOS**

**Peso: 0**

**Posición: 31 - 31**

**Código: 4. Acciones\Relación profesorado-alumnado\4.15. Importancia relación**

Entonces, bueno, tienes ahí eso, y se trata de eso, de tener tiempo para conocerlos, o sea, ya, esa alumna era antes una alumna que tenía un nombre árabe, ¿no? Pero, ya, a partir de ahora, esa alumna para mí es diferente, digamos, más persona, el compromiso con ella es todavía mayor, ¿no? Cada alumno que tienes es un proyecto de vida, que tiene una familia, tal, un esfuerzo… Joder, es una responsabilidad, ¿no? Entonces, si tú solo tienes tiempo para ir y soltar las diapositivas y los alumnos son bultos, pues…por eso digo yo lo del tiempo.

**Documento: 5. Ciencias de la Educación\P101 EDU DISEÑOS**

**Peso: 0**

**Posición: 52 - 55**

**Código: 4. Acciones\Relación profesorado-alumnado\4.15. Importancia relación**

E: Vale. Y pasamos ahora a la relación entre el profesorado y el alumnado, ¿crees que es importante esta relación? Ya me has hablado antes más o menos de esto…

P101: Sí, es fundamental, y eso es lo que te digo, los mejores profesores que he tenido eran encantadores y tuve una relación fantástica y no concibo lo contrario. Un profesor que nos tratara mal, nos faltara el respeto o nos tratara de manera despectiva y tal y que yo lo considere buen profesor y considere que haya aprendido con él, ¿no? quizá puede haber algunos que sean más o menos autoritarios, pero esa relación… Yo recuerdo a uno que era bastante autoritario y tal, pero que después nos invitaba a desayunar, y a su casa y tenía gallinas y tal, rompía esa barrera, y nosotros nos volcábamos con esa asignatura, aunque era muy exigente y tal, le echábamos una cantidad de horas que no le hubiésemos echado si él no hiciese eso, ¿no? entonces, la relación…para que exista un aprendizaje tiene que haber una atmósfera y un ambiente de aprendizaje. Lo contrario puede ser memorización o puede ser algún tipo de aprender…pero es muy complicado si no hay…y hay quién dice, “es que entonces te van a perder el respeto” ¿no? a mí no me ha pasado nunca eso. Sí que he tenido a lo mejor alumnos más avispadillos que me la han intentado colar, ¿no? pero de esos que me han dicho “no, yo he ido a todas tus clases”, y a lo mejor no han venido, ¿no? Y claro, tú dices “tú por qué me quieres engañar”, y dicen “a ver, dónde están las listas de firmas”, digo “ah, que es eso, ¿no?”, entonces le dices “bueno, mira, tenemos quinientas fotografías que hemos hecho a lo largo del curso, búscate en alguna y si te encuentras”, y entonces claro, se desmontaban. Entonces, son situaciones tensas con algunos alumnos, que a lo mejor han aprovechado el ambiente relajado de clase para aprovecharse de la situación, ¿no? pero incluso yo creo que merece la pena correr ese riesgo. Kiko, en el curso de profesores nóveles nos dio una charla de evaluación y nos decía “¿qué queréis ser policías o docentes?”. Yo no voy a estar si esto ha sido tanto o cuánto, décimas arriba, abajo… ¿no? Tú evalúate y tal, que yo estoy aquí para enseñarte, para que tú aprendas y no voy a malgastar mi tiempo en hacer una evaluación aséptica y tal, ¿no? Entonces, bueno, tengo muy en cuenta la autoevaluación que se hacen ellos. Si hay una diferencia de más de dos puntos entre lo que les sale, digamos, de la autoevaluación que ellos tienen, los llamo, me pongo en contacto con ellos, y les digo, “vamos a ver, aquí hay problemas de percepción de la realidad que pueden ser míos o tuyos”, y hay veces que incluso me convencen “es que yo he hecho esto y también esto que tú no has tenido en cuenta y tal”, y bueno, es verdad, ¿no? Entonces, claro, hay veces que la evaluación es al final consensuada, pero vamos, que para mí cada tarea tiene su puntuación, su tal y su cual, vamos que pasaría cualquier inspección, tengo ahí mi Excel con un montón de números.

E: Y, me has comentado que utilizas estrategias para fomentar esta relación, ¿no? como lo de los nombres y… ¿quieres añadir alguna más?

P101: Claro, claro, eso es fundamental. Y los que tienen una discapacidad tienes que intentar que tampoco por el hecho de tenerla tengan un trato diferente, porque eso sería un trato discriminatorio incluso para ellos, ¿no? Una confianza excesiva que no le das a ningún otro, ¿por qué? Y después, yo creo que el desafío de la atención a la diversidad está en el coco, ¿no? vamos que es eso, con problemas de depresiones, tal, de pareja, de lo otro… Nos encontramos ahí un batiburrillo de tal, y eso es lo que realmente le afecta a la hora de hacer un seguimiento adecuado de la asignatura, ¿no? y eso, si tú quieres ayudarles, un paso inexcusable es acercarte a ellos. Yo creo que es complicado, ¿no? no podemos tampoco ser psicólogos, ¿no? O sí, no sé. Pero la relación con el alumno es fundamental, y hay una limitación de tiempo, porque además es la parte más bonita, cuando tú te das cuenta de que vas a darle clase a un grupo, que no son bultos, que son personas, conoces sus historias… Yo también me sé de dónde es cada uno, de los pueblos y tal, entonces, tal. Pero yo creo que es también una preocupación sincera, ¿no? “ah, que tú eres de Ronda, ¿y de qué parte?”, y eso, estableces conversación, pero ya no es una conversación pensando en mejorar la docencia, sino que, a ti, como persona, con otras personas vas a compartir un tiempo y te interesa conocerlas, ¿no?

**Documento: 5. Ciencias de la Educación\P102 EDU DISEÑOS**

**Peso: 0**

**Posición: 80 - 85**

**Código: 4. Acciones\Relación profesorado-alumnado\4.15. Importancia relación**

E: Para ti, ¿es importante la relación que se establece entre el alumnado y el profesorado?

P102: Para mí sí.

E: Por qué.

P102: Yo, de hecho, es tanto así que me cuentan sus problemas, sus historias… Que algunos compañeros hasta me han dicho “pero bueno, tú cómo permites que un alumno te cuente cosas de estas”. Yo creo que uno hace tutorías más allá de…hasta me llama el padre de uno o la madre o cosas de estas, ¿no? Me meto más de lo que me llaman, en ese sentido. Creo en esa evaluación sentimental también, esa evaluación del proceso de enseñanza y no solo la evaluación académica.

E: ¿Y qué prácticas o estrategias llevas a cabo para fomentar esa relación?

P102: Yo creo que lo fundamental en este aspecto es la accesibilidad, ser accesible, que el alumno te vea como alguien accesible, a quien le puede preguntar, tener confianza contigo y que sabe que a otra persona no se lo diría y a ti sí. Entonces, primero que sea accesible, que sepa que vas a tener comprensión con sus problemas y que pueda comunicarse contigo más allá de la relación profesor-alumno, que pueda comentarte…

**Documento: 5. Ciencias de la Educación\P103 EDU DISEÑOS**

**Peso: 0**

**Posición: 64 - 67**

**Código: 4. Acciones\Relación profesorado-alumnado\4.15. Importancia relación**

E: Vale. Y ahora sobre la relación alumnado-profesorado ¿Para ti es importante esta relación?

P103: Si hay mala relación profesor-alumno, no hay aprendizaje, no lo hay. Hay otra cosa, hay me lo aprendo porque si no me suspende, porque me da miedo… Pero si quieres que sea educativo, la relación es una buena relación, no un pasteleo, pero una buena relación sí. Porque el alumnado me ve cercano, participativo, y dice “me produce curiosidad, vamos a ver por qué”.

E: Y qué estrategias llevas a cabo para fomentar esta relación con los estudiantes.

P103: Pues mira, primero el campus, fíjate. El primer día que vamos a conocernos les envío correos y tal…el campus es muy importante porque es una manera de relacionarnos. Después, en clase tengo siempre una actitud de escuchar y de preguntar. Y luego, al final de clase me acerco a la gente “qué tal, cómo va la cosa”. Incluso el Facebook, que es una herramienta muy potente para trabajar con los alumnos. Tú lo puedes tener de uso personal o lo puedes tener de vamos a hacer una página donde podamos compartir cosas o incluso tu Facebook personal, que te sirva para comunicarte con los alumnos. Y, a partir de ahí, es algo muy personal, pero algo que da un feedback que no veas. Yo con Mabel hablo mucho por Facebook. Entonces, las redes sociales, bien utilizadas, son un pelotazo, te van dando información y vas viendo si la gente está implicada o no.

**Documento: 5. Ciencias de la Educación\P104 EDU DISEÑOS**

**Peso: 0**

**Posición: 64 - 67**

**Código: 4. Acciones\Relación profesorado-alumnado\4.15. Importancia relación**

E: En cuanto a la relación del profesorado con el alumnado, ¿es importante para ti la relación que se establece?

P104: Sí, mucho. Que el alumno te vea cercano, pero a la vez, establecer los límites y que vean que el experto eres tú, es decir, que no eres el amigo, que no eres el colega. Que hay un dominio, pero también un trato cercano.

E: Te iba a preguntar ahora por las prácticas que llevas a cabo para fomentar esa relación con tu alumnado, pero acabas de decir que la cercanía, ¿no?

P104: Exactamente. Con que te sepas el nombre y en un momento dado le digas…o después de la clase te acerques a él y le preguntes si se ha enterado de todo o si tiene dificultades. Eso ya te va ayudando mucho a que la persona vea que te interesa y que puede contar contigo.

**Documento: 5. Ciencias de la Educación\P105 EDU CREENCIAS**

**Peso: 0**

**Posición: 27 - 27**

**Código: 4. Acciones\Relación profesorado-alumnado\4.15. Importancia relación**

Yo suelto todo, por lo que me van conociendo, saben de mi familia, de mis niños, pongo ejemplos de mi vida personal. Y yo creo que esto es una estrategia didáctica en el sentido de que rompamos la brecha que hay entre lo académico y lo cotidiano. Y creo que se ilustra lo teórico en lo cotidiano.

**Documento: 5. Ciencias de la Educación\P105 EDU CREENCIAS**

**Peso: 0**

**Posición: 41 - 41**

**Código: 4. Acciones\Relación profesorado-alumnado\4.15. Importancia relación**

Yo creo que hay cercanía. Entonces, mi modelo es ese, un modelo de contacto, de contactar con ellos, de remangarme y ponerme a trabajar con ellos, sentado a su altura. Pero, sin perder la posición porque los roles hay que dejarlos claros, pero yo creo que acaban contentos. Yo me veo así, por un lado, un docente que intenta detectar el nivel educativo de ellos, es decir, ver por dónde andan y por dónde van, y por otro lado, el soporte emocional es clave, ya que un grupo es un estado de ánimo, lo tengo así de claro.

**Documento: 5. Ciencias de la Educación\P106 EDU DISEÑOS**

**Peso: 0**

**Posición: 78 - 83**

**Código: 4. Acciones\Relación profesorado-alumnado\4.15. Importancia relación**

E: Y, ahora, sobre la relación entre profesorado-alumnado, ¿para ti es importante esta relación?

P106: Sí. Por lo que te digo, porque yo con los alumnos, en el momento en el que empiezo a poder llamarles por el nombre, ya…también noto que se atreven a preguntar más cuando yo me dirijo a un alumno por su nombre que cuando no…entonces, el tipo de relación. Que tengan confianza también, que puedan preguntar la barbaridad mayor del mundo, que les dices “vale, eso no, pero por qué no”, y ahora, “¿ves cómo lo que me habías dicho no?”, “sí, tal”. Y soy muy machacona, cuando alguien me pregunta, se lo explico y al rato, vuelvo sobre la misma cosa, y vuelvo sobre la misma persona “María, lo que me preguntaste antes, ¿lo ves ahora?”, “sí, sí, ahora lo veo”. Necesito confirmación de ellos continua de que lo están entendiendo, porque si no, luego te encuentras…la clase de hoy, las variables “¿lo tenéis claro?”, “sí, sí”, “vale, ahora hacemos la investigación”, me voy a un grupo y “es que nosotros no entendimos nada de las variables”, y digo “os mato”. Pero, claro, te lo dicen porque están tres.

E: El número de alumnos.

P106: Claro. Cuando son cuarenta no te lo dicen, no preguntan.

E: Claro. Y bueno, esto me lo estabas comentando ya, que qué estrategias llevas a cabo para fomentar la relación con el alumnado.

P106: Pues cercanía. Y, me gusta llamarlos por el nombre, intento aprendérmelos, soy una rompetechos para los nombres, por eso les digo que me pongan el papelito. Luego ya me los voy aprendiendo, normalmente, te aprendes los de los que más preguntan, ¿no? Y, eso, pues depende de la temática, por ejemplo, cuando trabajamos emociones, no se pueden dar detrás de una mesa, entonces, nos vamos todos al suelo y yo con ellos, ¿sabes? La cercanía con los alumnos, que nos vean cercanos. Y las tutorías, les insisto mucho con las tutorías, que, si se quedan con algo que no se queden con ello, que vengan a tutorías.

**Documento: 5. Ciencias de la Educación\P107 EDU DISEÑOS**

**Peso: 0**

**Posición: 138 - 150**

**Código: 4. Acciones\Relación profesorado-alumnado\4.15. Importancia relación**

E: Sí, pero la esencia, la metodología es la misma, lo único que tú haces son ajustes para que la otra persona pueda acceder a la información. Ya ha salido en varias ocasiones y va a sonar repetitivo, pero ¿es para ti importante la relación entre el profesorado y el alumnado?

P107: Es fundamental.

E: Por qué.

P107: Porque yo puedo poner unas presentaciones estupendas, puedo tener un programa docente que es la repanocha y tener un premio por ser el mejor proyecto docente y el mejor programa y todo lo que quieras, pero al final, el alumno tiene que hacer algo con su cabeza para aprender y si tú no garantizas tener las mejores condiciones para que esté dispuesto a aprender, lo puedes contar bonito, pero no lo va a aprender.

E: Y las cuestiones emocionales están ahí.

P107: Claro.

E: Y qué cosas haces o qué prácticas o estrategias llevas a cabo para fomentar esa buena relación, ese buen clima en el aula con todo el alumnado.

P107: Pues hablarles por su nombre, siempre les pregunto por su nombre, no lo recuerdo, pero les pregunto.

E: Yo lo memorizo constantemente.

P107: Sí, yo también. Hablar en plural, por ejemplo, eso es otra cosa. Preguntar directamente cosas personales a ellos, o sea, de lo que piensan, de lo que sienten, a qué se van a dedicar, esa cara qué significa… O cosas personales de “no viniste ayer, ¿qué te pasó?”. Y hablar, y otra cosa es…

E: Mostrar un acercamiento…

P107: Sí. Y no penalizar ninguna intervención, lo contrario, decirles que se tienen que equivocar, que se equivoquen, que no pasa nada. Y no penalizar, ni ridiculizar… Y otra cosa que yo creo que es fundamental es entender quiénes son y dónde están, es decir, entender que a lo mejor lo que a ti te parece super interesante a ellos les parece un rollo, entender que son las tres de la tarde y a lo mejor están dormidos y que tú no puedes utilizar un tono monocorde porque si no, nos dormimos todos…

E: Claro.

**Documento: 5. Ciencias de la Educación\P108 EDU DISEÑOS**

**Peso: 0**

**Posición: 66 - 69**

**Código: 4. Acciones\Relación profesorado-alumnado\4.15. Importancia relación**

E: En cuanto a la relación del profesorado con el alumnado, en la entrevista anterior dijimos que era fundamental la relación entre el profesorado y el alumnado, ¿por qué?

P108: Yo antes te estaba comentando, yo trabajo así y estas estrategias funcionan con todo, en el proceso de construcción del aprendizaje, pero claro, eso conlleva muchas cosas. Eso conlleva que el alumno te respeta, que tú lo respetas, que el alumno te estima, que tiene unas emociones hacia ti y tú también. Otra estrategia que no te he comentado es creer en el alumnado, expectativas altas, yo siempre lo digo, tengo reuniones con mis compañeros y estoy encantada con el grupo, les he dicho que mi grupo es el mejor, yo a ellos también se los digo y ellos comienzan a reírse y les digo oye, no me vayáis a dejar en un mal lugar ¿eh? Que yo pienso que sois los mejores y que pienso que podéis dar mucho, además que sois un grupo magnífico que, además es que yo me lo creo, oye y al final, las expectativas hacen que ellos, digan no me puedo resbalar, no me puedo despistar, eso es otra, pero claro, eso conlleva una calidad humana en las relaciones, con cuestiones emocionales, hay mucha estima, y también el que tu estés bien, por ejemplo, esa es mi forma de trabajar y mi forma de tal, tú has estado saturada o con algún problema fuerte de tal, tu forma de trabajar también se nota, y se ve que tú a lo mejor no estás personalmente, o temas puntuales ¿no? En general trabajas así, pero a lo mejor en un día puntual porque tienes a tu suegro que lo están interviniendo o yo que sé, como me ha pasado ahora en mayo y que es una operación importante y que tú a lo mejor emocionalmente pues estás más triste y tal, entonces empiezas la clase como más baja pero yo no sé también como milagrosamente las clases te sirven a ti como para evadirte y…son a lo mejor los primeros diez minutos los que estás ahí más….Pero ya después esas relaciones que has construido con ellos pues ellos mismos te demandan y tú te olvidas del problema, es una cosa tremenda. Entonces yo creo que el potencial está en esa calidad de las relaciones de simetría siempre en una relación…A ver, son relaciones horizontales, pero con un poquito de asimetría porque verás yo tengo un papel, son relaciones cercanas, pero con un poco de límites y que cuando hay algo que se va un poco de tal pues tú tienes la responsabilidad de retomar, y de resituar, pero son relaciones basadas en el respeto, en el cariño, en la creencia de los otros, de los que tienes en frente.

E: ¿Y qué prácticas llevas a cabo para favorecer las relaciones con el alumnado?

P108: Pues, las que te he comentado, tener relaciones cercanas con ellos, a veces si estás dando la clase y les haces una broma. El clima, cuidar mucho el clima y esas relaciones con ellos, que ellos se sientan libres para hablar y comentar, contarte sus cosas, y yo veo que en mis clases en general funciona. Hombre, siempre tienes al alumno que no participa y que te cuesta la propia vida que participe, a lo mejor no participa porque es muy tímido, pero después en las prácticas en “petit comité” tú le dices, oye, esta primera práctica a penas has intervenido, eso lo tienes que mejorar, en la siguiente espero que tú participes y es super gratificante cuando a esa persona la escuchas hablar, y participa y trabaja bien y sobre todo, no porque tú no esperaras que fuera igual que los demás, sino sobre todo por haber roto el hielo, y que esa persona a pesar de la timidez tan extrema que tiene haya sido capaz de exponer, de hablar, de estar tranquilo, de hacerlo en un clima. Entonces, yo creo que son estrategias de componente humano y además, veo que funciona porque ellos a veces en clase me dicen ¿puedo poner un ejemplo? O me preguntan en voz alta porque yo desde el momento cero les digo que tienen que participar y que yo también tengo que aprender de ellos, y que aquí venimos todos a aprender y a contar, entonces cuando ellos ven que yo cuento muchas experiencias y muchas cosas mías pues ellos también ponen los suyos y, a veces, por eso incluso me piden consejo de problemas que ellos tienen incluso con otras asignaturas, dudas de otras asignaturas, que yo a veces me río y digo, la verdad que es muy gratificante que confiéis en mí, pero también que sepáis que las tutorías están y que también podéis hablar con los demás profesores aunque vosotros de entrada los veáis más serios y más tal pues que podáis hablar y negociar las cosas…También veo yo que el clima, ese clima es una estrategia muy buena para aprender.

**Documento: 5. Ciencias de la Educación\P109 EDU DISEÑOS**

**Peso: 0**

**Posición: 132 - 149**

**Código: 4. Acciones\Relación profesorado-alumnado\4.15. Importancia relación**

E: Para ti, pasando ahora a hablar de la relación entre docente y alumnado, ¿es importante la relación que se establece?

P109: A mí me lo parece, considero que es muy importante.

E: Por qué.

P109: Yo es que pienso que, en la enseñanza, ese vínculo que se establece profesor-alumnado, tiene un peso muy importante sobre el resultado del proceso. La imagen o el modelo, la opinión o percepción, no sé cómo llamarlo, que tienes en la confianza de lo que tú me puedes aportar como docente, yo creo que es fundamental, es decir, que lo que tú me dices, a mí me toque, pero no solo que me toque desde el punto de vista cognitivo o intelectual, que me toque mis esquemas, sino que me toque a nivel personal, es decir, que yo me lo estoy creyendo, como persona. Como yo les digo, por ejemplo, el tema del género, y otros temas actuales, yo no quiero que te sepas solo qué distintos tipos de relaciones familiares hay, porque como van a ser maestros, quiero que, personalmente, te convenza, de que esto es así, es importante, hay que abordarlo, y que, si no tienes esta opinión, tenemos que trabajarlo. Entonces, para que tú no toques solo lo cognitivo, sino lo personal, tenemos que hacerlo.

E: Muchos contenidos de reflexión y…

P109: Exactamente. Yo creo que crear una buena alianza con unos límites ayuda, porque el alumnado es como que se entrega, yo tengo la sensación de como que están entregaditos, es que les diga lo que les diga…

E: Porque cuando tú creas ese clima de partida, ya hemos hecho gran parte del camino.

P109: Muchas veces a mí me da miedo porque, eso se lo digo algunas veces, digo “a ver, que no os quiero adoctrinar, que no quiero que vuestras ideas sean las mías”, porque a veces los ves entregaditos y tú dices “simplemente quiero que entendáis que vuestras ideas tienen que estar, como futuros maestros, al amparo de lo que la literatura y la investigación tiene”, porque a veces tú los ves como reproduciendo lo que tú les acabas de decir y dices…

E: O que piensan como cualquier persona de la calle.

P109: Claro. Entonces, a mí me da miedo, porque no es adoctrinamiento, es un modelo.

E: Claro, lo que pasa es que ellos tienen que, en los contenidos de aprendizaje, hay que aprenderlos, hay que reflexionar, y que en una profesión como es educador, tú previamente tienes que revisar tus planteamientos de partida.

P109: Exactamente.

E: Eso es igual que si partes de un modelo teórico, es que la reflexión…

P109: Claro, y yo con lo que tú estabas planteando, es que, para hacer eso, hay que crear un vínculo, o sea, la relación tiene que ser buena.

E: Y tú que haces, qué estrategias o qué prácticas llevas a cabo para fomentar esa relación con tu alumnado.

P109: Pues yo te voy a decir, yo soy como soy, quiero decir, que soy natural con ellos. Es verdad que, hombre, que intento ser…y mira que soy muy directiva, o sea, que yo marco mucho lo que hay que hacer…intento llegar a un punto de una distancia emocional ajustada, ¿vale? Es decir, una cercanía, intentar que ellos sientan que yo soy accesible, pero que también sientan que soy un referente teórico, conceptual, un modelo, ¿no? Y, ¿cómo? Porque a lo mejor tiene mucho que ver con mi forma de ser, ¿no? Yo les ofrezco un espacio donde los hago partícipes, donde no cuestiono, donde pueden hablar libremente, ¿no? Dentro de…bueno, respetando los límites, soy amable, que ellos sientan que, bueno, que el entorno es amable. Por supuestísimo, soy educada con ellos, siempre dándoles una cierta cercanía, ¿no? Yo intento que el aula, ese espacio, sea un entorno amable, ¿sabes? Que ellos entren…es verdad que ellos “ay, estamos cansados”, bueno, pues…incluso cuando “estamos cansados, que acabamos de salir de matemáticas”, pues yo intento “qué habéis dado” …no sé, crear un entorno donde, como me decía un alumno, “es que nos mete cada tocho y no nos damos ni cuenta”, claro, es decir, ¿no? O como dice mi hijo “al final me estás dando una de órdenes que yo no me he dado cuenta”, ¿no? O sea, tú me estás diciendo esto de una manera…pues…

E: Sensible.

P109: Exactamente, ser sensible. Esa sensibilidad, esa empatía hacia ellos, entenderlos, escucharlos… Por ejemplo, en mi aula ellos pueden hablar e incluso de algún compañero y yo jamás…les pido que no me den nombres, jamás…yo no les permito que me digan nombres ni que falten al respeto, pero bueno, los escucho y les animo a solucionarlo de manera constructiva y les digo que vayan a esa persona, que no quiero saber su nombre, porque es un compañero o compañera más…pero que ellos sientan que es un entorno desde lo amable, lo correcto, lo educado, y la empatía.

**Documento: 5. Ciencias de la Educación\P110 EDU DISEÑOS**

**Peso: 0**

**Posición: 96 - 103**

**Código: 4. Acciones\Relación profesorado-alumnado\4.15. Importancia relación**

E: Y, ¿para ti es importante la relación, me la vas a contestar que sí, entre alumnado y profesorado?

P110: Es que es la base de la enseñanza, creo que, si no estableces una buena conexión, en psicología lo llamamos raport, no sé…

E: Sí, raport.

P110: Si no estableces una buena conexión, un buen raport, da igual lo que expliques, no van a aprender, no van a aprender, no hay más, luego, habrá que currárselo, que no consiste en hacerte súper coleguita, ni en, de repente volverte moderno, ni…consiste en respetar a los alumnos, comprender a los alumnos, ayudar a los alumnos y pedirles que hagan un acto de reciprocidad, que nos respeten como docentes, que nos comprendan, que nos ayuden.

E: Y qué prácticas o estrategias llevas a cabo para fomentar la relación con tus alumnos.

P110: No soy consciente de no hacer nada queriendo, que a lo mejor lo hago, pero no soy consciente. Desde el principio sí obligo a la gente a, quiero que me digáis las cosas, quiero que participéis y, evidentemente, si le pides la opinión, no es para castigarles, que eso es una cosa que como docente nos encanta, te digo que me digas lo que no funciona en mi asignatura y después me enfado contigo cuando me lo dices, ¿somos tontos?, si te he preguntado es para hacerte caso, si no, no te pregunto, e intentar también, tener consideración individual con ellos, preguntarles cómo vas, cómo te sientes, cuando ves a uno con la mala cara, “oye, qué te pasa, ¿te puedo ayudar?”, es que creo que consiste en tener un trato humano, si hago otra cosa no soy consciente de ello.

E: Y, ¿cómo crees que esa relación puede influir en el aprendizaje?

P110: Es el principio, y el medio, y el fin. Si tienes una buena relación con un alumno, los alumnos para empezar, están más motivados con tu asignatura, para continuar, no tienen miedo a hacer preguntas, y si no preguntas, no aprendes, para seguir, me ha pasado alguna vez, que me diga un alumno, “P110 no me voy a presentar a tu examen”, “¿por qué?”, “Porque me da vergüenza suspender contigo y no he estudiado”, creo que al final, eso también es un arma, el, no quiero fallarle a esta persona, bueno, pues si es un arma, habrá que usarla, como cualquier otra.

**Documento: 5. Ciencias de la Educación\P111 EDU DISEÑOS**

**Peso: 0**

**Posición: 52 - 59**

**Código: 4. Acciones\Relación profesorado-alumnado\4.15. Importancia relación**

E: Pasamos al área de las relaciones entre el profesorado y el alumnado. Entonces, ¿para ti es importante esa relación?

P111: Sí, yo soy muy sensible a esta relación. Necesito saber que ellos siguen las cosas. Lo que yo llevaría peor, es pensar que estoy hablando a la gente de estadística y que no entienden nada. Entonces, soy muy sensible al feedback que me den. También, puede haber alumnos que no se enteren o que tengan cara de póker y entonces, durante las clases teóricas, pues es necesario que pongas ciertos ejercicios de lo que acaban de ver a realizar en clase y que lo hagan por pareja, por ejemplo, en los últimos diez minutos. Entonces, aquí es donde voy viendo si han asimilado los contenidos y estas cosas.

E: Además, me has dicho antes que suelen ser participativos, entonces esto te da a ti un feedback de que se están enterando.

P111: Sí. Es verdad, que hay un grupo de gente que participa, pero es verdad que el grueso del alumnado es gente que no suelen participar mucho, pero bueno siempre hay gente que sí, que participan. Este año he tenido un alumno con discapacidad de movilidad y participaba mucho y yo le he agradecido al final de curso las aportaciones que ha hecho. De igual forma, también se lo he agradecido a dos alumnos más que no tenían discapacidad, ya que gracias a estos tres alumnos, el resto de la clase participaba algo más.

E: ¿Qué estrategias llevas a cabo para fomentar esta relación con el alumnado?

P111: Pues, me pasa un poco como antes. Es que no tengo estrategias explícitas, sino las habilidades sociales que uno puede tener en clase. Yo soy incapaz de echarles una bronca a los estudiantes, soy muy blandito en ese sentido. Pero también es verdad, que ellos me facilitan mucho las cosas. O sea, que no necesito ser un policía…y creo que estas cosas ellos lo valoran. Es que estas cosas hacen que ellos se sientan cómodos.

E: La relación es un poco más simétrica.

P111: Sí, yo creo que se sienten cómodos. Pero, claro yo no les doy una relación de colegas ni nada.

**Documento: 5. Ciencias de la Educación\P113 EDU DISEÑOS**

**Peso: 0**

**Posición: 20 - 21**

**Código: 4. Acciones\Relación profesorado-alumnado\4.15. Importancia relación**

E: Pues ya hemos hablado un poco de la planificación de la guía docente. Ahora, quiero que nos cuentes qué aspectos consideras que son los más importantes a la hora de poner todo esto en el aula, no sé que funcionen bien los dispositivos electrónicos, que haya un buen ambiente de clima de aula, etc.

P113: Tiene que haber un buen ambiente en el aula, pero esto no significa una relación de amistad entre docente-estudiante. Creo que el docente es docente y el estudiante es estudiante. Pero, sí te tienes que sentir con libertad docente para poder preguntar a nivel docente.

**Documento: 5. Ciencias de la Educación\P113 EDU DISEÑOS**

**Peso: 0**

**Posición: 56 - 59**

**Código: 4. Acciones\Relación profesorado-alumnado\4.15. Importancia relación**

E: Vale. En cuanto a la relación que tiene el profesorado con el alumnado, para ti, ¿es importante esa relación?

P113: Sí, el profesor tiene que intentar que el alumnado venza a ciertos miedos, como por ejemplo, a que hable en público, a través de exposiciones, así como el profesor tiene que incentivar que el alumnado pregunte, haga consulta.

E: ¿Qué prácticas o estrategias llevas acabo para fomentar esta relación?

P113: Pues, preguntar una y otra vez lo mismo en el aula para que vaya cogiendo lo que se espera de ellos, que es que ellos respondan y esto es muy difícil de conseguir.

**Documento: 5. Ciencias de la Educación\P114 EDU DISEÑOS**

**Peso: 0**

**Posición: 62 - 65**

**Código: 4. Acciones\Relación profesorado-alumnado\4.15. Importancia relación**

E: Vale. Me has hablado antes sobre la relación que hay entre el profesorado y el alumnado, sobre la importancia que tiene esta relación. ¿Por qué consideras que es tan importante?

P114: Pues porque somos personas, quiero decir, a ver, nosotros ahora, ¿vale?, esto lo podríamos haber hecho escribiéndonos, o lo que sea. El hecho de que nos estemos viendo establece una relación más cercana, más directa. Entonces, cuando estás interaccionando con el alumno, él aporta sus características y tú aportas las tuyas, y eso es inevitable. Entonces, si yo me limito a dirigirme a un aula, con un contenido, y echarlo, eso lo hace cualquier robot. Sin embargo, si sigue habiendo en las escuelas un maestro, un profesor o un docente, es porque se crea algo más que una simple transmisión de contenidos. Tiene que estar la interacción, entonces, puedes tener con unos un poco más de empatía, con otros no, pueden tener más carácter, más de esto, pero lo normal es que cuando hables, el otro vea interés por el aprendizaje y por el alumno.

E: Vale, y qué prácticas o estrategias llevas a cabo para fomentar ese feedback, esa relación con el alumnado.

P114: Pues desde el principio, fomentar eso, que vean que yo pregunto, si no contestan, a lo mejor, con ironía o con broma, “qué pasa, ¿es que no lo sabéis?, venga, yo os ayudo, tal”, o cuando a lo mejor, alguien participa, valorarle que ha participado, o si no le escuchan, pues decir, “oye, ya le está costando a ella, a esta persona, el esfuerzo de hacerlo, que da corte”, pues ayudarla, o si alguien intenta responder pero se queda en duda, pues decir, “venga, alguien que le ayude”, un poco así…

**Documento: 5. Ciencias de la Educación\P115 EDU DISEÑOS**

**Peso: 0**

**Posición: 76 - 83**

**Código: 4. Acciones\Relación profesorado-alumnado\4.15. Importancia relación**

E: En cuanto a la relación profesor y alumnado. ¿Para ti es importante la relación que se establece entre el alumnado y el profesorado?

P115: Sí. También es verdad que no nos tenemos que hacer amigos, sino un término medio. Hay que estar próximos, pero teniendo claro cuál es nuestra labor.

E: ¿Por qué crees que es importante mantener estas relaciones?

P115: Para llevar a cabo un seguimiento y, además, te da un feedback para saber si están entendiendo las cosas, sí les gusta, sí no les gusta. Yo creo que aprendemos todos, nosotros sobre como abordar la práctica docente, y ellos, pues aprenden contenidos y habilidades.

E: ¿Qué prácticas o estrategias llevas a cabo para fomentar la relación con tu alumnado?

P115: Yo creo que cuando me dirijo a ellos y demás, yo soy próxima, pues eso facilita que ellos me pidan cualquier cosa que necesiten. Yo es que no hago nada en especial, pero sí es cierto que no tengo una forma de ser distante…todo sale de forma natural.

E: No utilizas estrategias concretas, pero por tu personalidad o tu forma de ser sí que…

P115: Yo les voy preguntando si tienen dudas o si alguien está interesado en un tema que no vamos a ver o que quiere ver con más profundidad, pues que me lo diga y le ofrezco el material. Así que siempre facilito el contacto.

**Documento: 5. Ciencias de la Educación\P116 EDU DISEÑOS**

**Peso: 0**

**Posición: 66 - 71**

**Código: 4. Acciones\Relación profesorado-alumnado\4.15. Importancia relación**

E: Y ahora me gustaría preguntarte por la relación docente-alumno. Entonces, bueno, la primera pregunta es un poco obvia, pero tengo que preguntarte si para ti es importante esta relación.

P116: Sí es importante, pero claro, hay que tener unos ciertos límites. Vamos a ver, la figura del profesor y el alumno, aunque eso no impida que pueda haber una relación cordial y además yo te comentaba el otro día que esto es fundamental. Con el paso de los años sí que hay esta…es como si todavía estuvieran en el instituto, no es diferente la universidad, ¿no? Depende más de su propia motivación o de su propio trabajo y no tienen que ser tan guiado, no porque la asignatura no tenga que ser guiada, sino que se tienen que organizar ellos mejor el tiempo, las asignaturas… Tiene que ser una realidad diferente a lo que tenían en el instituto, eso sí. Y yo lo que veo es que al menos una parte de los estudiantes que llegan nuevos, como tengo estas dos asignaturas de los primeros años, sí que veo que les cuesta un poquito más, lo tenían asumido, yo creo que antes.

E: Sí, que ahora mismo están todavía un poquito fuera de lugar.

P116: Sí, sí.

E: Y, para fomentar la relación tuya entre tú y los alumnos, qué cosas sueles hacer o…

P116: La verdad es que…lo que pasa es que ya, como te comenté el otro día, por mis características de la personalidad, ya, digamos que no hay esa cuestión real, no.

**Documento: 5. Ciencias de la Educación\P117 EDU DISEÑOS**

**Peso: 0**

**Posición: 46 - 49**

**Código: 4. Acciones\Relación profesorado-alumnado\4.15. Importancia relación**

E: Claro. Y si hablamos ahora de la relación profesor-alumno, me gustaría preguntarte, si para ti es importante esa relación y por qué.

P117: A ver, sí, para mí es importante la relación, entendiendo que nunca se pierde de vista quién es el profesor y quién es el alumno. Como comentaba antes, no tienen ningún problema para acceder a mí, saben cuál es mi despacho, saben mi horario de tutorías, saben que pueden venir cuando quieran, incluso cuando no es el horario de tutorías, saben que pueden contactar conmigo a través del aula virtual, por email, de cualquier manera. Y la idea es que ellos entiendan que yo estoy ahí para facilitarles el adquirir una serie de competencias, ya sea de contenido, de procedimientos, de lo que sea, en relación con la materia, de la cual soy responsable y que a mí me toca impartir. Entonces, para mí es importante que los alumnos no se queden con una pregunta o con un comentario porque me vean inalcanzable o me vean que soy desagradable o eso. Entonces, yo intento ser lo más abierta posible, pero sin olvidar que la relación es: yo soy la responsable de la asignatura, yo puedo facilitarles la asignatura, las partes formales, las partes de contenido, y que esto no se entienda mal, pero que nunca vamos a discutir sobre la asignatura desde el mismo nivel, porque no estamos en el mismo nivel. Ellos están para aprender la asignatura y yo la tengo controlada desde su nivel y desde más allá. Pero sí que es muy importante el que sepan que no tienen que enfrentarse, en mi caso particular, al profesorado ni con miedo, ni con reservas, ni con “a ver si pregunto esto y me va a tratar de ignorante”, esto nunca jamás, yo siempre lo he contado así “vosotros estáis para aprender, cualquier duda que tengáis me la formuláis”. Que yo lo entiendo desde ese punto de vista, yo entiendo que cuando alguien me pregunta algo, no es por falta de interés, no es porque quiera ponérmelo a mí difícil, es porque simplemente no lo entiende, y yo comprendo eso, entiendo que haya algún concepto que yo no haya transmitido bien y que me puedan preguntar lo que sea. Entonces, yo creo que tengo una buena relación con los alumnos, que no tienen ningún problema en venir a decirme tanto las cosas que funcionan como las que no funcionan, y en el aula nunca jamás he tenido ningún problema con ningún estudiante. Son muchas horas de docencia, sí que recuerdo alguna vez que un estudiante se dirigió con muy poco respeto, le contesté y me dirigí tajante, pero respetando…y en los veinte y pico años que llevo dando clase solamente ha habido una vez que eché de clase a tres estudiantes, que estaban armando tanto barullo que incluso los propios compañeros se estaban quejando. Entonces, la única vez en veinte y pico años significa que está bien, que no es difícil mantener un ambiente ideal para que todos puedan seguir la clase y luego siempre el respeto con respecto a los estudiantes, eso nunca ha sido un problema.

E: Vale, y para fomentar que haya una buena relación, qué estrategias utilizas, les comentas “estoy aquí para lo que queráis”, ¿alguna cuestión más?

P117: No, sobre todo, en las explicaciones, las caras lo dicen todo. Cuando estás explicando y tienes de frente a toda esta gente ves los que están apuntando bien, los que están tomando apuntes, pero se les ve agobiadísimos, los que están más mirando el móvil que otra cosa… Entonces, tú tienes que…son años también, ¿vale? Tienes que ver esto se está entendiendo y esto no, y muchas veces lo digo previamente “esta es una de las clases más fáciles de dar” o directamente “yo lo sé, porque es un tema muy árido, que hoy os va a ser un poquito más difícil de seguir”, y entonces voy más tranquila, voy parándome más, voy preguntándome más frecuentemente “¿esto lo sabe o no?”, y preguntándoles a ellos y viendo las caras. Entonces, muchas veces, y esto me lo reconozco, le facilito que me hagan las preguntas, o sea, aparte de ellos saben que me pueden preguntar y cuando me han preguntado un par de estudiantes ya ven que les contesto bien, sin ningún problema y repitiendo las cosas, y entonces, creo que eso les quita el miedo que puedan tener a levantar la mano o hacer una pregunta, porque muchas veces son ellos mismos los que dicen “uy, esto será una tontería y se van a reír”, y entonces, no hay ningún problema, de hecho cuando ha ocurrido que alguien pregunta algo y el resto se ríe, se controla rápidamente “pues me parece muy interesante la pregunta”, a ver, lo puedes controlar relativamente bien, a veces soy yo la que les pregunto y es “¿esto se ha entendido?”, y cuando ves muchas caras, pero nadie dice nada, dices “esas caras me están diciendo que no, a ver, tú que…”, “pues yo no he entendido esto”, y les facilitas que lo puedan preguntar. Por lo que decía antes, yo puedo tener una forma muy clásica de dar la clase, el rol de tal, tal y tal, y de explicar los contenidos, pero eso no significa que sea un rol autoritario. Los alumnos pueden participar en el momento en el que quieran, y si alguna vez alguien se lanza a hablar o no levanta la mano o tal, simplemente lo paro y digo “déjame que acabe de hablar y ya está y cuando acabe puedes formular tu pregunta”, y no hay ningún problema ni ninguna mala dinámica ni ninguna forma de dar la clase que impida que si los alumnos se quedan con alguna duda no se vean con la libertad de poderla transmitir. Y yo siempre repito lo mismo, no solamente, como tiene que ser, no es que respete lo que ellos me preguntan o que ellos me respeten a mí, sino que también tengo muy en cuenta el respeto entre los distintos compañeros. Siempre se generan roles dentro de la clase y siempre hay gente que pregunta mucho, a veces, sin mucho sentido, y al final esto genera un poco de…el resto se cansa, es muy evidente, se ríen, pero esto se intenta controlar, que no se generen dentro de la clase como grupitos o que no “ya está este otra vez preguntando esas superioridades”, está todo bastante controlado y de eso no tengo quejas cuando evalúo, por eso creo que lo tengo bastante bien en la dinámica de la clase.

**Documento: 5. Ciencias de la Educación\P118 EDU CREENCIAS**

**Peso: 0**

**Posición: 19 - 19**

**Código: 4. Acciones\Relación profesorado-alumnado\4.15. Importancia relación**

P118: Bueno, me manda un correo de estos de agradecimiento que casi te hacen llorar y esas cosas, pero la verdad es que mi relación con los estudiantes suele ser muy buena. Aquí se ríen mis compañeros porque dicen que tendría que montar una consulta. Pero bien.

**Documento: 5. Ciencias de la Educación\P118 EDU CREENCIAS**

**Peso: 0**

**Posición: 47 - 47**

**Código: 4. Acciones\Relación profesorado-alumnado\4.15. Importancia relación**

Para mí la relación entre el estudiante y el docente es sagrada, y las clases son sagradas. Entonces, yo siempre les digo que si no tienen nada que aportar que no vengan.

**Documento: 5. Ciencias de la Educación\P118 EDU CREENCIAS**

**Peso: 0**

**Posición: 49 - 49**

**Código: 4. Acciones\Relación profesorado-alumnado\4.15. Importancia relación**

Pero yo creo que, digamos, establecemos una relación personal, a veces, y tengo que frenarla a veces, ¿eh?, pero estoy cerca de ellos, y ellos están…no hay mucha distancia entre ellos y yo.

**Documento: 5. Ciencias de la Educación\P118 EDU DISEÑOS**

**Peso: 0**

**Posición: 132 - 137**

**Código: 4. Acciones\Relación profesorado-alumnado\4.15. Importancia relación**

E: Vale. Y ahora vamos a hablar un poquito de la relación del profesorado con el alumnado. Entonces, la primera pregunta, que es muy obvia, si para ti es importante la relación que se establece y, ¿por qué?

P118: Para mí es básica. Es básica. Desde la cercanía yo me puedo permitir las licencias, a veces, de...pero primero tengo que...nosotros tenemos...explico yo una teoría de liderazgo que se llama la teoría del crédito idiosincrático que dice que de una forma u otra los profesores somos una especie de líderes en las clases. Entonces, esa teoría siempre dice que un líder nunca puede introducir cambios al principio, cuando llega. Al principio tiene que ser uno más. Pero ya cuando ha conseguido crédito, es decir, la confianza y ahí les explico la diferencia entre préstamo y crédito... que no lo sabía, pero me enteré cuando entré a la UJI, luego te lo contaré. Cuando ha conseguido el crédito, es decir, la confianza, entonces a partir de ahí ya puede...le llama idiosincrático porque el momento es...idiosincrasia es puntual, lo puede utilizar. Entonces, puedes llevarte...el teórico este dice que el líder se puede llevar al grupo donde quiera, pero previamente se ha ganado la confianza. Entonces, para mí es básico. Es básico porque desde esa confianza que se establece entre los dos...pero yo también, siempre les digo que...eso te lo conté el otro día, lo del liderazgo transformacional, ¿no? Que yo soy una líder transformacional. Lo que digo es que yo procuro comportarme por lo menos así. Yo les digo siempre “no os voy a tratar a todos por igual. Si alguien espera eso, lo siento. Pero no lo vamos a tener porque no es...porque no”. Entonces, como uno de los teóricos más importantes de la asignatura establece la diferencia teórica y lo tienen que aprender entre relación grupal e interpersonal...yo digo “podemos relacionar profesor y alumno. Perfecto. La norma es la norma. Entre todos somos genial para todo el mundo. Me es igual, Luis, que tú mañana tengas examen de inglés a la misma hora que el examen de grupos. Tienes que venir a hacer el examen de grupos. Pero si me relaciono interpersonalmente te diré “ven a mi despacho y te haré el examen antes o después. O con un cuatro con noventa y nueve te suspenderé y si me voy a la interpersonal te aprobaré, a lo mejor, con un tres””. Entonces, hablamos...trabajamos eso. Entonces, yo me sitúo ahí. Pero porque me lo creo. Quiero decir, para mí es básico. Aunque una vez un estudiante me dijo...hablábamos de eso y un estudiante me dijo “pues yo no estoy de acuerdo. Yo no he venido aquí para que los profesores sean simpáticos o me comprendan. No quiero ni que me saluden en el bar”. Ahora es compañera mía de departamento. Nos dejó a toda la clase...pero una estudiante en psicología de la comunicación decía “claro, porque la UJI una de las ventajas que tiene es que somos grupos pequeños, hay una cercanía y tal”. Y aquella nos sacó del sistema, cambió radical. Pues vale, chica, pues vale. Lo que pasa es que eso hace que llegues a situaciones en las que...pues a veces me veo en situaciones en las que “¿de verdad tengo necesidad de esto?” Porque me ha pasado de todo. Pero para mí es básico.

E: Claro. Y tú, ¿qué estrategias utilizas para que se fomente esta relación?

P118: Yo no creo...es que ninguna, Ana. Si fuera una estrategia, no saldría.

E: O, ¿qué cosas haces? No sé, ¿Lo que surge?

P118: Lo que...yo estoy un poquito “loqueta”, que me llaman ellos. Y yo creo que es que a mí me interesa la gente. Yo, cuando tengo a alguien delante...pero no es ningún mérito, es...nací así. Quiero decir, es algo que viene conmigo. Y lo que hago es atenderles. En la manera de lo posible, me pongo en su pellejo. Quiero decir...y a veces me mosqueo mucho, ¿eh? Pero yo creo que es porque me interesan. Y entonces, a partir de ahí sale solo. Lo que pasa es que a veces se mosquean. Algunos se han mosqueado conmigo. Pero yo ya les he dicho que no...y eso me ayudó mucho la jefa de titulación un día, porque no sé qué le dije, y me dijo “una cosa es poner notas y la otra evaluar”. Y digo “vale, ya estoy tranquila”. Y yo, este año, a un estudiante con un dos y medio, uno de los cuentos, es que yo no lo podía suspender, había tenido una historia fea, la había dejado la novia. Estaba hecho una mierda el pobre. No había faltado ni un día, era súper participativo y en los “cigarrets” que es algo muy importante en mi clase, los “cigarrets”, era el tío...digo “yo no lo puedo suspender”. Pero eso solamente es posible desde la confianza. Ahora, yo le dije “tío, ni se te ocurra, ¿eh? Hacer publicidad de esto”. Que a mí no me importa, que luego lo digo yo, pero para que no lo comenten por ahí. Pero ellos saben que eso puede pasar porque desde el primer día lo he avisado. O una vez que tenía a un estudiante que su padre tenía una novia y le había puesto en el móvil el nombre de la hija.Y estaba en la habitación recibiendo los mensajes de su...Y vino hecha una mierda. Una mierda. Que qué hacía, si se lo decía a su madre o no. Y yo...digo “¿qué coño se lo va a decir a su madre?” Claro, yo digo “pues esta chica hará lo que yo le diga. Me acojoné”. Pero tuve que pensar. Y yo le dije “no, eso es una relación de tu madre y tu padre, tú ahí...tú habla con tu padre que es el que te está mandando los mensajes”. Pero vino a revisión de exámenes y se desmoronó. “¿No me estará mintiendo?” Es que era imposible. Me enseñó de todo. Pues con un cuatro y medio dije esta pasa. Tú imagínate el plan. Vino en septiembre “P118, te hice caso. No le dije nada a mi madre. Y se han arreglado”. Digo “pues claro, que se apañen ellos”. Pero eso sale desde la confianza. Pero es porque me suelo llevar bien.

**Documento: 5. Ciencias de la Educación\P119 EDU DISEÑOS**

**Peso: 0**

**Posición: 58 - 63**

**Código: 4. Acciones\Relación profesorado-alumnado\4.15. Importancia relación**

E: Vale. En cuanto a la relación del profesorado con el alumnado, ¿para usted es importante esa relación?

P119: Sí. Yo considero que a raíz de esta relación, pues el estudiante se siente más atraído por lo que le vas a presentar.

E: Vale. ¿Qué medios utilizas para comunicarse con su alumnado?

P119: Pues utilizo la plataforma, ya que tenemos un servicio de mensajería, y luego hay un sistema similar al de WhatsApp, dependiendo, vamos a ver, si hablamos de grado, de estudiantes de grados, pues utilizo una aplicación que se llama Remark, es un aplicación que te permite comunicarte unidireccionalmente. Tú comunicas a todos y ellos se pueden poner en contacto contigo a nivel individual. Entonces, esto es una información que ellos agradecen porque es inmediata, en un momento puntual que precisa de una ayuda. Luego, con los estudiantes de máster o de doctorado, genero siempre grupos de WhatsApp para solucionar problemas conjuntos o situaciones directas.

E: Muy bien. ¿Cómo valoras su relación con los estudiantes?

P119: Pues, una relación normal.

**Documento: 5. Ciencias de la Educación\P119 EDU DISEÑOS**

**Peso: 0**

**Posición: 66 - 67**

**Código: 4. Acciones\Relación profesorado-alumnado\4.15. Importancia relación**

E: ¿Consideras que se debe cambiar el modo en el que el docente se relaciona con su alumnado?

P119: En principio, creo que se debe ser más sensible al estudiante, estar más próximo a él, pero sin perder la orientación tampoco.
